# Stereodivergent Palladium-Catalyzed C–F Bond Functionalization of *gem*-Difluoroalkenes

**DOI:** 10.1021/acs.orglett.4c02112

**Published:** 2024-06-27

**Authors:** Yanhui Wang, Gavin Chit Tsui

**Affiliations:** † Department of Chemistry, The Chinese University of Hong Kong, Shatin, New Territories, Hong Kong SAR 999077, China; ‡ Shanghai-Hong Kong Joint Laboratory in Chemical Synthesis, Shanghai Institute of Organic Chemistry, The Chinese Academy of Sciences, Shanghai 200032, China

## Abstract

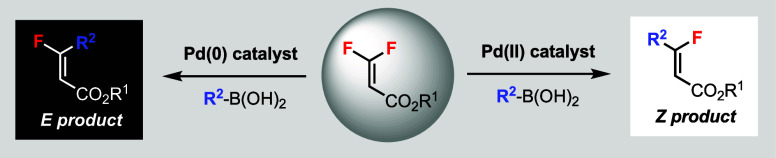

We herein describe a stereodivergent C–F bond functionalization of *gem*-difluoroalkenes. Using trisubstituted β,β-difluoroacrylates, both *E* and *Z* monofluoroalkene products can be obtained with excellent diastereoselectivities. The design of two different reaction manifolds, i.e., Pd(II)- versus Pd(0)-catalyzed cross-coupling of boronic acids, is the key to stereocontrol.

*gem*-Difluoroalkenes are privileged motifs in biological and synthetic applications.^1^ The two strong electron-withdrawing F atoms through the σ-bond can activate the alkene for nucleophilic attack. This fundamental property allows the *gem*-difluoroalkenes to serve as precursors for a broad spectrum of organic transformations. One of the most important synthetic applications of *gem*-difluoroalkenes in recent years is the selective transition metal-catalyzed C–F bond functionalization^2^ for the synthesis of monofluoroalkenes. Monofluoroalkenes are of particular importance in medicinal chemistry because of their potential to act as amide bond isosteres and enol mimics.^3^ Thus, the utility of monofluoroalkenes can be found in pharmaceutical development, materials science, and organic synthesis.

In general, the carbon–fluorine bond transformation is considered much more difficult than C–I, C–Br, and C–Cl bonds because of the enormous bond dissociation energy (∼120 kcal/mol) of C–F bonds.^4^ However, in the presence of transition metal catalysts [M] (M = Cu,^5^ Ni,^6^ Rh,^7^ Co,^8^ Mn,^9^ Ru,^10^ Pd,^11^ and Fe^12^), *gem*-difluoroalkenes can undergo selective functionalization of a single C–F bond. The key step in these reactions is the β-fluoride elimination of the β-fluoroalkylmetal intermediate (Scheme 1a).^1a^ The formation of a strong M–F bond is the thermodynamic driving force for this process. The diastereoselectivity usually favors the *Z*-monofluoroalkene product.

**Scheme 1:**
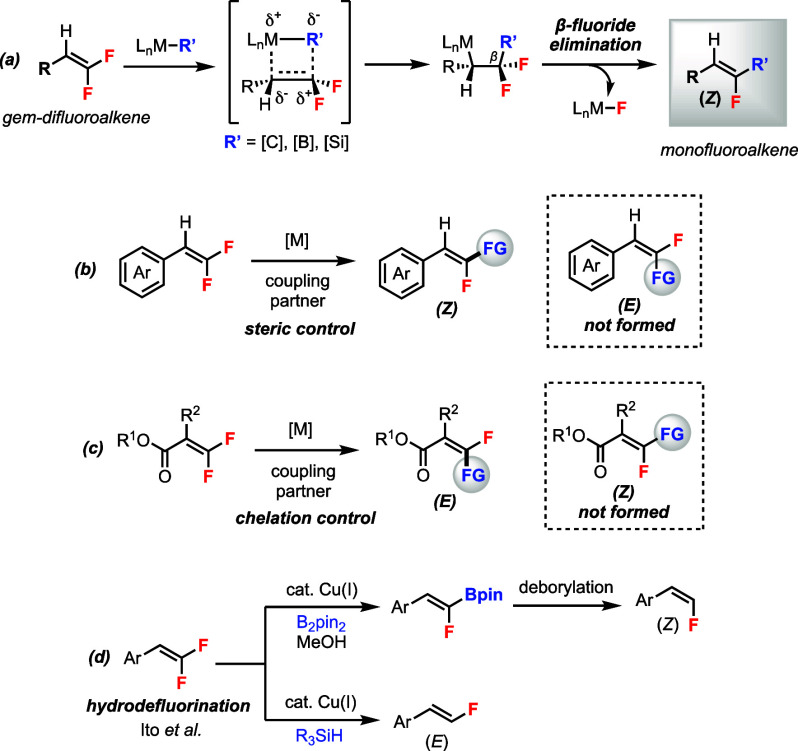
Transition-Metal-Catalyzed C–F Bond Functionalization of *gem*-Difluoroalkenes

Currently, transition-metal-catalyzed C–F bond functionalization of *gem*-difluoroalkenes has the following limitations. For trisubstituted *gem*-difluoroalkenes, such as β,β-difluorostyrene derivatives used by others,^5−12^ the stereoselectivity favors the *Z* product on the basis of steric bias (Scheme 1b). For tetrasubstituted *gem*-difluoroalkenes, such as β,β-difluoroacrylate derivatives used by us, the stereoselectivity favors the *E* product based on chelation control by the directing group (Scheme 1c).^13^ Despite the vast literature in the field,^1−3^ there is a lack of a general stereodivergent strategy of C–F bond functionalization to access *both* (*Z*)- and (*E*)-monofluoroalkenes from the same *gem*-difluoroalkene.

Stereoselective synthesis of multisubstituted alkenes has been a long-standing challenge in organic synthesis.^14^ A stereodivergent catalytic protocol to synthesize both *Z* and *E* isomers from one set of substrates offers a highly attractive solution. For instance, catalytic semihydrogenation of alkynes can lead to *Z* or *E* alkenes;^15a,15b^ however, such a method is not practical and applicable to the preparation of monofluoroalkenes with higher substitution patterns.^15c^ To the best of our knowledge, only Ito and co-workers have reported an indirect Cu(I)-catalyzed stereodivergent hydrodefluorination of *gem*-difluoroalkenes to synthesize disubstituted (*Z*)- and (*E*)-terminal monofluoroalkenes (Scheme 1d).^5d^ The *Z* product was obtained via a borylation/deborylation sequence using B_2_pin_2_, and the *E* product was obtained using hydrosilane. The steric and electronic repulsions were claimed to be key factors in controlling the selectivity of *Z* and *E* products, respectively.

In order to develop a widely applicable stereodivergent C–F bond functionalization, the mode of stereocontrol must be expanded beyond steric/electronic repulsions. We envisioned a catalyst-controlled reaction motif by employing a trisubstituted *gem*-difluoroalkene **1** that contains a directing group (DG), which can potentially take on two different reaction pathways (a and b) depending on the catalytic system, to provide functionalized (*E*)- or (*Z*)-monofluoroalkene **2** (Scheme 2). In path a (directed pathway), **1** undergoes a chelation-assisted C–F bond oxidative addition with a metal catalyst [M^0^] resulting in intermediate **A**. Transmetalation of **A** with an organometallic reagent R-Y at the [M^II^] center leads to intermediate **B**. Final reductive elimination affords product (*E*)-**2** and regenerates catalyst [M^0^]. In path b (nondirected pathway), **1** undergoes migratory insertion with an organometallic species [M^II^-R] resulting in intermediate **C**. Steric effect favors conformation **D** where *syn*-*β-*fluoride elimination takes place to afford the product (*Z*)-**2** and regenerates the [M^II^] catalyst.

**Scheme 2:**
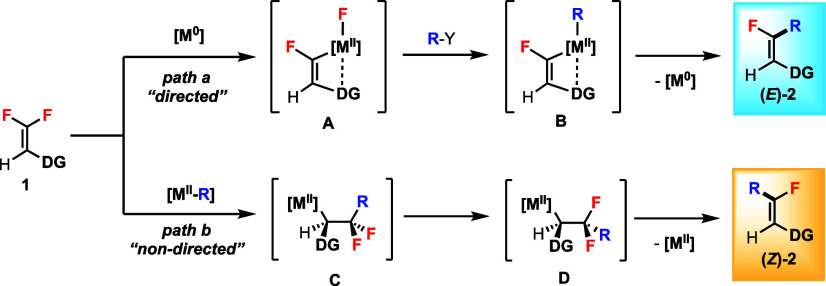
Reaction Design of a Stereodivergent C–F Bond Functionalization via Two Catalytic Pathways

To test our hypothesis, we identified trisubstituted *gem*-difluoroalkene **1a**, which contains an ester group, as a potential directing group, as a candidate for the stereodivergent C–F bond functionalization (Table 1). Compound **1a** is a type of β,β-difluoroacrylate that can be prepared from the corresponding α-diazo ester precursor.^16^ Toste et al. reported a redox-neutral Pd(II)-catalyzed coupling of β,β-difluorostyrenes with boronic acids to synthesize monofluorostilbenes.^11^ When **1a** was subjected to the standard conditions, the monofluoroalkene product **2a** was detected in 52% yield (*Z/E* > 99:1), along with 30% difluoro side product (i.e., conjugate addition product) (entry 1).^17^ We were delighted to see the “nondirected” pathway b (cf. Scheme 2) could provide the *Z* product in excellent diastereoselectivity. The goal was, therefore, to increase the yield of **2a** while decreasing the side product formation by varying the Pd catalysts and ligands.^18^

**Table 1:**
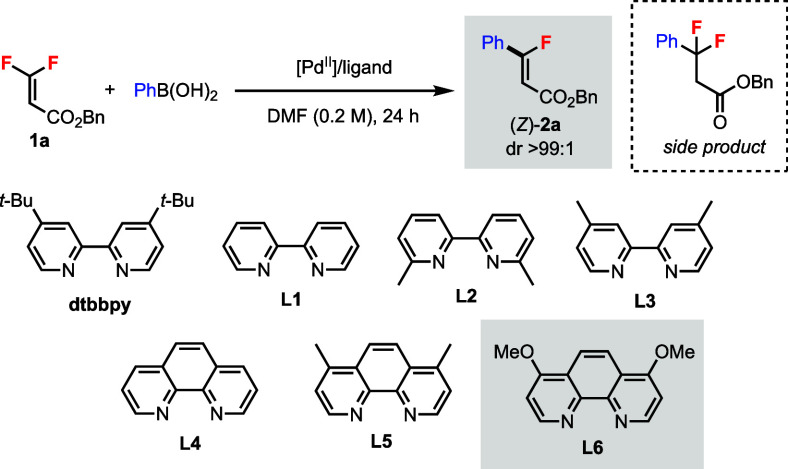
Effects of Pd(II) Catalysts and Ligands for the Stereoselective C–F Bond Functionalization of **1a** to (*Z*)-**2a**.^a^

entry	[Pd^II^]	ligand	yield (%)^b^
1	Pd(TFA)_2_	dtbbpy	52^c^
2	Pd(OAc)_2_	dtbbpy	43
3	PdCl_2_	dtbbpy	8
4	Pd(PPh_3_)_2_Cl_2_	dtbbpy	0
5	Pd(dppf)Cl_2_	dtbbpy	0
6	Pd(MeCN)_2_Cl_2_ (10)	dtbbpy	0
7	Pd(TFA)_2_	**L1**	50
8	Pd(TFA)_2_	**L2**	19
9	Pd(TFA)_2_	**L3**	77
10	Pd(TFA)_2_	**L4**	40
11	Pd(TFA)_2_	**L5**	66
12	Pd(TFA)_2_	**L6**	61^d^
13^e^	Pd(TFA)_2_	**L6**	76
14^e^^,^^f^	Pd(TFA)_2_	**L6**	73
15^e^^,^^f^	Pd(OAc)_2_	**L6**	87

a Unless specified otherwise, reactions were carried out using **1a** (0.1 mmol), PhB(OH)_2_ (0.2 mmol), [Pd^II^] (10 mol %), and ligand (11 mol %) at 50 °C under argon.

b Yield and diastereomeric ratio (dr) were determined by ^19^F NMR analysis using benzotrifluoride as the internal standard.

c Detected 30% of the difluoro side product by ^19^F NMR.

d Only trace difluoro side product was detected by ^19^F NMR.

e **L6** (10 mol %) at 65 °C.

f Reacted with 1.0 equiv of PhB(OH)_2_.

Among the Pd(II) catalysts screened, only Pd(OAc)_2_ gave comparable reactivity as Pd(TFA)_2_ (entries 2–6). 2,2′-Bipyridine ligand **L1** gave a similar yield as dtbbpy (entry 7), and other bipyridine ligands **L2** and **L3** were also compared (entries 8 and 9). 1,10-Phenanthroline ligand **L4** and its analogues **L5** and **L6** were screened (entries 10–12). We found that using ligand **L6** produced the least amount of difluoro side product (entry 12). Increasing the temperature from 50 to 65 °C gave a better yield (entry 13), and 1.0 equiv of boronic acid was effective (entry 14). Finally, we identified the combination of Pd(OAc)_2_/**L6** as the optimal catalytic system for this reaction (entry 15). Other parameters, including solvents, reagents, and stoichiometry were screened with no further improvement.^18^

Under the Pd(II)-catalyzed conditions, various monofluoroalkenes (*Z*)-**2** could be obtained from *gem*-difluoroalkenes **1** in excellent diastereoselectivities (dr > 99:1) (Scheme 3). Arylboronic acids containing different substituents were tolerated to afford products **2a**–**g** in moderate yields. Reaction at the 1.0 mmol scale was also demonstrated (**2a**). Substituent group (R) of the ester moiety could also be varied, including different benzylic (**2h**–**i**), alkyl (**2j**), and aromatic (**2k**–**l**) groups. In all cases, only the *Z* products were formed.

**Scheme 3:**
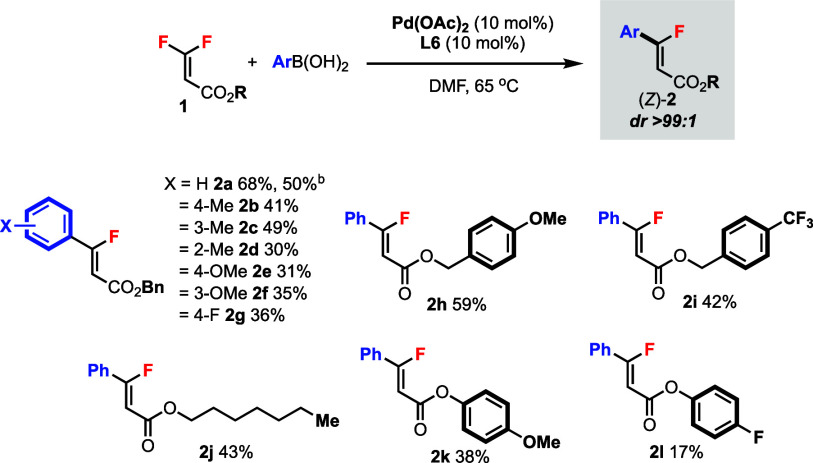
Pd(II)-Catalyzed *Z*-Selective C–F Bond Arylation of *gem*-Difluoroalkenes **1** Unless specified otherwise, reactions were carried out using **1** (0.2 mmol) and arylboronic acid (0.2 mmol) in DMF (1.0 mL) under argon for 1–24 h. Isolated yields. The diastereomeric ratio (dr) was determined by ^19^F NMR analysis. Reacted at 1.0 mmol scale.

Next, we investigated the scope of the “directed” pathway a (cf. Scheme 2) using Pd(0) catalyst and the same *gem*-difluoroalkenes **1** (Scheme 4). Previous work^13c^ has shown that Ph(PPh_3_)_4_-catalyzed Suzuki–Miyaura cross-coupling of tetrasubstituted β,β-difluoroacrylates is highly diastereoselective. To our delight, by applying catalytic Ph(PPh_3_)_4_ to trisubstituted **1a** at 90 °C,^18^ product **3a** could be obtained in 27:1 dr (crude ratio) favoring the *E* diastereomer. The reaction was also performed at a 1.0 mmol scale with similar results. Various arylboronic acids were employed to generate monofluoroalkenes **3b**–**l**. The reaction tolerated substituents at *ortho*, *meta*, and *para* positions of the benzene ring. Electron-rich (**3c**) and electron-poor (**3f**) groups were compatible, although the latter gave a lower dr. Other functional groups, including chloro (**3i**), ester (**3j**), ketone (**3k**), and aldehyde (**3l**), were also tolerated. The diastereoselectivities were generally very high (>20:1 dr). Other boronic acids, such as naphthyl (**3m**), and heteroaryl ones, such as 3-thienyl (**3n**) and 3-furyl (**3o**), provided the products in excellent drs. The ester substituent group of **1** could be varied to include different alkyl (**3p**), benzyl (**3q**,**r**), and aryl (**3s**) groups with good diastereoselectivities. The structure of **3** and (*E*)-alkene geometry were unambiguously confirmed by X-ray crystallography through compound **3q**. Some of the substrates required a lower temperature (80 °C) to ensure high diastereoselectivities. Upon isolation, all products were obtained as a single *E* diastereomer.

**Scheme 4:**
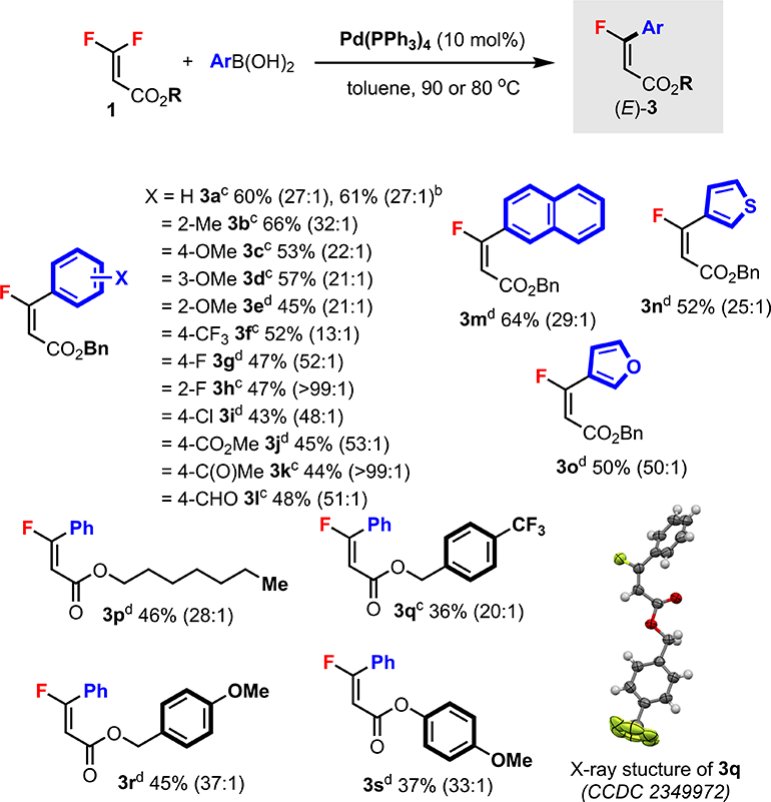
Pd(0)-Catalyzed *E*-Selective C–F Bond Arylation of *gem*-Difluoroalkenes **1** Unless specified otherwise, reactions were carried out using **1** (0.2 mmol) and arylboronic acid (0.3 mmol) in toluene (2.0 mL) under argon for 18 h. Isolated yields. The *E/Z* ratio of the crude mixture determined by ^19^F NMR analysis is shown in the brackets. After isolation, products (*E*)-**3** were obtained in dr > 99:1. Reacted at 1.0 mmol scale. Reacted at 90 °C. Reacted at 80 °C.

Further experiments were conducted to gain insights into the reaction mechanisms (Scheme 5). In the Pd-free reaction using a Grignard reagent, both *Z* and *E* products were obtained in poor selectivity via addition–elimination (Scheme 5a). Thus, the excellent diastereoselectivities in products (*Z*)-**2** and (*E*)-**3** (cf. Schemes 3 and 4) should be controlled by the Pd catalysts through “nondirected” and “directed” pathways, respectively (cf. Scheme 2). Intriguingly, subjecting β,β-difluoroacrylamide **4** to the standard Pd(II)-catalyzed conditions gave no desired product (Scheme 5b). However, under Pd(0)-catalyzed conditions, the monofluoroacrylamide product (*E*)-**5** was obtained in excellent dr (>99:1). The amide functionality could also serve as an effective directing group in this reaction. Following the previous protocol,^13a^ we were able to obtain and characterize the trisubstituted monofluorovinyl Pd(II) complex **Int-1** as a single diastereomer (Scheme 5c). Reacting **Int-1** with boronic acid gave the desired product (*E*)-**3a** in good yield and diastereoselectivity, thus proving the intermediacy of the analogous monofluorovinyl Pd(II) complex in the “directed” pathway (cf. Scheme 2, intermediate **A**).

**Scheme 5:**
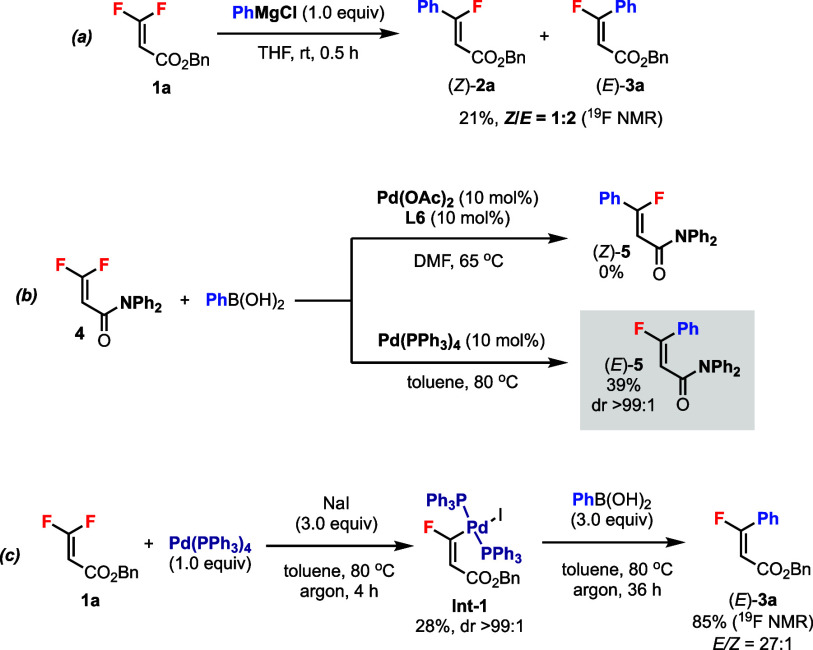
Further Experiments to Gain Mechanistic Insights

In conclusion, we have developed a novel stereodivergent C–F bond functionalization of trisubstituted *gem*-difluoroalkenes **1**. Using the same substrate, both *Z*- and *E*-monofluoroalkene products could be obtained with excellent diastereoselectivities. The stereocontrol relies on two different reaction pathways involving Pd(II)- versus Pd(0)-catalyzed cross-coupling of arylboronic acids. Other types of C–C and *C*-heteroatom bond formation via the stereodivergent reaction design are currently under investigation in our laboratory.

## Data Availability

The data underlying this study are available in the published article and its Supporting Information.

## Supporting Information Available

Single-crystal X-ray diffraction data for compound (*E*)-**3q**, experimental procedures, optimization data, characterization data, and spectral data (PDF)
