# Supplementary material for: Stereodivergent Palladium-Catalyzed C–F Bond Functionalization of gem-Difluoroalkenes
Source: Org Lett. 2024 Jun 27;26(27):5822–6. doi: 10.1021/acs.orglett.4c02112 (PMC11250036; doi:10.1021/acs.orglett.4c02112)

Supporting Information

Stereodivergent Palladium-Catalyzed C-F Bond Functionalization of *gem*-Difluoroalkenes

Yanhui Wang, Gavin Chit Tsui*[a]

[a] Y. Wang, Prof. Gavin Chit Tsui
Department of Chemistry, The Chinese University of Hong Kong, Shatin, New Territories, Hong Kong SAR, China
E-mail: gctsui@cuhk.edu.hk

Experimental Procedures and Spectral Data

<u>Table of Contents:</u>

I. General Experimental……………………………………S2
II. Materials……………………………………S2
III. Instrumentation……………………………………S2
IV. Experimental Procedures……………………………………S4
V. X-Ray Structure of (*E*)-**3q**……………………………………S6
VI. Optimization Studies……………………………………S7
VII. Mechanistic studies……………………………………S11
VIII. Further transformation……………………………………S14
IX. References……………………………………S15
X. Characterization Data……………………………………S16
XI. Spectra……………………………………S27

I. General Experimental.

Unless otherwise noted, C-F bond activation reactions were carried out under argon in a 10 mL glass tube with magnetic stirring. Reactions that require heating were carried out in the oil bath. Analytical thin layer chromatography (TLC) was performed with Merck silica gel 60 F_{254} aluminum plates. Visualization was done under a UV lamp (254 nm) and by immersion in potassium permanganate ($KMnO_4$), followed by heating using a heat gun. Organic solutions were concentrated by rotary evaporation at 23-35 °C. Purification of reaction products were generally done by flash column chromatography with Silicycle 60-230 mesh silica gel.

II. Materials.

Anhydrous NaI, $TMSCF_3$, $TMSCF_2Br$, *n*-Bu_4NB_r, $Pd(PPh_3)_4$, $Pd(OAc)_2$, ligand were purchased from J&K Scientific, Aladdin, Acros, Energy Chemical. Diazo compounds for substates synthesis were prepared according to literature procedure. Other chemicals for substrates preparation were purchased from Acros, J&K Scientific, Aldrich and Dikemann.

III. Instrumentation.

Proton nuclear magnetic resonance spectra (^1H NMR), carbon nuclear magnetic resonance spectra (^{13}C NMR) and fluorine nuclear magnetic resonance spectra (^{19}F NMR) were recorded at 23 °C on Bruker 400 MHz or 500 MHz spectrometer in $CDCl_3$. Chemical shifts of ^1H NMR spectra were reported as parts per million in δ scale using residual solvent signal ($CDCl_3$: 7.26 ppm) or tetramethylsilane (0.00 ppm) as internal standard. Chemical shifts of ^{13}C NMR spectra were reported using residual solvent signal of $CDCl_3$ (77.16 ppm) on the δ scale. Chemical shifts of ^{19}F NMR were reported as parts per million in δ scale using benzotrifluoride (-63.72 ppm) as internal standard. Data are represented as follows: chemical shift (δ ppm), multiplicity (s = singlet, d = doublet, t = triplet, q = quartet, m = multiplet), coupling constant (J, Hz) and integration. High resolution mass spectra (HRMS) were obtained on a Finnigan MAT 95XL GC Mass Spectrometer or a Thermo Scientific Q Exactive Focus Mass Spectrometer or a Bruker SolariX 9.4T FTMS with Q Exactive Focus Orbitrap. X-ray structures were obtained on a Bruker Kappa ApexII Duo Diffractometer or a Bruker D8venture Diffractometer.

Substrates 1 (trisubstituted *gem*-difluoroalkenes)

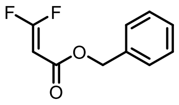

1a

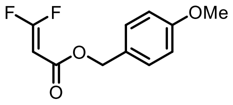

1b

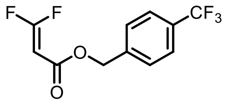

1c

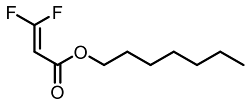

1d

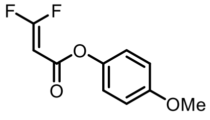

1e

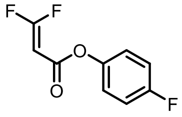

1f

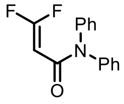

1g

Note: substrates **1** are prepared according to literature procedures.[1, 2,7]

Commercial boronic acid

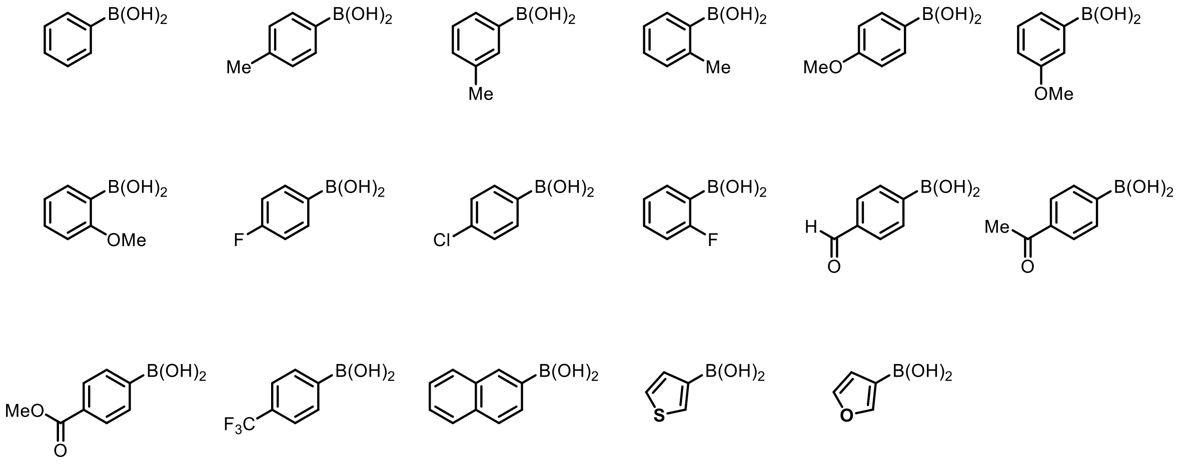

IV. Experimental Procedures.

General procedure (I) for the synthesis of *gem*-difluoroalkenes 1 (using 1a as an example):

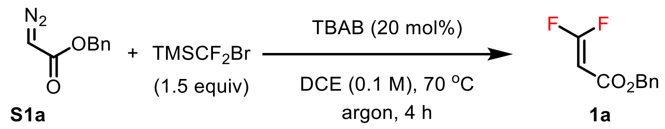

gem-Difluoroalkenes **1** (except **1e**, **1f** and **1g**) were synthesized according to literature procedure.[1] For example, under a argon atmosphere, diazo compounds **s1a** (2.0 mmol, 352.4 mg), TBAB (0.4 mmol, 130 mg) were successively added to a dry 100 mL flask. The reaction flask was degassed three times with argon and dry 1,2-dichloroethane (20 mL) was added using a syringe. Then $TMSCF_2Br$ (3.0 mmol, 610 mg) was added by micro syringe, successively. The reaction tube was then immersed in an oil bath (70 ° C, 4 h). The reaction mixture was then cooled down to room temperature, the reaction mixture was extracted with 200 mL CH_2Cl_2, washed with H_2O (80 mL) then brine (80 mL), dried over $MgSO_4$ and concentrated in vacuo. The residue was purified by flash column chromatography on silica gel to afford pure product **1a** (309.2 mg, 78% yield).

General procedure (II) for the synthesis of *gem*-difluoroalkenes 1 (using 1e as an example):

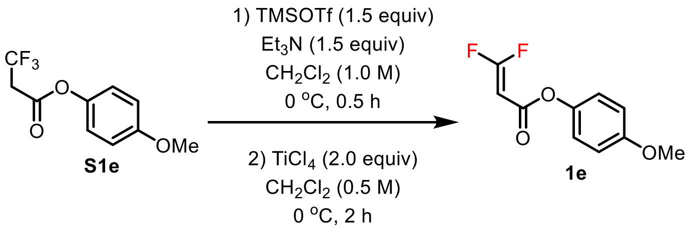

gem-Difluoroalkenes **1e, 1f** and **1g** were synthesized according to literature procedure.[2,7] For example, to a solution of 3,3,3-trifluoropropanoate **S1e** (257.6 mg, 1.1 mmol) in DCM (1 mL) was added triethylamine (0.22 mL, 1.6 mmol), followed by dropwise addition of trimethylsilyl triflate (0.3 mL, 1.6 mmol). The solution was stirred at 0 °C for 0.5 h. Titanium(IV) chloride (2.2 mmol, 0.24 mL) and 1.0 mL CH_2Cl_2 was added. The reaction was stirred at 0 °C for 2 h, and then quenched with water. The reaction mixture was extracted with 30 mL CH_2Cl_2, washed with H_2O (10 mL) then brine (10 mL), dried over $MgSO_4$ and concentrated in vacuo. The residue was purified by flash column chromatography on silica gel to afford *gem*-difluoroalkene **1e** as a colorless oil (126 mg, 54% yield).

General procedure (III) for the stereoselective synthesis of (*Z*)-monofluoroalkenes :

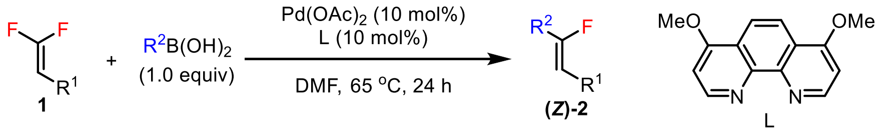

To an oven-dried glass tube equipped with a stir bar, $Pd(OAc)_2$ (4.5 mg, 0.02 mmol), 4,7-dimethoxy-1,10-phenanthroline (4.8 mg, 0.02 mmol) was added. The solids were suspended in 0.5 mL of DMF and stirred for 30 minutes. Difluoroalkene substrate **1** (0.2 mmol) was added as a solution in 0.5 mL of DMF. Aryl boronic acid (0.2 mmol) was added as a solid. The glass tube was capped and was placed in a 65 °C oil bath for 24 h. After cooling to room temperature, the reaction mixture was extracted with CH_2Cl_2 (3 × 10 mL). The combined organic layers were washed with H_2O (2 × 10 mL), then brine (2 × 10 mL), dried over $MgSO_4$ and concentrated in vacuo. The residue was purified by flash column chromatography on silica gel to afford products (*Z*)-**2**.

General procedure (IV) for the stereoselective synthesis of (*E*)-monofluoroalkenes :

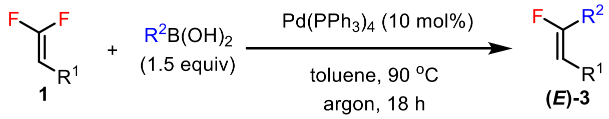

To an oven-dried glass tube equipped with a stir bar was added $Pd(PPh_3)_4$ (23.1 mg, 0.02 mmol) and boronic acid (0.3 mmol). The tube was sealed with a septum, evacuated and refilled with argon three times. Then a solution of **1** (0.2 mmol) in 2.0 mL toluene was added under argon through syringe. The resulting mixture was heated at 90 °C with stirring in an oil bath

for 18 h. After cooling to room temperature, the reaction mixture was extracted with CH_2Cl_2 (3 × 10 mL). The combined organic layers were washed with H_2O (2 × 10 mL), then brine (2 × 10 mL), dried over $MgSO_4$ and concentrated in vacuo. The residue was purified by flash column chromatography on silica gel to afford products (*E*)-**3**.

Gram-scale synthesis of (*Z*)-2a:

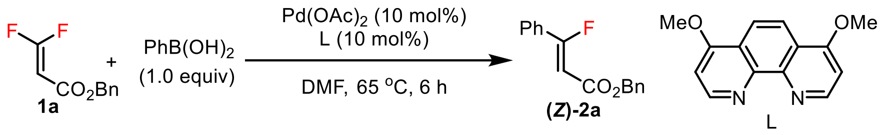

To an oven-dried glass tube equipped with a stir bar, $Pd(OAc)_2$ (22.4 mg, 0.1 mmol), 4,7-dimethoxy-1,10-phenanthroline (24.0 mg, 0.1 mmol) was added. The solids were suspended in 2.5 mL of DMF and stirred for 30 minutes. Difluoroalkene substrate **1a** (198.2 mg, 1.0 mmol) was added as a solution in 2.5 mL of DMF. Phenyl boronic acid (121.9 mg, 1.0 mmol) was added as a solid. The glass tube was capped and was placed in a 65 °C oil bath for 6 h. After cooling to room temperature, the reaction mixture was extracted with CH_2Cl_2 (3 × 20 mL). The combined organic layers were washed with H_2O (2 × 20 mL), then brine (2 × 20 mL), dried over $MgSO_4$ and concentrated in vacuo. The residue was purified by flash column chromatography on silica gel (ethyl acetate: hexane = 1 : 100) to afford products (*Z*)-**2a** as colorless oil (129.4 mg, 50%; *E*/*Z* >99:1, ^{19}F NMR).

Gram-scale synthesis of (*E*)-3a:

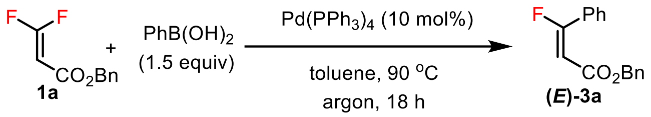

To an oven-dried glass tube equipped with a stir bar was added $Pd(PPh_3)_4$ (115.6 mg, 0.1 mmol) and phenyl boronic acid (183.0 mg, 1.5 mmol). The tube was sealed with a septum, evacuated and refilled with argon three times. Then a solution of **1a** (198.2 mg, 1.0 mmol) in 10 mL toluene was added under argon through syringe. The resulting mixture was heated at 90 °C with stirring in an oil bath for 18 h. After cooling to room temperature, the reaction mixture was extracted with CH_2Cl_2 (3 × 20 mL). The combined organic layers were washed with H_2O (2 × 20 mL), then brine (2 × 20 mL), dried over $MgSO_4$ and concentrated in vacuo. The residue was purified by flash column chromatography on silica gel (CH_2Cl_2 : hexane = 1 : 20) to afford products (*E*)-**3a** as colorless oil (155.8 mg, 61%; *E*/*Z* >99:1, ^{19}F NMR).

V. X-Ray Structure of (*E*)-3q

Crystals of (*E*)-**3q** were obtained by slow diffusion from the solution in $CHCl_3$ layered *n*-hexane. The crystal was kept at 296 K during data collection. Crystallographic data for (*E*)-**3q** has been deposited with the Cambridge Crystallographic Data Centre (CCDC) under deposition number 2349972.

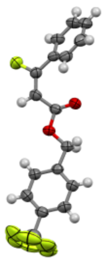

Molecular structure of (*E*)-**3q** with thermal ellipsoids at the 50% probability level

Crystal data and structure refinement for (*E*)-**3q**.

Identification code	CCDC 2349972	
Empirical formula	$C_{17}H_{12}F_4O_2$	
Formula weight	324.27	
Temperature	296(2) K	
Wavelength	0.71073 Å	
Crystal system	Triclinic	
Space group	P-1	
Unit cell dimensions	a = 5.8940(11) Å	α= 86.935(5)°.
	b = 8.1787(16) Å	β= 87.009(5)°.
	c = 15.643(3) Å	γ = 86.270(5)°.
Volume	750.5(2) Å^3	
Z	2	
Density (calculated)	1.435 Mg/m^3	
Absorption coefficient	0.126 mm^{-1}	
F(000)	332	
Crystal size	0.500 x 0.300 x 0.300 mm^3	
Theta range for data collection	2.499 to 25.249°.	
Index ranges	-7<=h<=7, -9<=k<=9, -18<=l<=18	
Reflections collected	16637	
Independent reflections	2698 [R(int) = 0.0262]	
Completeness to theta = 25.242°	99.5 %	
Absorption correction	multi-scan	
Max. and min. transmission	0.7456 and 0.6898	
Refinement method	Full-matrix least-squares on F^2	
Data / restraints / parameters	2698 / 36 / 236	
Goodness-of-fit on F^2	1.058	
Final R indices [I>2sigma(I)]	R1 = 0.0480, wR2 = 0.1226	
R indices (all data)	R1 = 0.0653, wR2 = 0.1416	
Extinction coefficient	n/a	
Largest diff. peak and hole	0.165 and -0.153 e.Å^{-3}	

VI. Optimization studies.

Table S1. Screening of Pd(II) catalysts and ligands for the formation of (*Z*)-2a.[a]

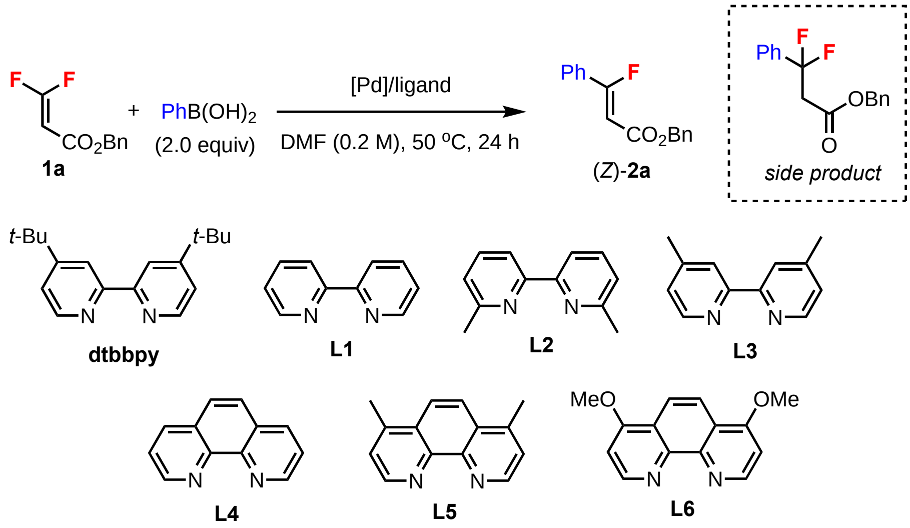

entry	[Pd] (mol%)	ligand	yield (%)[b]	*Z/E*[b]
1	$Pd(TFA)_2$ (10)	dtbbpy (11)	52[d]	>99:1
2	$Pd(OAc)_2$ (10)	dtbbpy (11)	43	>99:1
3	$PdCl_2$(10)	dtbbpy (11)	8	>99:1
4	$Pd(PPh_3)_2Cl_2$ (10)	dtbbpy (11)	0	-
5	$Pd(dppf)Cl_2$ (10)	dtbbpy (11)	0	-
6	$Pd(MeCN)_2Cl_2$ (10)	dtbbpy (11)	0	-
7	$Pd(TFA)_2$ (10)	L1 (11)	50	>99:1
8	$Pd(TFA)_2$ (10)	L2 (11)	19	>99:1
9	$Pd(TFA)_2$ (10)	L3 (11)	77	>99:1
10	$Pd(TFA)_2$ (10)	L4 (11)	40	>99:1
11	$Pd(TFA)_2$ (10)	L5 (11)	66	>99:1
12	$Pd(TFA)_2$ (10)	L6 (11)	61[e]	>99:1
13	$Pd(TFA)_2$ (10)	L6 (20)	13	>99:1
14	$Pd(TFA)_2$ (10)	L6 (15)	60	>99:1
**15**	**$Pd(TFA)_2$ (10)**	**L6 (10)**	**59 (43)[c]**	**>99:1**
16	$Pd(TFA)_2$ (10)	L6 (7)	59	>99:1
17	$Pd(TFA)_2$ (10)	L6 (5)	52	>99:1

[a]Unless specified otherwise, reactions were carried out using **1a** (0.1 mmol) under argon. [b]Determined by ^{19}F NMR analysis using benzotrifluoride as the internal standard. [c]Isolated yield. [d]Detected 30% of the difluoro side product by ^{19}F NMR. [e]Only trace amounts of the difluoro side product was detected by ^{19}F NMR.

Table S2. Screening of temperature, solvents and other parameters for the formation of (*Z*)-2a.[a]

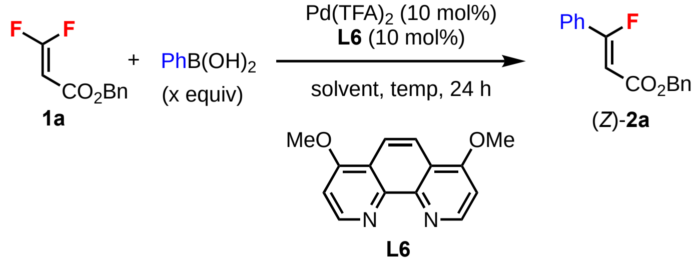

entry	x	temp (°C)	solvent (M)	yield (%)[b]	*Z/E*[b]
1	2.0	rt	DMF (0.2)	5	>99:1
2	2.0	65	DMF (0.2)	76	>99:1
3	2.0	75	DMF (0.2)	43	>99:1
4	2.0	100	DMF (0.2)	38	>99:1
5	1.5	65	DMF (0.2)	73	>99:1
6	1.2	65	DMF (0.2)	64	>99:1
7	1.1	65	DMF (0.2)	63	>99:1
**8**	**1.0**	**65**	**DMF (0.2)**	**73**	**>99:1**
9	1.0	65	DMSO (0.2)	4	>99:1
10	1.0	65	toluene (0.2)	12	>99:1
11	1.0	65	1,4-dioxane (0.2)	23	>99:1
12	1.0	65	DCE (0.2)	10	>99:1
13	1.0	65	DMF (0.3)	51	>99:1
14	1.0	65	DMF (0.1)	59	>99:1

[a]Unless specified otherwise, reactions were carried out using **1a** (0.1 mmol) under argon. [b]Determined by ^{19}F NMR analysis using benzotrifluoride as the internal standard.

Table S3. Screening of reagents and other parameters for the formation of (*Z*)-2a.[a]

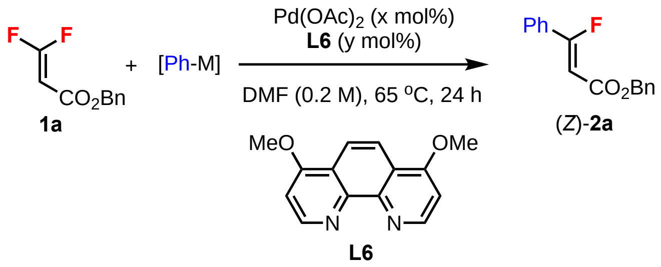

entry	x	y	[Ph-M] (equiv)	yield (%)[b]	*Z/E*[b]
**1**	**10**	**10**	**$PhB(OH)_2$ (1.0)**	**87 (80)**[c]	**>99:1**
2	10	10	$PhBF_3K$ (1.0)	0	-
3	10	10	PhBPin (1.0)	48	>99:1
4	10	10	$(PhBO)_3$ (0.33)	63	>99:1
5	10	10	$PhSi(OEt)_3$ (1.0)	0	-
6	10	10	$PhMe_2SiCl$ (1.0)	0	-
7	10	10	Ph_2Cl_2Si (1.0)	0	-
8	10	10	$PhMe_2$vinylSi (1.0)	0	-
9	10	10	Ph_3SiH (1.0)	0	-
10	10	10	Ph(*n*-Bu)$_3$Sn (1.0)	0	-
11	5	5	$PhB(OH)_2$ (1.0)	43	>99:1
12	2	2	$PhB(OH)_2$ (1.0)	19	>99:1
13	10	0	$PhB(OH)_2$ (1.0)	0	-
14	0	10	$PhB(OH)_2$ (1.0)	0	-

[a]Unless specified otherwise, reactions were carried out using **1a** (0.1 mmol) under argon. [b]Determined by ^{19}F NMR analysis using benzotrifluoride as the internal standard. [c]Isolated yield

Table S4. Optimization studies for the formation of (*E*)-3a.[a]

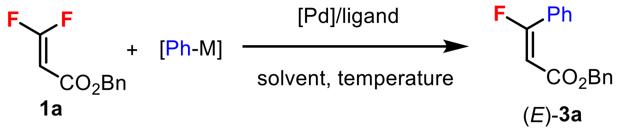

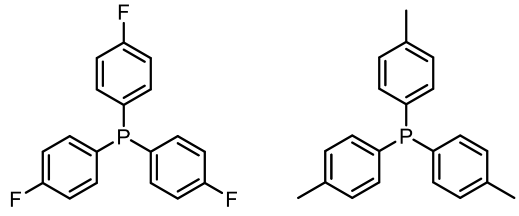

entry	[Pd] (mol%)	ligand (mol%)	[Ph-M] (equiv)	solvent (M)	temp (°C)/ time (h)	yield (%)[b]
1	$Pd(PPh_3)_4$ (10)	-	$PhB(OH)_2$ (3.0)	toluene (0.1)	80/18	43
2	$Pd(PPh_3)_4$ (10)	-	$PhBF_3K$ (3.0)	toluene (0.1)	80/18	10
3	$Pd(PPh_3)_4$ (10)	-	PhBPin (3.0)	toluene (0.1)	80/18	0
4	$Pd(PPh_3)_4$ (10)	-	$(PhBO)_3$ (1.0)	toluene (0.1)	80/18	90
5	$Pd(PPh_3)_4$ (10)	-	$PhSi(OEt)_3$ (3.0)	toluene (0.1)	80/18	3
6	$Pd(PPh_3)_4$ (10)	-	$PhB(OH)_2$ (3.0)	toluene (0.1)	90/18	62
7	$Pd(PPh_3)_4$ (10)	-	$PhB(OH)_2$ (3.0)	toluene (0.1)	100/18	79
8	$Pd(PPh_3)_4$ (10)	-	$PhB(OH)_2$ (3.0)	toluene (0.2)	90/18	56
9	$Pd(PPh_3)_4$ (10)	-	$PhB(OH)_2$ (3.0)	toluene (0.5)	90/18	42
10	$Pd(PPh_3)_4$ (5)	-	$PhB(OH)_2$ (3.0)	toluene (0.1)	90/18	40
11	$Pd(PPh_3)_4$ (10)	-	$PhB(OH)_2$ (3.0)	toluene (0.1)	90/48	52
12	$Pd(PPh_3)_4$ (10)	-	$PhB(OH)_2$ (2.0)	toluene (0.1)	90/18	59
**13**	**$Pd(PPh_3)_4$ (10)**	**-**	**$PhB(OH)_2$ (1.5)**	**toluene (0.1)**	**90/18**	**57**[c]
14	$Pd(PPh_3)_4$ (10)	-	$PhB(OH)_2$ (1.2)	toluene (0.1)	90/18	53
15	$Pd(PPh_3)_4$ (10)	-	$PhB(OH)_2$ (1.5)	toluene (0.2)	90/18	40
16	$Pd(PPh_3)_4$ (10)	PPh_3 (20)	$PhB(OH)_2$ (1.5)	toluene (0.1)	90/18	32
17	$Pd_2(dba)_3$ (5)	**L1** (20)	$PhB(OH)_2$ (1.5)	toluene (0.1)	80/18	<5
18	$Pd_2(dba)_3$ (5)	**L2** (20)	$PhB(OH)_2$ (1.5)	toluene (0.1)	80/18	0
19	$Pd(OAc)_2$ (10)	PPh_3 (20)	$PhB(OH)_2$ (1.5)	toluene (0.1)	80/18	44
20	$Pd(OAc)_2$ (10)	PPh_3 (40)	$PhB(OH)_2$ (1.5)	toluene (0.1)	80/18	46
21	-	-	$PhB(OH)_2$ (1.5)	toluene (0.1)	90/18	0

[a]Unless specified otherwise, reactions were carried out using **1a** (0.1 mmol) under argon. [b]Determined by ^{19}F NMR analysis using benzotrifluoride as the internal standard. [c]dr = 28:1 by ^{19}F NMR analysis of the crude mixture.

VII. Mechanistic studies.

Pd-free arylation of 1a.

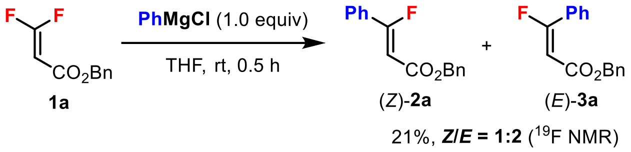

To an oven-dried glass tube equipped with a stir bar was added **1a** (19.8 mg, 0.1 mmol) in 0.6 mL THF. The tube was sealed with a septum, evacuated and refilled with argon three times. PhMgCl (0.1 mmol, 0.1 L, 1.0 M in THF) was added through microsyringe dropwise under argon, the resulting mixture was kept stirring for 0.5 h. The crude mixture was analyzed by ^{19}F NMR using benzotrifluoride as internal standard (*E*)-**2a** (14% ^{19}F NMR yield) and (*Z*)-**3a** (7% ^{19}F NMR yield).

Preparation of monofluorovinylpalladium(II) iodide complex Int-1 and stoichiometric reaction between Int-1 and PhB(OH)$_2$:

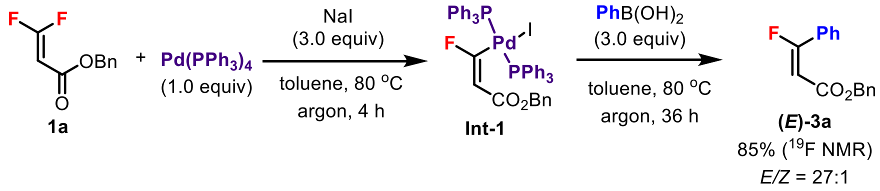

The complex **Int-1** was prepared followed by a reported procedure.[3] To an oven-dried 10 mL glass tube equipped with a stir bar was added Pd(PPh$_3$)$_4$ (115.6 mg, 0.1 mmol) and NaI (45 mg, 0.3 mmol). The tube was sealed with a septum, evacuated and refilled with argon three times. Then a solution of **1a** (19.8 mg, 0.1 mmol) in 1.0 mL toluene was added through syringe. The resulting mixture was heated at 80 °C with stirring under argon for 4 h. The mixture was cooled to room temperature, then filtered with cotton to remove solid residues, which were rinsed with 1 mL toluene resulting in a clear red filtrate solution (~2.5 mL). To this solution was added 25 mL dry hexane, an orange solid precipitated out upon ultrasonication. The solid was then collected by filtration as a crude product. To the crude product was added 4 mL dry hexane followed by ultrasonication, a mixture of yellow and red solids was visible. To this mixture was carefully added CH_2Cl_2 dropwise until most yellow solids dissolved (red solids remained). The red solids were filtered off and the filtrate (clear yellow solution) was collected, and the solvent was removed *in vauo*. The residue was purified by flash column chromatography on silica gel (ethyl acetate: hexane = 1 : 50) to afford products **Int-1** as yellow solid (26.7 mg, 28%; d.r. >99:1, ^{19}F NMR). **^{1}H NMR** (500 MHz, CDCl$_3$): δ (ppm) 7.75 (q, J = 6.3 Hz, 12H), 7.40 (t, J = 7.4 Hz, 6H), 7.42 – 7.26 (m, 17H), 4.97 (s, 2H), 4.54 (d, J = 25.9 Hz, 1H). **^{13}C NMR** (126 MHz, CDCl$_3$): δ (ppm) 213.3 (d, J = 363.0 Hz), 168.5 (d, J = 32.1 Hz), 109.3 (d, J = 16.2 Hz), 64.8, remaining signals not resolved. **^{19}F NMR** (471 MHz, CDCl$_3$): δ (ppm) -23.72 (dt, J = 27.1, 14.6 Hz, 1F). **^{31}P NMR** (202 MHz, CDCl$_3$): δ (ppm) 20.70 (d, J = 12.6 Hz). **HRMS** (ESI) m/z: [M+Na]$^+$ Calcd for $C_{46}H_{38}P_2O_2IFPdNa$ 959.0319; Found 959.0310.

To an oven-dried NMR tube was added **Int-1 (**26.7 mg, 0.03 mmol) and phenylboronic acid (11 mg, 0.09 mmol), the tube was sealed with a septum, evacuated, and refilled with argon three times. 1.0 mL toluene was then added through syringe under argon. The resulting mixture was heated at 80 °C for 36 h, then cooled to room temperature. **(*E*)-3a** was obtained in 85% yield (*E/Z* = 27:1) by ^{19}F NMR analysis using benzotrifluoride (3.6 µL, 0.03 mmol) as internal standard.

Isomerization of product.

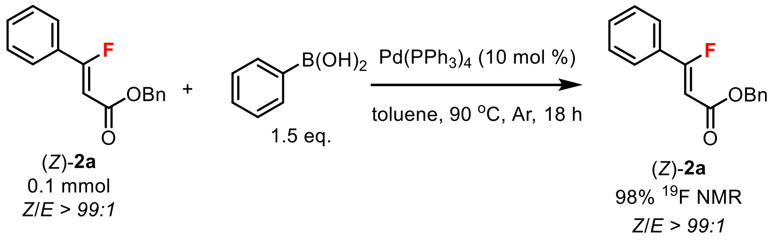

To an oven-dried 10 mL glass tube equipped with a stir bar was added PhB(OH)$_2$ (18.3 mg, 0.15 mmol) and Pd(PPh$_3$)$_4$ (11.5 mg, 0.01 mmol), the tube was sealed with a septum, evacuated and refilled with argon three times. (*Z*)-**2a** (25.6 mg, 0.1 mmol, *Z*/*E* >99:1) in 1.0 mL toluene was added under argon. The tube was sealed and heated at 90 °C in the oil bath for 18 h. The reaction was monitored by ^{19}F NMR (471MHz, *Z*/*E* >99:1).

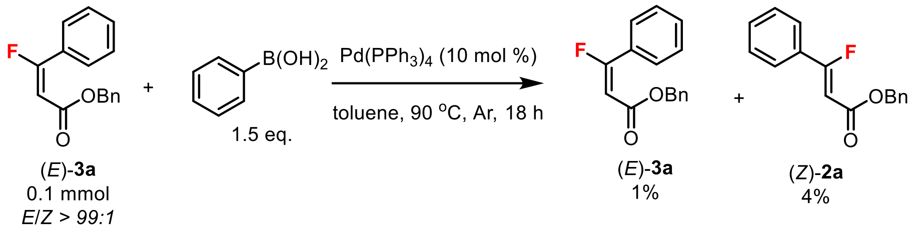

To an oven-dried 10 mL glass tube equipped with a stir bar was added $PhB(OH)_2$ (18.3 mg, 0.15 mmol) and $Pd(PPh_3)_4$ (11.5 mg, 0.01 mmol), the tube was sealed with a septum, evacuated and refilled with argon three times. (*E*)-**3a** (25.6 mg, 0.1 mmol, *E*/*Z* >99:1) in 1.0 mL toluene was added under argon. The tube was sealed and heated at 90 °C in the oil bath for 18 h. The reaction was monitored by ^{19}F NMR (471MHz), 1% (*E*)-**3a** and 4% (*Z*)-**2a** was detected.

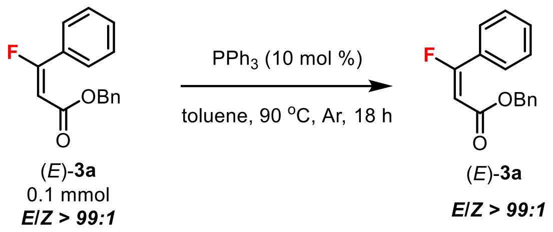

To an oven-dried 10 mL glass tube equipped with a stir bar was added PPh_3 (2.6 mg, 0.01 mmol), the tube was sealed with a septum, evacuated and refilled with argon three times. (*E*)-**3a** (25.6 mg, 0.1 mmol, *E*/*Z* >99:1) in 1.0 mL toluene was added under argon. The tube was sealed and heated at 90 °C in the oil bath for 18 h. The reaction was monitored by ^{19}F NMR (471MHz, *E*/*Z* >99:1).

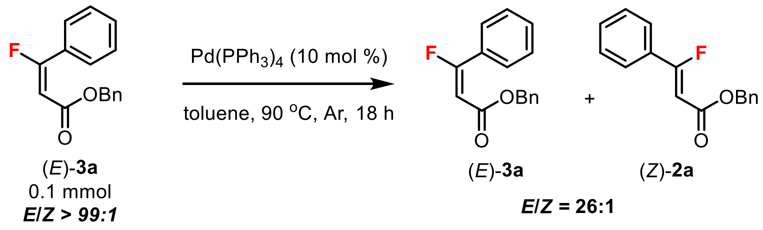

To an oven-dried 10 mL glass tube equipped with a stir bar was added $Pd(PPh_3)_4$ (11.5 mg, 0.01 mmol), the tube was sealed with a septum, evacuated and refilled with argon three times. (*E*)-**3a** (25.6 mg, 0.1 mmol, *E*/*Z* >99:1) in 1.0 mL toluene was added under argon. The tube was sealed and heated at 90 °C in the oil bath for 18 h. The reaction was monitored by ^{19}F NMR (471MHz, *E*/*Z* = 26:1).[4]

Reactivity of dibromo and dichloro substrate.

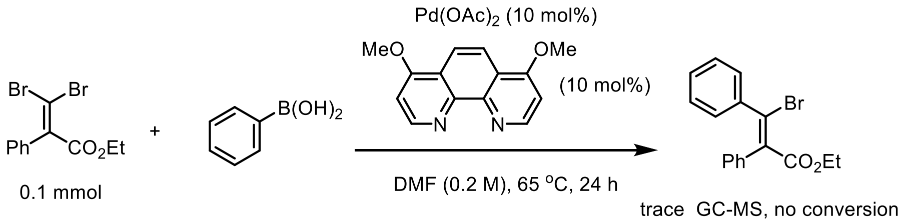

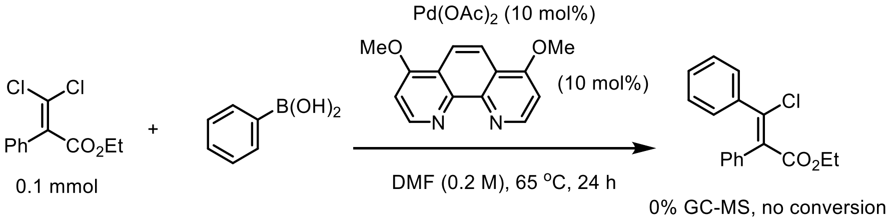

To an oven-dried glass tube equipped with a stir bar, $Pd(OAc)_2$ (2.2 mg, 0.01 mmol), 4,7-dimethoxy-1,10-phenanthroline (2.4 mg, 0.01 mmol) was added. The solids were suspended in 0.25 mL of DMF and stirred for 30 minutes. Dibromoalkene or dichloroalkene (0.1 mmol) was added as a solution in 0.25 mL of DMF. Phenyl boronic acid (12.2mg, 0.1 mmol) was added as

a solid. The glass tube was capped and was placed in a 65 ºC oil bath for 24 h. After cooling to room temperature, the reaction mixture was monitored by GC-MS. Only trace amount or no desired product can be detected due to no conversion.

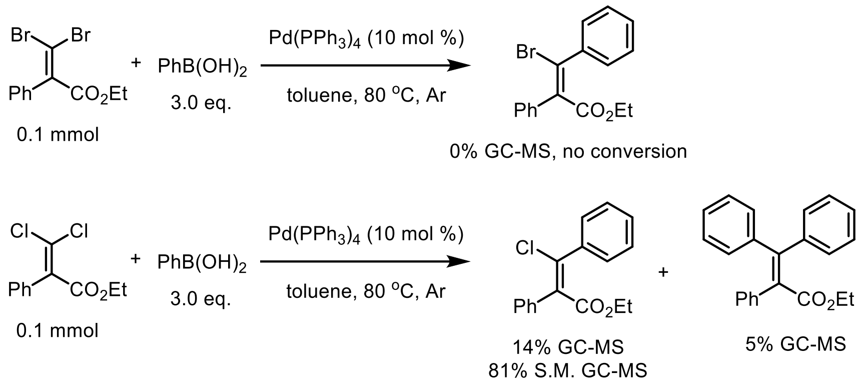

To an oven-dried glass tube equipped with a stir bar was added Pd(PPh$_3$)$_4$ (11.5 mg, 0.01 mmol) and phenyl boronic acid (36.6 mg, 0.3 mmol). The tube was sealed with a septum, evacuated and refilled with argon three times. Then a solution of dibromoalkene or dichloroalkene (0.1 mmol) in 1.0 mL toluene was added under argon through syringe. The resulting mixture was heated at 80 °C with stirring in an oil bath for 18 h. After cooling to room temperature, the reaction mixture was monitored by GC-MS. Only trace amount or no desired product can be detected due to no conversion.

VIII. Further transformations.

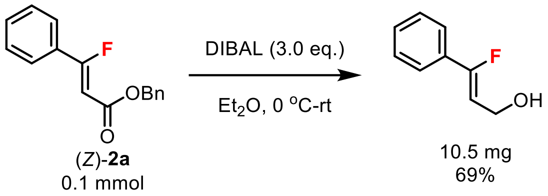

Followed a literature procedure[5] : to a solution of (*Z*)-**2a** (25.6 mg, 0.1 mmol, *Z*/*E* >99:1) in Et_2O (0.5 mL) at 0 °C was added DIBAL (1.0 M in hexane, 0.3 mL, 0.3 mmol) over 5 min and the mixture was stirred at this temperature for 20 min, and then at room temperature for 5 h. The crude mixture was cooled to 0 °C and quenched carefully with 1.0 M aqueous HCl solution until the pH of the mixture was 5-6. The reaction mixture was extracted with Et_2O (3 ×10 mL). The combined organic layers were washed with H_2O (2 ×10 mL), then brine (2 ×10 mL), dried over MgSO4 and concentrated in vacuo. The residue was purified by flash column chromatography on silica gel (EA : hexane = 1 : 10) and product was obtained as a colorless oil (10.5 mg, 69% yield, dr >99:1), Rf = 0.21 (EA : hexane = 1:2). **^{1}H NMR** (500 MHz, $CDCl_3$): δ (ppm) 7.54 (dd, *J* = 7.8, 1.9 Hz, 2H), 7.40 – 7.35 (m, 3H), 5.67 (dt, *J* = 36.5, 7.1 Hz, 1H), 4.45 (dd, *J* = 7.1, 2.1 Hz, 2H), 1.60 (s, 1H). **^{13}C NMR** (126 MHz, $CDCl_3$): δ (ppm) 158.2 (d, *J* = 251.3 Hz), 131.8 (d, *J* = 28.6 Hz), 129.5, 128.7 (d, *J* = 2.1 Hz), 124.5 (d, *J* = 7.2 Hz), 104.9 (d, *J* = 15.1 Hz), 56.3 (d, *J* = 7.7 Hz). **^{19}F NMR** (471 MHz, $CDCl_3$): δ (ppm) -117.23 (d, *J* = 36.8 Hz, 1F). The spectra are in full accordance with the literature report.[6]

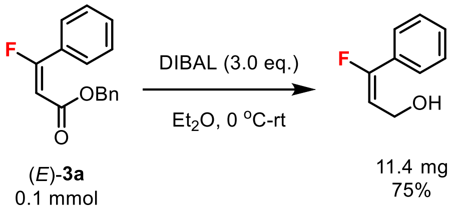

Followed a literature procedure[1] : to a solution of (*E*)-**3a** (25.6 mg, 0.1 mmol, E/Z >99:1) in Et_2O (0.5 mL) at 0 °C was added DIBAL (1.0 M in hexane, 0.3 mL, 0.3 mmol) over 5 min and the mixture was stirred at this temperature for 20 min, and then at room temperature for 5 h. The crude mixture was cooled to 0 °C and quenched carefully with 1.0 M aqueous HCl solution until the pH of the mixture was 5-6. The reaction mixture was extracted with Et_2O (3 ×10 mL). The combined organic layers were washed with H_2O (2 ×10 mL), then brine (2 ×10 mL), dried over MgSO4 and concentrated in vacuo. The residue was purified by flash column chromatography on silica gel (EA : hexane = 1 : 10) and product was obtained as a colorless oil (11.4 mg, 75% yield, dr >99:1), Rf = 0.22 (EA : hexane = 1:2). **^{1}H NMR** (500 MHz, $CDCl_3$): δ (ppm) 7.50 – 7.42 (m, 5H), 5.66 (dt, *J* = 20.0, 7.9 Hz, 1H), 4.29 (d, *J* = 7.9 Hz, 2H). **^{13}C NMR** (126 MHz, $CDCl_3$): δ (ppm) 160.4 (d, *J* = 249.5 Hz), 131.1 (d, *J* = 29.2 Hz), 129.9, 128.7, 128.0 (d, *J* = 4.9 Hz), 107.1 (d, *J* = 24.4 Hz), 58.1 (d, *J* = 12.2 Hz). **^{19}F NMR** (471 MHz, $CDCl_3$): δ (ppm) -97.24 (d, *J* = 20.0 Hz, 1F). The spectra are in full accordance with the literature report.[6]

IX. References.

1. Zhang, Z.; Yu, W.; Wu, C.; Wang, C.; Zhang, Y.; Wang, J. Reaction of Diazo Compounds with Difluorocarbene: An Efficient Approach towards 1,1-Difluoroolefins. *Angew. Chem., Int. Ed.* **2016**, *55*, 273-277.
2. (a) McAlpine, I.; Tran-Dube, M.; Wang, F.; Scales, S.; Matthews,´ J.; Collins, M. R.; Nair, S. K.; Nguyen, M.; Bian, J.; Alsina, L. M.; Sun, J.; Zhong, J.; Warmus, J. S.; O'Neill, B. T. Synthesis of Small 3-Fluoroand 3,3-Difluoropyrrolidines Using Azomethine Ylide Chemistry. *J. Org. Chem.* **2015**, *80*, 7266-7274. (b)Shimada, T.; Konno, T.; Ishihara, T. A New Access to 3-Halo-3,3-difluoropropanoic Acid Derivatives via Fluorine-Halogen Exchange Reaction of Silyl Enolates of 3,3,3-Trifluoropropanoic Acid Derivatives. *Chem. Lett.* **2007**, *36*, 636-637.
3. Ma, Q.; Wang, Y.; Tsui, G. C. Stereoselective palladium-catalyzed C-F Bond alkynylation of tetrasubstituted *gem*-difluoroalkenes. *Angew. Chem., Int. Ed.* **2020**, *59*, 11293-11297.
4. Canovese, L.; Santo, C.; Visentin, F. Palladium(0)-Catalyzed Cis-Trans Alkene Isomerizations. *Organometallics* **2008**, *27*, 3577-3581.
5. Li, M.; Wang, Y.; Tsui, G. C. Palladium-Catalyzed Stereoselective C-F Bond Vinylation and Allylation of Tetrasubstituted gem-Difluoroalkenes via Stille Coupling: Synthesis of Monofluorinated 1,3- and 1,4-Dienes. *Org. Lett.* **2021**, *23*, 8072-8076.
6. Gillet, J. P.; Sauvetre, R.; Normant, J. F. Carboxylation of 2, 2-Difluorovinyllithium: A New General Synthesis of *α*, *β*-Unsaturated *β*-Fluoroalcohols,-ketones, and-acids. *Synthesis* **1982**, *1982*, 297-301.
7. Wang, Y.; Tang, Y.; Zong, Y.; Tsui, G. C. Highly Selective C-F Bond Functionalization of Tetrasubstituted *gem*-Difluoroalkenes and Trisubstituted Monofluoroalkenes Using Grignard Reagents. *Org. Lett.* **2022**, *24*, 4087-4092.
8. Thornbury, R. T.; Toste, F. D. Palladium-Catalyzed Defluorinative Coupling of 1-Aryl-2, 2-Difluoroalkenes and Boronic Acids: Stereoselective Synthesis of Monofluorostilbenes. *Angew. Chem., Int. Ed.* **2016**, *55*, 11629-11632.
9. Lecea, M.; Grassin, A.; Ferreiro-Mederos, L.; Choppin, S.; Urbano, A.; Carreňo, M. C.; Colobert, F. One-Step Stereoselective Synthesis of Trisubstituted Monofluoroalkenes from 3,3,3-Trifluoropropionates. *Eur. J. Org. Chem.* **2013**, 4486.

X. Characterization Data.

Characterization data of products

benzyl (*Z*)-3-fluoro-3-phenylacrylate (2a)

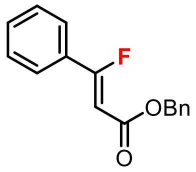

Following the general procedure (III), reaction was run using *gem*-difluoroalkene **1a** (39.6 mg, 0.2 mmol), phenylboronic acid (24.4 mg, 0.2 mmol), $Pd(OAc)_2$ (4.5 mg, 0.02 mmol), 4,7-dimethoxy-1,10-phenanthroline (4.8 mg, 0.02 mmol) in 1.0 mL DMF in a 65 °C oil bath for 24 h. The product was purified by flash column chromatography on silica gel (ethyl acetate : hexane = 1 : 100) and obtained as a colorless oil (34.8 mg, 68% yield), R_f = 0.25 (ethyl acetate : hexane = 1 : 10). **^{1}H NMR** (500 MHz, $CDCl_3$): δ (ppm) 7.66 – 7.65 (m, 2H), 7.50 – 7.33 (m, 8H), 5.96 (d, *J* = 33.1 Hz, 1H), 5.25 (s, 2H). **^{13}C NMR** (126 MHz, $CDCl_3$): δ (ppm) 166.9 (d, *J* = 278.5 Hz), 164.0, 136.1, 131.8, 130.7 (d, *J* = 26.0 Hz), 129.0 (d, *J* = 2.1 Hz), 128.7, 128.4, 128.4, 125.8 (d, *J* = 7.9 Hz), 97.0 (d, *J* = 6.7 Hz), 66.4. **^{19}F NMR** (471 MHz, $CDCl_3$): δ (ppm) -97.57 (d, *J* = 33.1 Hz, 1F). **HRMS** (ESI) m/z: $[M+Na]^+$ Calcd for $C_{16}H_{13}FO_2Na$ 279.0792; Found 279.0790.[8-9]

benzyl (*Z*)-3-fluoro-3-(p-tolyl)acrylate (2b)

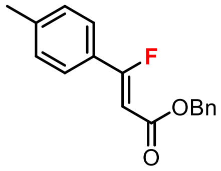

Following the general procedure (III), reaction was run using *gem*-difluoroalkene **1a** (39.6 mg, 0.2 mmol), (*p*-methylphenyl) boronic acid (27.2 mg, 0.2 mmol), $Pd(OAc)_2$ (4.5 mg, 0.02 mmol), 4,7-dimethoxy-1,10-phenanthroline (4.8 mg, 0.02 mmol) in 1.0 mL DMF in a 65 °C oil bath for 1 h. The product was purified by flash column chromatography on silica gel (ethyl acetate : hexane = 1 : 80) and obtained as a colorless oil (22.4 mg, 41% yield), R_f = 0.29 (ethyl acetate : hexane = 1 : 20). **^{1}H NMR** (500 MHz, $CDCl_3$): δ (ppm) 7.47 (d, *J* = 8.1 Hz, 2H), 7.36 – 7.25 (m, 5H), 7.18 – 7.15 (m, 2H), 5.84 (d, *J* = 33.3 Hz, 1H), 5.16 (s, 2H), 2.32 (s, 3H). **^{13}C NMR** (126 MHz, $CDCl_3$): δ (ppm) 167.2 (d, *J* = 278.2 Hz), 164.1 (d, *J* = 2.3 Hz), 142.4, 136.1, 129.7 (d, *J* = 1.8 Hz), 128.7, 128.4, 128.3, 127.8 (d, *J* = 25.9 Hz), 125.8 (d, *J* = 8.0 Hz), 96.0 (d, *J* = 6.7 Hz), 66.3, 21.7. **^{19}F NMR** (471 MHz, $CDCl_3$): δ (ppm) -98.04 (d, *J* = 33.1 Hz, 1F). **HRMS** (ESI) m/z: $[M+Na]^+$ Calcd for $C_{17}H_{15}FO_2Na$ 293.0948; Found 293.0945.

benzyl (*Z*)-3-fluoro-3-(*m*-tolyl)acrylate (2c)

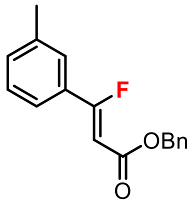

Following the general procedure (III), reaction was run using *gem*-difluoroalkene **1a** (39.6 mg, 0.2 mmol), (*m*-methylphenyl) boronic acid (27.2 mg, 0.2 mmol), $Pd(OAc)_2$ (4.5 mg, 0.02 mmol), 4,7-dimethoxy-1,10-phenanthroline (4.8 mg, 0.02 mmol) in 1.0 mL DMF in a 65 °C oil bath for 24 h. The product was purified by flash column chromatography on silica gel (ethyl acetate : hexane = 1 : 100) and obtained as a colorless oil (23.5 mg, 49% yield), R_f = 0.34 (ethyl acetate : hexane = 1 : 20). **^{1}H NMR** (500 MHz, $CDCl_3$): δ (ppm) 7.38 – 7.17 (m, 9H), 5.86 (d, *J* = 33.2 Hz, 1H), 5.16 (s, 2H), 2.30 (s, 3H). **^{13}C NMR** (126 MHz, $CDCl_3$): δ (ppm) 167.1 (d, *J* = 278.7 Hz), 164.0 (d, *J* = 2.3 Hz), 138.8 (d, *J* = 2.1 Hz), 136.1, 132.6, 130.6 (d, *J* = 25.8 Hz), 128.9 (d, *J* = 1.9 Hz), 128.7, 128.4, 128.3, 126.4 (d, *J* = 7.8 Hz), 123.0 (d, *J* = 8.1 Hz), 96.7 (d, *J* = 6.5 Hz), 66.3, 21.5. **^{19}F NMR** (471 MHz, $CDCl_3$): δ (ppm) -97.81 (d, *J* = 33.1 Hz, 1F). **HRMS** (ESI) m/z: $[M+Na]^+$ Calcd for $C_{17}H_{15}FO_2Na$ 293.0948; Found 293.0942.

benzyl (*Z*)-3-fluoro-3-(o-tolyl)acrylate (2d)

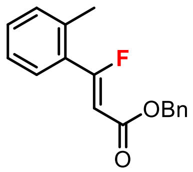

Following the general procedure (III), reaction was run using *gem*-difluoroalkene **1a** (39.6 mg, 0.2 mmol), (*o*-methylphenyl) boronic acid (27.2 mg, 0.2 mmol), $Pd(OAc)_2$ (4.5 mg, 0.02 mmol), 4,7-dimethoxy-1,10-phenanthroline (4.8 mg, 0.02 mmol) in 1.0 mL DMF in a 65 °C oil bath for 24 h. The product was purified by flash column chromatography on silica gel (CH_2Cl_2 : hexane = 1 : 10) and obtained as a colorless oil (16.5 mg, 30% yield), R_f = 0.30 (ethyl acetate : hexane = 1 : 20). **^{1}H NMR** (500 MHz, $CDCl_3$): δ (ppm) 7.36 – 7.25 (m, 7H), 7.18 – 7.14 (m, 2H), 5.56 (d, J = 31.7 Hz, 1H), 5.17 (s, 2H), 2.38 (d, J = 3.5 Hz, 3H). **^{13}C NMR** (126 MHz, $CDCl_3$): δ (ppm) 169.2 (d, J = 284.4 Hz), 163.8 (d, J = 2.8 Hz), 137.4, 136.0, 131.4, 131.2, 131.0 (d, J = 23.2 Hz), 129.1 (d, J = 5.8 Hz), 128.7, 128.4, 128.4, 126.1, 101.4 (d, J = 7.4 Hz), 66.3, 20.8 (d, J = 4.1 Hz). **^{19}F NMR** (471 MHz, $CDCl_3$): δ (ppm) -79.21 (dd, J = 31.6, 3.0 Hz, 1F). **HRMS** (ESI) m/z: $[M+Na]^+$ Calcd for $C_{17}H_{15}FO_2Na$ 293.0948; Found 293.0944.

benzyl (*Z*)-3-fluoro-3-(4-methoxyphenyl)acrylate (2e)

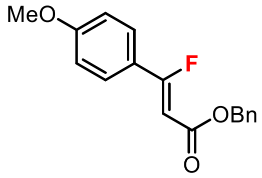

Following the general procedure (III), reaction was run using *gem*-difluoroalkene **1a** (39.6 mg, 0.2 mmol), (4-methoxyphenyl) boronic acid (30.4 mg, 0.2 mmol), $Pd(OAc)_2$ (4.5 mg, 0.02 mmol), 4,7-dimethoxy-1,10-phenanthroline (4.8 mg, 0.02 mmol) in 1.0 mL DMF in a 65 °C oil bath for 24 h. The product was purified by flash column chromatography on silica gel (ethyl acetate : hexane = 1 : 100) and obtained as a colorless oil (17.6 mg, 31% yield), R_f = 0.30 (ethyl acetate : hexane = 1 : 20). **^{1}H NMR** (500 MHz, $CDCl_3$): δ (ppm) 7.60 (d, J = 8.8 Hz, 2H), 7.43 – 7.32 (m, 5H), 6.93 (d, J = 8.6 Hz, 2H), 5.84 (d, J = 33.5 Hz, 1H), 5.23 (s, 2H), 3.85 (s, 3H). **^{13}C NMR** (126 MHz, $CDCl_3$): δ (ppm) 167.1 (d, J = 277.1 Hz), 164.2 (d, J = 2.2 Hz), 162.5, 136.2, 128.7, 128.4, 128.3, 127.6 (d, J = 8.4 Hz), 123.0 (d, J = 25.9 Hz), 114.4 (d, J = 1.9 Hz), 94.9 (d, J = 6.7 Hz), 66.2, 55.6. **^{19}F NMR** (471 MHz, $CDCl_3$): δ (ppm) -97.63 (d, J = 33.7 Hz, 1F). **HRMS** (ESI) m/z: $[M+Na]^+$ Calcd for $C_{17}H_{15}FO_3Na$ 309.0897; Found 309.0893.

benzyl (*Z*)-3-fluoro-3-(3-methoxyphenyl)acrylate (2f)

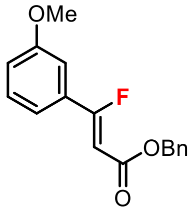

Following the general procedure (III), reaction was run using *gem*-difluoroalkene **1a** (39.6 mg, 0.2 mmol), (*m*-methoxyphenyl)boronic acid (30.4 mg, 0.2 mmol), $Pd(OAc)_2$ (4.5 mg, 0.02 mmol), 4,7-dimethoxy-1,10-phenanthroline (4.8 mg, 0.02 mmol) in 1.0 mL DMF in a 65 °C oil bath for 24 h. The product was purified by flash column chromatography on silica gel (ethyl acetate : hexane = 1 : 40) and obtained as a colorless oil (20.2 mg, 35% yield), R_f = 0.22 (ethyl acetate : hexane = 1 : 20). **^{1}H NMR** (500 MHz, $CDCl_3$): δ (ppm) 7.36 – 7.24 (m, 6H), 7.18 – 7.15 (m, 1H), 7.08 (s, 1H), 6.94 (d, J = 8.2 Hz, 1H), 5.87 (d, J = 33.0 Hz, 1H), 5.17 (s, 2H), 3.76 (s, 3H). **^{13}C NMR** (126 MHz, $CDCl_3$): δ (ppm) 166.7 (d, J = 278.7 Hz), 163.9 (d, J = 2.3 Hz), 160.0, 136.0, 132.0 (d, J = 26.3 Hz), 130.1 (d, J = 1.7 Hz), 128.7, 128.4, 128.4, 118.3 (d, J = 8.0 Hz), 117.7, 111.0 (d, J = 8.2 Hz), 97.2 (d, J = 6.8 Hz), 66.4, 55.5. **^{19}F NMR** (471 MHz, $CDCl_3$): δ (ppm) -98.38 (d, J = 33.0 Hz, 1F). **HRMS** (ESI) m/z: $[M+Na]^+$ Calcd for $C_{17}H_{15}FO_3Na$ 309.0897; Found 309.0892.

benzyl (*Z*)-3-fluoro-3-(4-fluorophenyl)acrylate (2g)

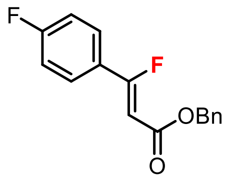

Following the general procedure (III), reaction was run using *gem*-difluoroalkene **1a** (39.6 mg, 0.2 mmol), (4-fluorophenyl) boronic acid (28.0 mg, 0.2 mmol), $Pd(OAc)_2$ (4.5 mg, 0.02 mmol), 4,7-dimethoxy-1,10-phenanthroline (4.8 mg, 0.02 mmol) in 1.0 mL DMF in a 65 °C oil bath for 24 h. The product was purified by flash column chromatography on silica gel (ethyl acetate : hexane = 1 : 50) and obtained as a colorless oil (19.7 mg, 36% yield), R_f = 0.33 (ethyl acetate : hexane = 1 : 20). ^{1}**H NMR** (500 MHz, $CDCl_3$): δ (ppm) 7.65 (dd, *J* = 8.8, 5.3 Hz, 2H), 7.43 – 7.33 (m, 5H), 7.12 (t, *J* = 8.5 Hz, 2H), 5.90 (d, *J* = 33.0 Hz, 1H), 5.24 (s, 2H). ^{13}**C NMR** (126 MHz, $CDCl_3$): δ (ppm) 165.9 (d, *J* = 277.9 Hz), 164.8 (d, *J* = 253.5 Hz), 163.8 (d, *J* = 2.2 Hz), 136.0, 128.7, 128.4, 128.1, 128.1 (d, *J* = 16.8 Hz), 126.9 (dd, *J* = 26.5, 3.5 Hz), 116.3 (dd, *J* = 22.2, 1.9 Hz), 96.8 (dd, *J* = 6.8, 1.9 Hz), 66.4. ^{19}**F NMR** (471 MHz, $CDCl_3$): δ (ppm) -97.23 (d, *J* = 33.0 Hz, 1F), -109.67 – -109.73 (m, 1F). **HRMS** (ESI) m/z: $[M+Na]^+$ Calcd for $C_{16}H_{12}F_2O_2Na$ 297.0698; Found 297.0693.

4-methoxybenzyl (*Z*)-3-fluoro-3-phenylacrylate (2h)

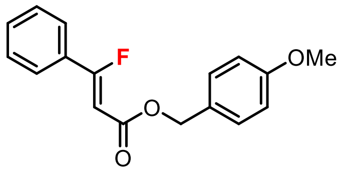

Following the general procedure (III), reaction was run using *gem*-difluoroalkene **1b** (45.6 mg, 0.2 mmol), phenylboronic acid (24.4 mg, 0.2 mmol), $Pd(OAc)_2$ (4.5 mg, 0.02 mmol), 4,7-dimethoxy-1,10-phenanthroline (4.8 mg, 0.02 mmol) in 1.0 mL DMF in a 65 °C oil bath for 24 h. The product was purified by flash column chromatography on silica gel (ethyl acetate : hexane = 1 : 50) and obtained as a colorless oil (34.0 mg, 59% yield), R_f = 0.20 (ethyl acetate : hexane = 1 : 20). ^{1}**H NMR** (500 MHz, $CDCl_3$): δ (ppm) 7.64 (d, *J* = 7.0 Hz, 2H), 7.49 – 7.36 (m, 5H), 6.91 (d, *J* = 8.7 Hz, 2H), 5.93 (d, *J* = 33.1 Hz, 1H), 5.18 (s, 2H), 3.82 (s, 3H).^{13}**C NMR** (126 MHz, $CDCl_3$): δ (ppm) 166.7 (d, *J* = 278.3 Hz), 164.0 (d, *J* = 2.2 Hz), 159.8, 131.7, 130.7 (d, *J* = 26.0 Hz), 130.3, 129.0 (d, *J* = 2.1 Hz), 128.2, 125.8 (d, *J* = 7.8 Hz), 114.1, 97.1 (d, *J* = 6.7 Hz), 66.2, 55.4. ^{19}**F NMR** (471 MHz, $CDCl_3$): δ (ppm) -98.26 (d, *J* = 33.3 Hz, 1F). **HRMS** (ESI) m/z: $[M+Na]^+$ Calcd for $C_{17}H_{15}FO_3Na$ 309.0897; Found 309.0893.

4-(trifluoromethyl)benzyl (*Z*)-3-fluoro-3-phenylacrylate (2i)

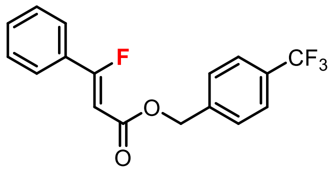

Following the general procedure (III), reaction was run using *gem*-difluoroalkene **1c** (53.2 mg, 0.2 mmol), phenylboronic acid (24.4 mg, 0.2 mmol), $Pd(OAc)_2$ (4.5 mg, 0.02 mmol), 4,7-dimethoxy-1,10-phenanthroline (4.8 mg, 0.02 mmol) in 1.0 mL DMF in a 65 °C oil bath for 4 h. The product was purified by flash column chromatography on silica gel (ethyl acetate : hexane = 1 : 60) and obtained as a colorless solid (27.0 mg, 42% yield), R_f = 0.35 (ethyl acetate : hexane = 1 : 20). ^{1}**H NMR** (500 MHz, $CDCl_3$): δ (ppm) 7.68 – 7.64 (m, 4H), 7.55 – 7.43 (m, 5H), 5.97 (d, *J* = 33.1 Hz, 1H), 5.30 (s, 2H). ^{13}**C NMR** (126 MHz, $CDCl_3$): δ (ppm) 167.3 (d, *J* = 279.3 Hz), 163.8 (d, *J* = 2.3 Hz), 140.1, 131.9, 130.5 (d, *J* = 25.9 Hz), 130.5 (q, *J* = 32.2 Hz), 129.1 (d, *J* = 2.1 Hz), 128.3, 125.9 (d, *J* = 8.0 Hz), 125.7 (q, *J* = 3.8 Hz), 124.2 (q, *J* = 272.1 Hz), 96.6 (d, *J* = 6.8 Hz), 65.4. ^{19}**F NMR** (471 MHz, $CDCl_3$): δ (ppm) -63.43 (s, 3F), -94.92 (d, *J* = 33.0 Hz, 1F). **HRMS** (ESI) m/z: $[M+Na]^+$ Calcd for $C_{17}H_{12}F_4O_2Na$ 347.0666; Found 347.0660.

heptyl (*Z*)-3-fluoro-3-phenylacrylate (2j)

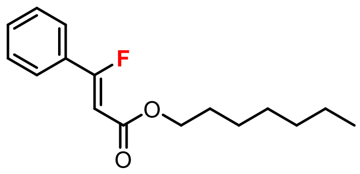

Following the general procedure (III), reaction was run using *gem*-difluoroalkene **1d** (41.2 mg, 0.2 mmol), phenylboronic acid (24.4 mg, 0.2 mmol), $Pd(OAc)_2$ (4.5 mg, 0.02 mmol), 4,7-dimethoxy-1,10-phenanthroline (4.8 mg, 0.02 mmol) in 1.0 mL DMF in a 65 ^oC oil bath for 9 h. The product was purified by flash column chromatography on silica gel (ethyl acetate : hexane = 1 : 100) and obtained as a colorless oil (22.7 mg, 43% yield), R_f = 0.36 (ethyl acetate : hexane = 1 : 50). **^{1}H NMR** (500 MHz, $CDCl_3$): δ (ppm) 7.67 – 7.64 (m, 2H), 7.50 – 7.42 (m, 3H), 5.90 (d, J = 33.4 Hz, 1H), 4.19 (t, J = 6.7 Hz, 2H), 1.72 – 1.64 (m, 2H), 1.42 – 1.27 (m, 8H), 0.89 (t, J = 6.9 Hz, 3H). **^{13}C NMR** (126 MHz, $CDCl_3$): δ (ppm) 166.4 (d, J = 277.6 Hz), 164.3 (d, J = 2.3 Hz), 131.6, 130.8 (d, J = 26.3 Hz), 129.0 (d, J = 1.9 Hz), 125.8 (d, J = 7.9 Hz), 97.3 (d, J = 6.8 Hz), 64.8, 31.9, 29.1, 28.8, 26.0, 22.7, 14.2. **^{19}F NMR** (471 MHz, $CDCl_3$): δ (ppm) -96.95 (d, J = 33.2 Hz, 1F). **HRMS** (ESI) m/z: $[M+Na]^+$ Calcd for $C_{16}H_{21}FO_2Na$ 287.1418; Found 287.1413.

4-methoxyphenyl (*Z*)-3-fluoro-3-phenylacrylate (2k)

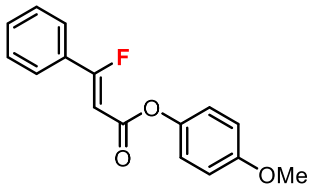

Following the general procedure (III), reaction was run using *gem*-difluoroalkene **1e** (42.8 mg, 0.2 mmol), phenylboronic acid (24.4 mg, 0.2 mmol), $Pd(OAc)_2$ (4.5 mg, 0.02 mmol), 4,7-dimethoxy-1,10-phenanthroline (4.8 mg, 0.02 mmol) in 1.0 mL DMF in a 65 ^oC oil bath for 6 h. The product was purified by flash column chromatography on silica gel (ethyl acetate : hexane = 1 : 100) and obtained as a colorless solid (21.0 mg, 38% yield), R_f = 0.21 (ethyl acetate : hexane = 1 : 20). **^{1}H NMR** (500 MHz, $CDCl_3$): δ (ppm) 7.72 (d, J = 8.2 Hz, 2H), 7.54 – 7.46 (m, 3H), 7.09 (d, J = 8.9 Hz, 2H), 6.92 (d, J = 8.9 Hz, 2H), 6.11 (d, J = 32.6 Hz, 1H), 3.81 (s, 3H). **^{13}C NMR** (126 MHz, $CDCl_3$): δ (ppm) 167.8 (d, J = 280.2 Hz), 162.8 (d, J = 2.8 Hz), 157.4, 144.1, 132.0, 130.5 (d, J = 25.6 Hz), 129.1 (d, J = 1.9 Hz), 126.0 (d, J = 8.1 Hz), 122.6, 114.6, 96.5 (d, J = 6.5 Hz), 55.7. **^{19}F NMR** (471 MHz, $CDCl_3$): δ (ppm) -95.05 (d, J = 32.6 Hz, 1F). **HRMS** (ESI) m/z: $[M+Na]^+$ Calcd for $C_{16}H_{13}FO_3Na$ 295.0741; Found 295.0736.

4-fluorophenyl (*Z*)-3-fluoro-3-phenylacrylate (2l)

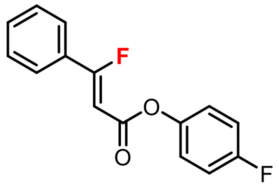

Following the general procedure (III), reaction was run using *gem*-difluoroalkene **1f** (40.4 mg, 0.2 mmol), phenylboronic acid (24.4 mg, 0.2 mmol), $Pd(OAc)_2$ (4.5 mg, 0.02 mmol), 4,7-dimethoxy-1,10-phenanthroline (4.8 mg, 0.02 mmol) in 1.0 mL DMF in a 65 ^oC oil bath for 3 h. The product was purified by flash column chromatography on silica gel (ethyl acetate : hexane = 1 : 100) and obtained as a colorless solid (9.1 mg, 17% yield), R_f = 0.28 (ethyl acetate : hexane = 1 : 20). **^{1}H NMR** (500 MHz, $CDCl_3$): δ (ppm) 7.73 (d, J = 7.6 Hz, 2H), 7.54 (t, J = 7.3 Hz, 1H), 7.48 (t, J = 7.5 Hz, 2H), 7.15 – 7.12 (m, 2H), 7.11 – 7.07 (m, 2H), 6.11 (d, J = 32.5 Hz, 1H). **^{13}C NMR** (126 MHz, $CDCl_3$): δ (ppm) 168.2 (d, J = 280.9 Hz), 162.4 (d, J = 2.5 Hz), 160.4 (d, J = 244.3 Hz), 146.4 (d, J = 2.7 Hz), 132.2, 130.4 (d, J = 25.6 Hz), 129.1 (d, J = 2.0 Hz), 126.0 (d, J = 8.1 Hz), 123.2 (d, J = 8.6 Hz), 116.2 (d, J = 23.5 Hz), 96.2 (d, J = 6.4 Hz). **^{19}F NMR** (471 MHz, $CDCl_3$): δ (ppm) -96.59 (d, J = 32.3 Hz, 1F), -121.45 – -121.51 (m, 1F). **HRMS** (ESI) m/z: $[M+Na]^+$ Calcd for $C_{15}H_{10}F_2O_2Na$ 283.0541; Found 283.0536.

benzyl (*E*)-3-fluoro-3-phenylacrylate (3a)

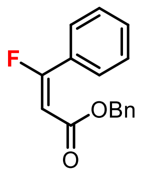

Following the general procedure (IV), reaction was run using *gem*-difluoroalkene **1a** (39.6 mg, 0.2 mmol), phenylboronic acid (36.6 mg, 0.3 mmol) and $Pd(PPh_3)_4$ (23.1 mg, 0.02 mmol) in 2.0 mL toluene at 90 °C for 18 h. The product was purified by flash column chromatography on silica gel (ethyl acetate : hexane = 1 : 50) and obtained as a colorless oil (31.0 mg, 60% yield), R_f = 0.27 (ethyl acetate : hexane = 1 : 20). **^{1}H NMR** (500 MHz, $CDCl_3$): δ (ppm) 7.70 (d, J = 7.5 Hz, 2H), 7.48 (t, J = 7.2 Hz, 1H), 7.41 (t, J = 7.6 Hz, 2H), 7.36 – 7.32 (m, 3H), 7.30 – 7.28 (m, 2H), 5.93 (d, J = 20.4 Hz, 1H), 5.13 (s, 2H). **^{13}C NMR** (126 MHz, $CDCl_3$): δ (ppm) 170.4 (d, J = 265.8 Hz), 165.2 (d, J = 24.5 Hz), 135.7, 131.5, 130.1 (d, J = 26.3 Hz), 129.2 (d, J = 6.0 Hz), 128.7, 128.4, 128.4, 128.1, 101.5 (d, J = 34.0 Hz), 66.4. **^{19}F NMR** (471 MHz, $CDCl_3$): δ (ppm) -77.33 (d, J = 20.4 Hz, 1F). The spectra are in full accordance with the literature report.[7]

benzyl (*E*)-3-fluoro-3-(o-tolyl)acrylate (3b)

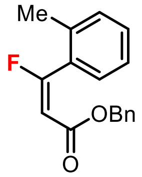

Following the general procedure (IV), reaction was run using *gem*-difluoroalkene **1a** (39.6 mg, 0.2 mmol), 2-methylphenylboronic acid (40.8 mg, 0.3 mmol) and $Pd(PPh_3)_4$ (23.1 mg, 0.02 mmol) in 2.0 mL toluene at 90 °C for 18 h. The product was purified by flash column chromatography on silica gel (CH_2Cl_2 : hexane = 1 : 10) and obtained as a colorless oil (35.5 mg, 66% yield), R_f = 0.24 (CH_2Cl_2 : hexane = 1 : 20). **^{1}H NMR** (500 MHz, $CDCl_3$): δ (ppm) 7.29 – 7.21 (m, 5H), 7.17 – 7.11 (m, 2H), 7.07 – 7.05 (m, 2H), 5.91 (d, J = 16.8 Hz, 1H), 4.96 (s, 2H), 2.25 (d, J = 2.7 Hz, 3H). **^{13}C NMR** (126 MHz, $CDCl_3$): δ (ppm) 171.9 (d, J = 272.5 Hz), 164.9 (d, J = 24.0 Hz), 137.1, 135.7, 130.8 (d, J = 2.8 Hz), 130.7 (d, J = 22.8 Hz), 130.3 (d, J = 1.9 Hz), 130.0 (d, J = 3.4 Hz), 128.6, 128.3, 128.2, 125.5 (d, J = 2.0 Hz), 103.5 (d, J = 32.3 Hz), 66.3, 19.5. **^{19}F NMR** (471 MHz, $CDCl_3$): δ (ppm) -68.48 (d, J = 16.8 Hz, 1F). **HRMS** (ESI) m/z: $[M+Na]^+$ Calcd for $C_{17}H_{15}FO_2Na$ 293.0948; Found 293.0945.

benzyl (*E*)-3-fluoro-3-(4-methoxyphenyl)acrylate (3c)

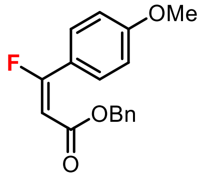

Following the general procedure (IV), reaction was run using *gem*-difluoroalkene **1a** (39.6 mg, 0.2 mmol), (4-methoxyphenyl) boronic acid (45.6 mg, 0.3 mmol) and $Pd(PPh_3)_4$ (23.1 mg, 0.02 mmol) in 2.0 mL toluene at 90 °C for 18 h. The product was purified by flash column chromatography on silica gel (ethyl acetate : hexane = 1 : 40) and obtained as a colorless oil (30.1 mg, 53% yield), R_f = 0.22 (ethyl acetate : hexane = 1 : 10). **^{1}H NMR** (500 MHz, $CDCl_3$): δ (ppm) 7.74 (d, J = 8.6 Hz, 2H), 7.39 – 7.32 (m, 5H), 6.91 (d, J = 8.6 Hz, 2H), 5.85 (d, J = 21.2 Hz, 1H), 5.15 (s, 2H), 3.85 (s, 3H). **^{13}C NMR** (126 MHz, $CDCl_3$): δ (ppm) 170.4 (d, J = 263.6 Hz), 165.5 (d, J = 25.2 Hz), 162.1, 135.9, 131.1 (d, J = 6.8 Hz), 128.7, 128.4, 128.3, 122.2 (d, J = 27.0 Hz), 113.5, 99.8 (d, J = 35.4 Hz), 66.3, 55.5. **^{19}F NMR** (471 MHz, $CDCl_3$): δ (ppm) -77.58 (d, J = 21.2 Hz, 1F). **HRMS** (ESI) m/z: $[M+Na]^+$ Calcd for $C_{17}H_{15}FO_3Na$ 309.0897; Found 309.0892.

benzyl (*E*)-3-fluoro-3-(3-methoxyphenyl)acrylate (3d)

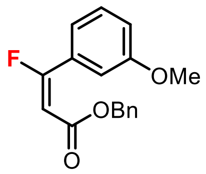

Following the general procedure (IV), reaction was run using *gem*-difluoroalkene **1a** (39.6 mg, 0.2 mmol), 3-methoxyphenylboronic acid (45.6 mg, 0.3 mmol) and $Pd(PPh_3)_4$ (23.1 mg, 0.02 mmol) in 2.0 mL toluene at 90 °C for 18 h. The product was purified by flash column chromatography on silica gel (CH_2Cl_2: hexane = 1 : 5) and obtained as a colorless oil (32.5 mg, 57% yield), R_f = 0.25 (CH_2Cl_2 : hexane = 1 : 2). **^{1}H NMR** (500 MHz, $CDCl_3$) δ (ppm) 7.30 – 7.16 (m, 8H), 6.93 (d, J = 7.7 Hz, 1H), 5.84 (d, J = 20.5 Hz, 1H), 5.05 (s, 2H), 3.71 (s, 3H). **^{13}C NMR** (126 MHz, $CDCl_3$) δ (ppm) 169.9 (d, J = 265.7 Hz), 165.1 (d, J = 24.7 Hz), 159.1, 135.7, 131.3, 131.1, 129.2, 128.6, 128.4, 121.6 (d, J = 6.3 Hz), 117.6, 114.2 (d, J = 6.2 Hz), 101.7 (d, J = 34.1 Hz), 66.5, 55.4. **^{19}F NMR** (471 MHz, $CDCl_3$) δ (ppm) -77.29 (d, J = 20.6 Hz, 1F). **HRMS** (ESI) m/z: $[M+Na]^+$ Calcd for $C_{17}H_{15}FO_3Na$ 309.0897; Found 309.0894.

benzyl (*E*)-3-fluoro-3-(2-methoxyphenyl)acrylate (3e)

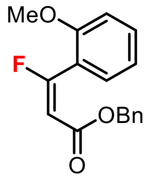

Following the general procedure (IV), reaction was run using *gem*-difluoroalkene **1a** (39.6 mg, 0.2 mmol), 2-methoxyphenylboronic acid (45.6 mg, 0.3 mmol) and $Pd(PPh_3)_4$ (23.1 mg, 0.02 mmol) in 2.0 mL toluene at 80 °C for 18 h. The product was purified by flash column chromatography on silica gel (CH_2Cl_2: hexane = 1 : 5) and obtained as a colorless oil (25.8 mg, 45% yield), R_f = 0.22 (CH_2Cl_2 : hexane = 1 : 2). **^{1}H NMR** (500 MHz, $CDCl_3$) δ (ppm) 7.45 – 7.41 (m, 1H), 7.37 (dt, J = 7.6, 1.3 Hz, 1H), 7.33 – 7.27 (m, 3H), 7.20 – 7.17 (m, 2H), 6.99 (t, J = 7.5 Hz, 1H), 6.90 (d, J = 8.4 Hz, 1H), 5.98 (d, J = 16.6 Hz, 1H), 5.07 (s, 2H), 3.74 (s, 3H). **^{13}C NMR** (126 MHz, $CDCl_3$) δ (ppm) 168.5 (d, J = 269.6 Hz), 165.1 (d, J = 24.0 Hz), 157.4 (d, J = 2.3 Hz), 135.9, 132.4 (d, J = 2.5 Hz), 130.8 (d, J = 3.2 Hz), 128.5, 128.2, 128.2, 120.3 (d, J = 1.7 Hz), 120.2 (d, J = 24.5 Hz), 111.2, 103.7 (d, J = 32.3 Hz), 66.1, 55.7. **^{19}F NMR** (471 MHz, $CDCl_3$) δ (ppm) -73.98 (d, J = 16.5 Hz, 1F). **HRMS** (ESI) m/z: $[M+Na]^+$ Calcd for $C_{17}H_{15}FO_3Na$ 309.0897; Found 309.0892.

benzyl (*E*)-3-fluoro-3-(4-(trifluoromethyl)phenyl)acrylate (3f)

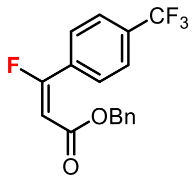

Following the general procedure (IV), reaction was run using *gem*-difluoroalkene **1a** (39.6 mg, 0.2 mmol), [p-(trifluoromethyl)phenyl]boronic acid (57.0 mg, 0.3 mmol) and $Pd(PPh_3)_4$ (23.1 mg, 0.02 mmol) in 2.0 mL toluene at 90 °C for 18 h. The product was purified by flash column chromatography on silica gel (ethyl acetate : hexane = 1 : 100) and obtained as a yellow oil (34.0 mg, 52% yield), R_f = 0.23 (ethyl acetate : hexane = 1 : 20). **^{1}H NMR** (500 MHz, $CDCl_3$): δ (ppm) 7.71 (d, J = 8.1 Hz, 2H), 7.57 (d, J = 8.1 Hz, 2H), 7.27 – 7.18 (m, 5H), 5.94 (d, J = 20.0 Hz, 1H), 5.06 (s, 2H). **^{13}C NMR** (101 MHz, $CDCl_3$): δ (ppm) 168.6 (d, J = 266.0 Hz), 164.7 (d, J = 23.9 Hz), 135.4, 133.4 (d, J = 26.7 Hz), 133.0 (d, J = 33.1 Hz), 129.6 (d, J = 5.6 Hz), 128.7, 128.6, 128.5, 125.1 (q, J = 3.8 Hz), 125.1 (q, J = 272.5 Hz), 103.2 (d, J = 32.6 Hz), 66.8. **^{19}F NMR** (471 MHz, $CDCl_3$): δ (ppm) -65.40 (s, 3F), -78.78 (d, J = 20.0 Hz, 1F). **HRMS** (ESI) m/z: $[M+Na]^+$ Calcd for $C_{17}H_{12}F_4O_2Na$ 347.0666; Found 347.0658.

benzyl (*E*)-3-fluoro-3-(4-fluorophenyl)acrylate (3g)

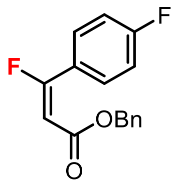

Following the general procedure (IV), reaction was run using *gem*-difluoroalkene **1a** (39.6 mg, 0.2 mmol), 4-fluorophenylboronic acid (42.0 mg, 0.3 mmol) and $Pd(PPh_3)_4$ (23.1 mg, 0.02 mmol) in 2.0 mL toluene at 80 °C for 18 h. The product was purified by flash column chromatography on silica gel (CH_2Cl_2: hexane = 1 : 10) and obtained as a colorless solid (25.8 mg, 47% yield), R_f = 0.23 (CH_2Cl_2 : hexane = 1 : 5). **^{1}H NMR** (500 MHz, $CDCl_3$): δ (ppm) 7.73 (dd, *J* = 8.7, 5.5 Hz, 2H), 7.38 – 7.30 (m, 5H), 7.08 (t, *J* = 8.5 Hz, 2H), 5.92 (d, *J* = 20.6 Hz, 1H), 5.14 (s, 2H). **^{13}C NMR** (126 MHz, $CDCl_3$): δ (ppm) 169.3 (d, *J* = 264.9 Hz), 165.1 (d, *J* = 24.6 Hz), 164.5 (dd, *J* = 252.9, 1.3 Hz), 135.7, 131.6 (dd, *J* = 8.9, 6.2 Hz), 128.7, 128.5, 128.5, 126.1 (dd, *J* = 27.4, 3.4 Hz), 115.3 (d, *J* = 22.1 Hz), 101.4 (d, *J* = 34.1 Hz), 66.5. **^{19}F NMR** (471 MHz, $CDCl_3$) δ (ppm) -77.32 (d, *J* = 20.3 Hz, 1F), -109.79 – -109.84 (m, 1F). **HRMS** (ESI) m/z: $[M+Na]^+$ Calcd for $C_{16}H_{12}F_2O_2Na$ 297.0698; Found 297.0695.

benzyl (*E*)-3-fluoro-3-(2-fluorophenyl)acrylate (3h)

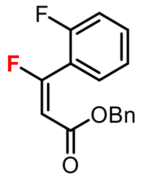

Following the general procedure (IV), reaction was run using *gem*-difluoroalkene **1a** (39.6 mg, 0.2 mmol), 2-Fluorophenylboronic acid (42.0 mg, 0.3 mmol) and $Pd(PPh_3)_4$ (23.1 mg, 0.02 mmol) in 2.0 mL toluene at 90 °C for 18 h. The product was purified by flash column chromatography on silica gel (CH_2Cl_2: hexane = 1 : 10) and obtained as a colorless oil (25.8 mg, 47% yield), R_f = 0.25 (CH_2Cl_2 : hexane = 1 : 3). **^{1}H NMR** (500 MHz, $CDCl_3$): δ (ppm) 7.40 – 7.10 (m, 8H), 7.01 (t, *J* = 9.1 Hz, 1H), 5.97 (d, *J* = 16.9 Hz, 1H), 5.02 (s, 2H). **^{13}C NMR** (126 MHz, $CDCl_3$): δ (ppm) 165.3 (d, *J* = 268.2 Hz), 164.6 (d, *J* = 23.3 Hz), 160.1 (dd, *J* = 253.5, 3.4 Hz), 135.6, 133.0 (dd, *J* = 8.6, 2.1 Hz), 130.6 (t, *J* = 2.5 Hz), 128.6, 128.4, 128.3, 123.9 (d, *J* = 3.8 Hz), 119.2 (dd, *J* = 26.6, 14.3 Hz), 116.0 (d, *J* = 21.5 Hz), 104.7 (d, *J* = 31.1 Hz), 66.5. **^{19}F NMR** (471 MHz, $CDCl_3$): δ (ppm) -76.79 (dd, *J* = 17.0, 7.5 Hz, 1F), -113.06 – -113.12 (m, 1F). **HRMS** (ESI) m/z: $[M+Na]^+$ Calcd for $C_{16}H_{12}F_2O_2Na$ 297.0698; Found 297.0695.

benzyl (*E*)-3-(4-chlorophenyl)-3-fluoroacrylate (3i)

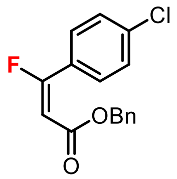

Following the general procedure (IV), reaction was run using *gem*-difluoroalkene **1a** (39.6 mg, 0.2 mmol), 4-chlorophenylboronic acid (46.9 mg, 0.3 mmol) and $Pd(PPh_3)_4$ (23.1 mg, 0.02 mmol) in 2.0 mL toluene at 80 °C for 18 h. The product was purified by flash column chromatography on silica gel (CH_2Cl_2: hexane = 1 : 10) and obtained as a colorless solid (24.8 mg, 43% yield), R_f = 0.22 (CH_2Cl_2 : hexane = 1 : 4). **^{1}H NMR** (500 MHz, $CDCl_3$): δ (ppm) 7.65 (d, *J* = 8.6 Hz, 2H), 7.38 – 7.29 (m, 7H), 5.94 (d, *J* = 20.5 Hz, 1H), 5.13 (s, 2H). **^{13}C NMR** (126 MHz, $CDCl_3$): δ (ppm) 169.14 (d, *J* = 264.9 Hz), 164.99 (d, *J* = 24.4 Hz), 137.66 (d, *J* = 2.0 Hz), 135.59, 130.59 (d, *J* = 6.1 Hz), 128.72, 128.52, 128.49, 128.45, 128.29, 102.00 (d, *J* = 33.7 Hz), 66.60. **^{19}F NMR** (471 MHz, $CDCl_3$): δ (ppm) -78.31 (d, *J* = 20.5 Hz, 1F). **HRMS** (ESI) m/z: $[M+Na]^+$ Calcd for $C_{16}H_{12}FClO_2Na$ 313.0402; Found 313.0398.

methyl (*E*)-4-(3-(benzyloxy)-1-fluoro-3-oxoprop-1-en-1-yl)benzoate (3j)

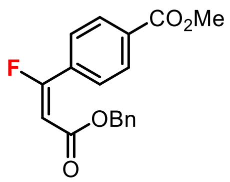

Following the general procedure (IV), reaction was run using *gem*-difluoroalkene **1a** (39.6 mg, 0.2 mmol), 4-methoxycarbonylphenylboronic acid (54.0 mg, 0.3 mmol) and $Pd(PPh_3)_4$ (23.1 mg, 0.02 mmol) in 2.0 mL toluene at 80 °C for 18 h. The product was purified by flash column chromatography on silica gel (CH_2Cl_2: hexane = 1 : 5) and obtained as a colorless solid (28.2 mg, 45% yield), R_f = 0.15 (CH_2Cl_2 : hexane = 1 : 2). **^{1}H NMR** (500 MHz, $CDCl_3$): δ (ppm) 7.98 (d, J = 8.1 Hz, 2H), 7.67 (d, J = 8.3 Hz, 2H), 7.29 – 7.18 (m, 5H), 5.92 (d, J = 20.2 Hz, 1H), 5.05 (s, 2H), 3.87 (s, 3H). **^{13}C NMR** (126 MHz, $CDCl_3$): δ (ppm) 169.0 (d, J = 266.0 Hz), 166.4, 164.8 (d, J = 24.3 Hz), 135.5, 134.1 (d, J = 26.5 Hz), 132.5 (d, J = 1.3 Hz), 129.2, 129.2, 129.2, 128.7, 128.5, 102.9 (d, J = 33.1 Hz), 66.7, 52.5. **^{19}F NMR** (471 MHz, $CDCl_3$): δ (ppm) -79.02 (d, J = 20.4 Hz, 1F). **HRMS** (ESI) m/z: $[M+Na]^+$ Calcd for $C_{18}H_{15}FO_4Na$ 337.0847; Found 337.0840.

benzyl (*E*)-3-(4-acetylphenyl)-3-fluoroacrylate (3k)

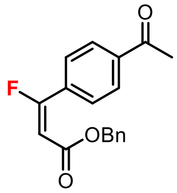

Following the general procedure (IV), reaction was run using *gem*-difluoroalkene **1a** (39.6 mg, 0.2 mmol), 4-acetylphenylboronic acid (49.2 mg, 0.3 mmol) and $Pd(PPh_3)_4$ (23.1 mg, 0.02 mmol) in 2.0 mL toluene at 90 °C for 18 h. The product was purified by flash column chromatography on silica gel (CH_2Cl_2: hexane = 1 : 5) and obtained as a colorless solid (26.2 mg, 44% yield), R_f = 0.15 (CH_2Cl_2 : hexane = 1 : 1). **^{1}H NMR** (500 MHz, $CDCl_3$): 7.96 (d, J = 8.0 Hz, 2H), 7.77 (d, J = 8.3 Hz, 2H), 7.37 – 7.26 (m, 5H), 6.01 (d, J = 20.1 Hz, 1H), 5.13 (s, 2H), 2.62 (s, 3H). **^{13}C NMR** (126 MHz, $CDCl_3$): δ (ppm) 197.4, 169.0 (d, J = 266.0 Hz), 164.8 (d, J = 23.9 Hz), 138.9, 135.5, 134.2 (d, J = 26.5 Hz), 129.5 (d, J = 5.6 Hz), 128.7, 128.5, 127.9, 103.0 (d, J = 32.8 Hz), 66.7, 26.9. **^{19}F NMR** (471 MHz, $CDCl_3$) δ (ppm) -78.70 (d, J = 20.2 Hz, 1F). **HRMS** (ESI) m/z: $[M+Na]^+$ Calcd for $C_{18}H_{15}FO_3Na$ 321.0897; Found 321.0894.

benzyl (*E*)-3-fluoro-3-(4-formylphenyl)acrylate (3l)

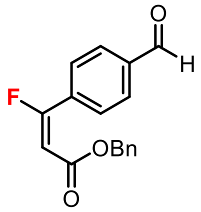

Following the general procedure (IV), reaction was run using *gem*-difluoroalkene **1a** (39.6 mg, 0.2 mmol), 4-formylphenylboronic acid (45.0 mg, 0.3 mmol) and $Pd(PPh_3)_4$ (23.1 mg, 0.02 mmol) in 2.0 mL toluene at 90 °C for 18 h. The product was purified by flash column chromatography on silica gel (CH_2Cl_2: hexane = 1 : 5) and obtained as a colorless solid (27.3 mg, 48% yield), R_f = 0.17 (CH_2Cl_2 : hexane = 1 : 2). **^{1}H NMR** (500 MHz, $CDCl_3$): δ (ppm) 10.05 (s, 1H), 7.89 (d, J = 8.0 Hz, 2H), 7.83 (d, J = 8.3 Hz, 2H), 7.37 – 7.26 (m, 5H), 6.04 (d, J = 20.0 Hz, 1H), 5.13 (s, 2H). **^{13}C NMR** (126 MHz, $CDCl_3$): δ (ppm) 191.6, 168.6 (d, J = 265.9 Hz), 164.7 (d, J = 23.9 Hz), 137.9, 135.4 (d, J = 26.5 Hz), 135.4, 129.8 (d, J = 5.6 Hz), 129.2, 128.7, 128.6, 128.5, 103.4 (d, J = 32.7 Hz), 66.8. **^{19}F NMR** (471 MHz, $CDCl_3$) δ (ppm) -79.68 (d, J = 19.9 Hz, 1F). **HRMS** (ESI) m/z: $[M+CH_3OH+Na]^+$ Calcd for $C_{18}H_{17}FO_4Na$ 339.1003; Found 339.1000.

benzyl (*E*)-3-fluoro-3-(naphthalen-2-yl)acrylate (3m)

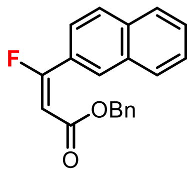

Following the general procedure (IV), reaction was run using *gem*-difluoroalkene **1a** (39.6 mg, 0.2 mmol), 2-fluorophenylboronic acid (51.6 mg, 0.3 mmol) and $Pd(PPh_3)_4$ (23.1 mg, 0.02 mmol) in 2.0 mL toluene at 80 °C for 18 h. The product was purified by flash column chromatography on silica gel (CH_2Cl_2: hexane = 1 : 10) and obtained as a colorless oil (39.4 mg, 64% yield), R_f = 0.26 (CH_2Cl_2 : hexane = 1 : 3). **^{1}H NMR** (500 MHz, $CDCl_3$): δ (ppm) 8.14 (s, 1H), 7.75 – 7.71 (m, 3H), 7.61 (d, *J* = 8.5 Hz, 1H), 7.46 – 7.39 (m, 2H), 7.17 (d, *J* = 9.5 Hz, 5H), 5.90 (d, *J* = 20.4 Hz, 1H), 5.03 (s, 2H). **^{13}C NMR** (126 MHz, $CDCl_3$): δ (ppm) 170.4 (d, *J* = 265.3 Hz), 165.2 (d, *J* = 24.6 Hz), 135.7, 134.6, 132.4, 130.1 (d, *J* = 6.9 Hz), 129.1, 128.6, 128.4, 128.4, 128.0, 127.8, 127.7, 127.4 (d, *J* = 26.3 Hz), 126.7, 125.3 (d, *J* = 5.4 Hz), 101.7 (d, *J* = 34.1 Hz), 66.5. **^{19}F NMR** (471 MHz, $CDCl_3$): δ (ppm) -76.87 (d, *J* = 20.5 Hz, 1F). **HRMS** (ESI) m/z: $[M+Na]^+$ Calcd for $C_{20}H_{15}FO_2Na$ 329.0948; Found 329.0947.

benzyl (*E*)-3-fluoro-3-(thiophen-3-yl)acrylate (3n)

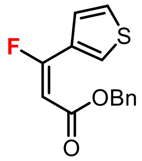

Following the general procedure (IV), reaction was run using *gem*-difluoroalkene **1a** (39.6 mg, 0.2 mmol), 3-thienylboronic acid (38.4 mg, 0.3 mmol) and $Pd(PPh_3)_4$ (23.1 mg, 0.02 mmol) in 2.0 mL toluene at 80 °C for 18 h. The product was purified by flash column chromatography on silica gel (CH_2Cl_2: hexane = 1 : 10) and obtained as a colorless oil (27.4 mg, 52% yield), R_f = 0.34 (CH_2Cl_2 : hexane = 1 : 5). **^{1}H NMR** (500 MHz, $CDCl_3$) δ (ppm) 8.38 (dd, *J* = 3.1, 1.2 Hz, 1H), 7.67 (dd, *J* = 5.2, 1.2 Hz, 1H), 7.39 – 7.31 (m, 6H), 5.86 (d, *J* = 23.4 Hz, 1H), 5.20 (s, 2H). **^{13}C NMR** (126 MHz, $CDCl_3$) δ (ppm) 165.4 (d, *J* = 25.7 Hz), 165.2 (d, *J* = 256.7 Hz), 135.9, 131.4 (d, *J* = 9.4 Hz), 131.0 (d, *J* = 30.4 Hz), 128.7, 128.4, 128.4, 127.7 (d, *J* = 7.1 Hz), 125.3, 99.7 (d, *J* = 35.8 Hz), 66.4. **^{19}F NMR** (471 MHz, $CDCl_3$) δ (ppm) -82.31 (d, *J* = 23.1 Hz, 1F). **HRMS** (ESI) m/z: $[M+Na]^+$ Calcd for $C_{14}H_{11}FO_2SNa$ 285.0356; Found 285.0355.

benzyl (*E*)-3-fluoro-3-(furan-3-yl)acrylate (3o)

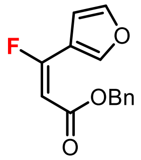

Following the general procedure (IV), reaction was run using *gem*-difluoroalkene **1a** (39.6 mg, 0.2 mmol), 3-furanylboronic acid (33.6 mg, 0.3 mmol) and $Pd(PPh_3)_4$ (23.1 mg, 0.02 mmol) in 2.0 mL toluene at 80 °C for 18 h. The product was purified by flash column chromatography on silica gel (CH_2Cl_2: hexane = 1 : 20) and obtained as a colorless oil (24.6 mg, 50% yield), R_f = 0.33 (CH_2Cl_2 : hexane = 1 : 5). **^{1}H NMR** (500 MHz, $CDCl_3$): δ (ppm) 8.47 (s, 1H), 7.45 (q, *J* = 1.7 Hz, 1H), 7.39 – 7.32 (m, 5H), 6.98 (d, *J* = 1.4 Hz, 1H), 5.83 (d, *J* = 23.1 Hz, 1H), 5.20 (s, 2H). **^{13}C NMR** (126 MHz, $CDCl_3$) δ (ppm) 165.4 (d, *J* = 25.5 Hz), 164.4 (d, *J* = 252.1 Hz), 147.5 (d, *J* = 12.6 Hz), 143.0, 136.0, 128.7, 128.4, 128.4, 117.3 (d, *J* = 33.1 Hz), 109.6 (d, *J* = 7.9 Hz), 99.8 (d, *J* = 34.9 Hz), 66.3. **^{19}F NMR** (471 MHz, $CDCl_3$) δ (ppm) -87.99 (dd, *J* = 23.3, 1.2 Hz, 1F). **HRMS** (ESI) m/z: $[M+Na]^+$ Calcd for $C_{14}H_{11}FO_3Na$ 269.0584; Found 269.0580.

heptyl (*E*)-3-fluoro-3-phenylacrylate (3p)

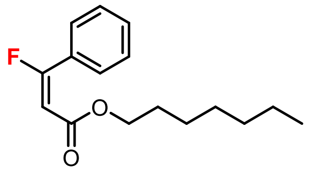

Following the general procedure (IV), reaction was run using *gem*-difluoroalkene **1d** (41.2 mg, 0.2 mmol), phenylboronic acid (36.6 mg, 0.3 mmol) and $Pd(PPh_3)_4$ (23.1 mg, 0.02 mmol) in 2.0 mL toluene at 80 °C for 18 h. The product was purified by flash column chromatography on silica gel (CH_2Cl_2: hexane = 1 : 10) and obtained as a colorless oil (24.3 mg, 46% yield), R_f = 0.24 (CH_2Cl_2 : hexane = 1 : 3). **^{1}H NMR** (500 MHz, $CDCl_3$): δ (ppm) 7.70 (d, J = 7.6 Hz, 2H), 7.49 – 7.41 (m, 3H), 5.88 (d, J = 20.5 Hz, 1H), 4.07 (t, J = 6.7 Hz, 2H), 1.57 (t, J = 6.8 Hz, 2H), 1.31 – 1.26 (m, 8H), 0.88 (t, J = 6.8 Hz, 3H). **^{13}C NMR** (126 MHz, $CDCl_3$): δ (ppm) 169.9 (d, J = 265.0 Hz), 165.5 (d, J = 24.4 Hz), 131.3 (d, J = 1.7 Hz), 130.3 (d, J = 26.4 Hz), 129.2 (d, J = 6.0 Hz), 128.0, 101.8 (d, J = 33.5 Hz), 64.9, 31.8, 29.0, 28.6, 26.0, 22.7, 14.2. **^{19}F NMR** (471 MHz, $CDCl_3$): δ (ppm) -78.27 (d, J = 20.7 Hz, 1F). **HRMS** (ESI) m/z: $[M+Na]^+$ Calcd for $C_{16}H_{21}FO_2Na$ 287.1418; Found 287.1415.

4-(trifluoromethyl)benzyl (*E*)-3-fluoro-3-phenylacrylate (3q)

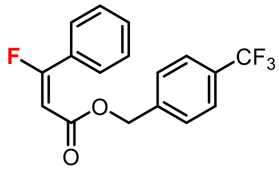

Following the general procedure (IV), reaction was run using *gem*-difluoroalkene **1c** (53.2 mg, 0.2 mmol), phenylboronic acid (36.6 mg, 0.3 mmol) and $Pd(PPh_3)_4$ (23.1 mg, 0.02 mmol) in 2.0 mL toluene at 90 °C for 18 h. The product was purified by flash column chromatography on silica gel (CH_2Cl_2 : hexane = 1 : 20) and obtained as a colorless solid (23.1 mg, 36% yield), R_f = 0.25 (CH_2Cl_2 : hexane = 1 : 2). **^{1}H NMR** (500 MHz, $CDCl_3$): δ (ppm) 7.69 (d, J = 7.3 Hz, 2H), 7.59 (d, J = 8.0 Hz, 2H), 7.49 (t, J = 7.4 Hz, 1H), 7.43 – 7.36 (m, 4H), 5.94 (d, J = 20.0 Hz, 1H), 5.17 (s, 2H). **^{13}C NMR** (126 MHz, $CDCl_3$): δ (ppm) 170.9 (d, J = 267.0 Hz), 165.0 (d, J = 24.6 Hz), 139.8, 131.6 (d, J = 1.7 Hz), 130.5 (d, J = 32.6 Hz), 130.0 (d, J = 26.1 Hz), 129.2 (d, J = 5.9 Hz), 128.3, 128.2, 125.6 (q, J = 3.8 Hz), 124.1 (q, J = 272.0 Hz), 101.2 (d, J = 34.3 Hz), 65.4. **^{19}F NMR** (471 MHz, $CDCl_3$): δ (ppm) -63.45 (s, 3F), -74.19 (d, J = 20.1 Hz, 1F). **HRMS** (ESI) m/z: $[M+Na]^+$ Calcd for $C_{17}H_{12}F_4O_2Na$ 347.0666; Found 347.0660.

4-methoxybenzyl (*E*)-3-fluoro-3-phenylacrylate (3r)

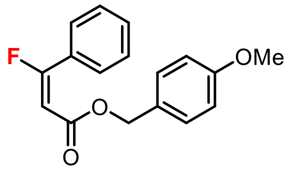

Following the general procedure (IV), reaction was run using *gem*-difluoroalkene **1b** (45.6 mg, 0.2 mmol), phenylboronic acid (36.6 mg, 0.3 mmol) and $Pd(PPh_3)_4$ (23.1 mg, 0.02 mmol) in 2.0 mL toluene at 80 °C for 18 h. The product was purified by flash column chromatography on silica gel (ethyl acetate : hexane = 1 : 60) and obtained as a colorless oil (25.9 mg, 45% yield), R_f = 0.23 (ethyl acetate : hexane = 1 : 20). **^{1}H NMR** (500 MHz, $CDCl_3$): δ (ppm) 7.61 (d, J = 7.5 Hz, 2H), 7.40 (t, J = 7.0 Hz, 1H), 7.33 (t, J = 7.8 Hz, 2H), 7.18 – 7.15 (m, 2H), 6.79 (d, J = 8.7 Hz, 2H), 5.82 (d, J = 20.4 Hz, 1H), 4.99 (s, 2H), 3.73 (s, 3H). **^{13}C NMR** (126 MHz, $CDCl_3$): δ (ppm) 170.2 (d, J = 265.3 Hz), 165.2 (d, J = 24.6 Hz), 159.8, 131.4 (d, J = 1.7 Hz), 130.3, 130.1 (d, J = 26.3 Hz), 129.2 (d, J = 6.0 Hz), 128.1, 127.9, 114.0, 101.6 (d, J = 33.9 Hz), 66.3, 55.4. **^{19}F NMR** (471 MHz, $CDCl_3$): δ (ppm) -77.82 (d, J = 20.5 Hz, 1F). **HRMS** (ESI) m/z: $[M+Na]^+$ Calcd for $C_{17}H_{15}FO_3Na$ 309.0897; Found 309.0893.

4-methoxyphenyl (*E*)-3-fluoro-3-phenylacrylate (3s)

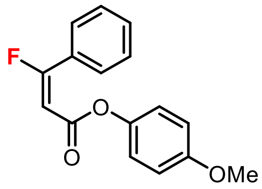

Following the general procedure (IV), reaction was run using *gem*-difluoroalkene **1e** (42.8 mg, 0.2 mmol), phenylboronic acid (36.6 mg, 0.3 mmol) and $Pd(PPh_3)_4$ (23.1 mg, 0.02 mmol) in 2.0 mL toluene at 80 °C for 18 h. The product was purified by flash column chromatography on silica gel (CH_2Cl_2: hexane = 1 : 10) and obtained as a colorless oil (20.2 mg, 37% yield), R_f =

0.21 (CH_2Cl_2 : hexane = 1 : 2). **^{1}H NMR** (500 MHz, $CDCl_3$) δ (ppm) 7.80 (d, J = 7.4 Hz, 2H), 7.49 (t, J = 7.3 Hz, 1H), 7.43 (t, J = 7.6 Hz, 2H), 7.05 – 6.99 (m, 2H), 6.91 – 6.86 (m, 2H), 6.10 (d, J = 20.4 Hz, 1H), 3.78 (s, 3H). **^{13}C NMR** (126 MHz, $CDCl_3$) δ (ppm) 171.4 (d, J = 267.1 Hz), 164.3 (d, J = 25.2 Hz), 157.4, 144.0, 131.8 (d, J = 1.2 Hz), 129.8 (d, J = 26.2 Hz), 129.3 (d, J = 6.5 Hz), 128.1, 122.4, 114.5, 101.0 (d, J = 35.1 Hz), 55.7. **^{19}F NMR** (471 MHz, $CDCl_3$) δ (ppm) -75.96 (d, J = 20.6 Hz, 1F). **HRMS** (ESI) m/z: $[M+Na]^+$ Calcd for $C_{16}H_{13}FO_3Na$ 295.0741; Found 295.0738.

(*E*)-3-fluoro-*N*,*N*,3-triphenylacrylamide (5)

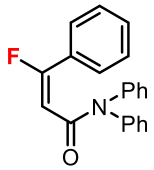

Following the general procedure (IV), reaction was run using *gem*-difluoroalkene **1g** (51.8 mg, 0.2 mmol), phenylboronic acid (36.6 mg, 0.3 mmol) and $Pd(PPh_3)_4$ (23.1 mg, 0.02 mmol) in 1.0 mL toluene at 80 °C for 18 h. The product was purified by flash column chromatography on silica gel (ethyl acetate: hexane = 1 : 10) and obtained as a colorless oil (24.5 mg, 39% yield), R_f = 0.22 (ethyl acetate : hexane = 1 : 10). **^{1}H NMR** (500 MHz, $CDCl_3$): δ (ppm) 7.73 – 7.71 (m, 2H), 7.45 – 7.19 (m, 13H), 5.84 (d, J = 21.6 Hz, 1H). **^{13}C NMR** (126 MHz, $CDCl_3$): δ (ppm) 166.3 (d, J = 259.5 Hz), 165.0 (d, J = 20.5 Hz), 142.6, 131.0, 130.4 (d, J = 27.3 Hz), 129.5 (broad), 128.6 (d, J = 6.4 Hz), 128.2, 126.5 (broad), 104.3 (d, J = 31.9 Hz). **^{19}F NMR** (471 MHz, $CDCl_3$): δ (ppm) -84.90 (d, J = 21.7 Hz, 1F). The spectra are in full accordance with the literature report.[7]

XI. Spectra.

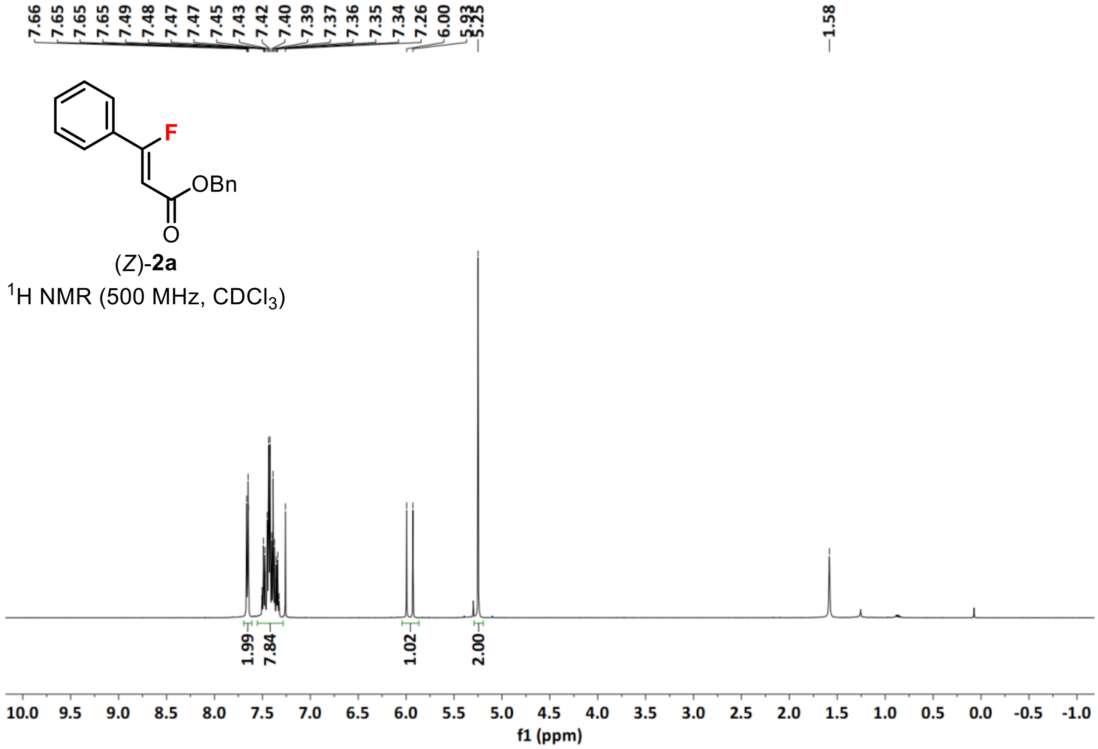

7.66 7.65 7.65 7.65 7.49 7.48 7.47 7.47 7.45 7.43 7.42 7.40 7.39 7.37 7.36 7.35 7.34 7.26 6.00 5.93 5.25
1.58
F
OBn
O
(Z)-2a
1H NMR (500 MHz, CDCl3)
1.99 7.84 1.02 2.00
10.0 9.5 9.0 8.5 8.0 7.5 7.0 6.5 6.0 5.5 5.0 4.5 4.0 3.5 3.0 2.5 2.0 1.5 1.0 0.5 0.0 -0.5 -1.0
f1 (ppm)

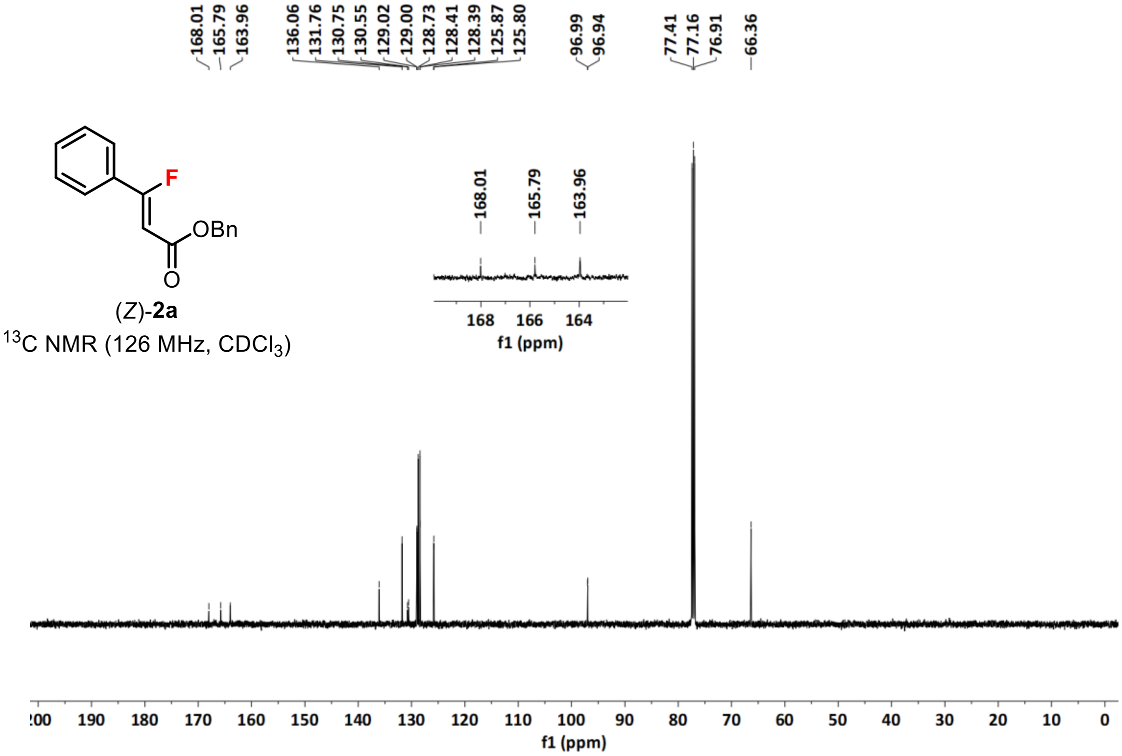

168.01 165.79 163.96
136.06 131.76 130.75 130.55 129.02 129.00 128.73 128.41 128.39 125.87 125.80
96.99 96.94
77.41 77.16 76.91
66.36
F
OBn
O
(Z)-2a
13C NMR (126 MHz, CDCl3)
168.01 165.79 163.96
168 166 164
f1 (ppm)
200 190 180 170 160 150 140 130 120 110 100 90 80 70 60 50 40 30 20 10 0
f1 (ppm)

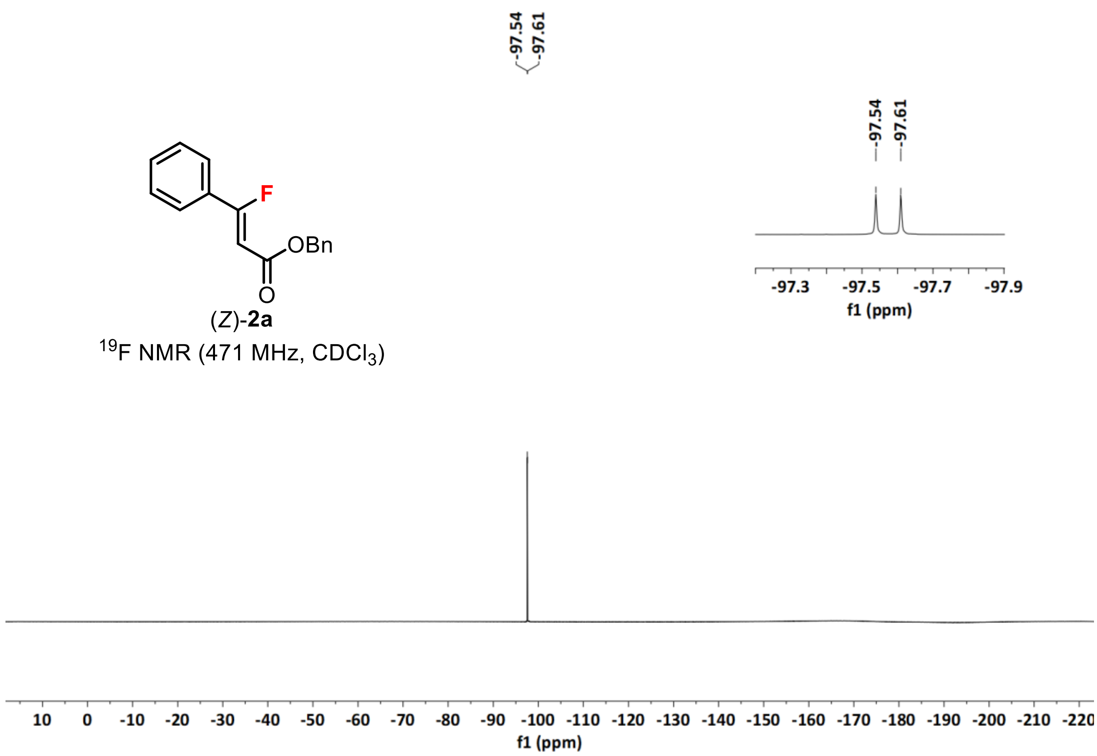
-97.54
-97.61
F
OBn
O
(Z)-2a
19F NMR (471 MHz, CDCl3)
-97.3 -97.5 -97.7 -97.9
f1 (ppm)
10 0 -10 -20 -30 -40 -50 -60 -70 -80 -90 -100 -110 -120 -130 -140 -150 -160 -170 -180 -190 -200 -210 -220
f1 (ppm)

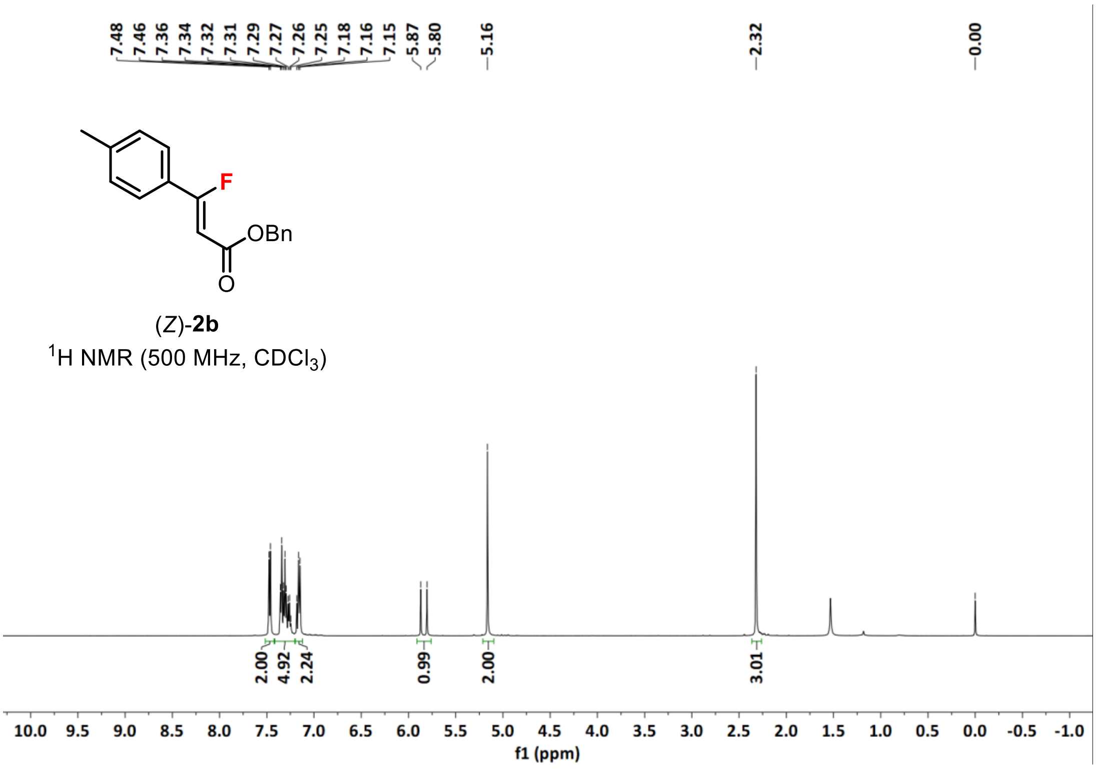
7.48 7.46 7.36 7.34 7.32 7.31 7.29 7.27 7.26 7.25 7.18 7.16 7.15 5.87 5.80 5.16 2.32 0.00
F
OBn
O
(Z)-2b
1H NMR (500 MHz, CDCl3)
2.00 4.92 2.24 0.99 2.00 3.01
10.0 9.5 9.0 8.5 8.0 7.5 7.0 6.5 6.0 5.5 5.0 4.5 4.0 3.5 3.0 2.5 2.0 1.5 1.0 0.5 0.0 -0.5 -1.0
f1 (ppm)

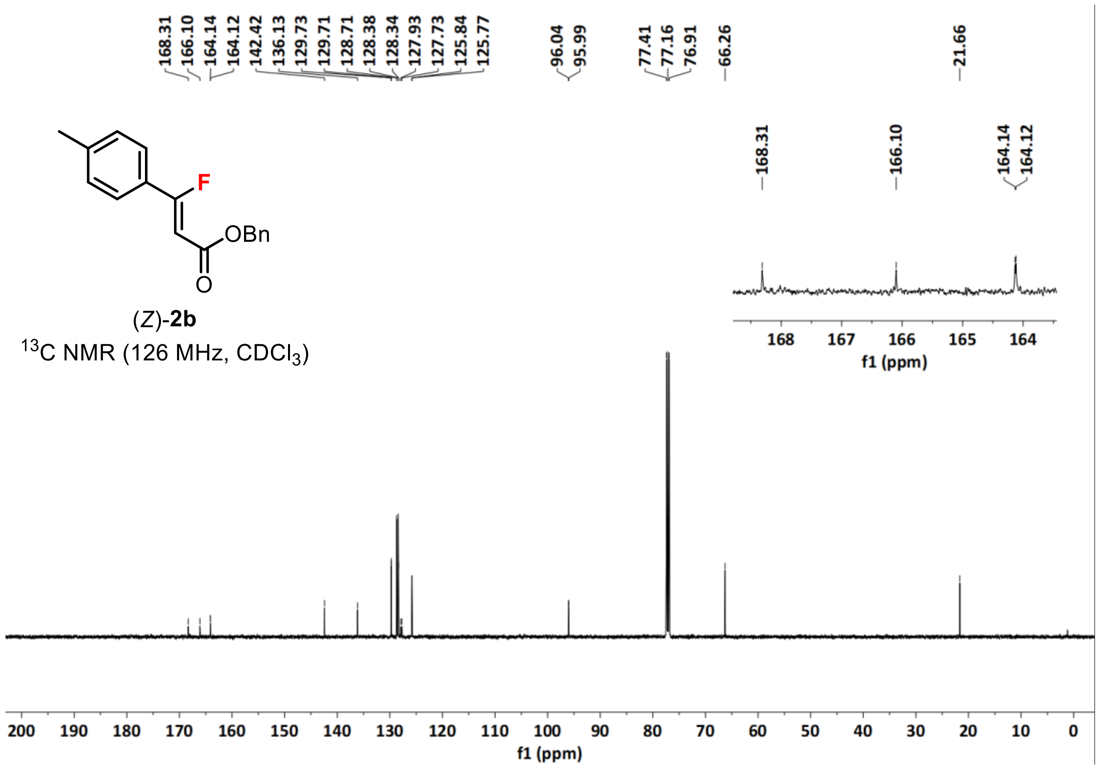
168.31
166.10
164.14
164.12
142.42
136.13
129.73
129.71
128.71
128.38
128.34
127.93
127.73
125.84
125.77
96.04
95.99
77.41
77.16
76.91
66.26
21.66
F
OBn
O
(Z)-2b
13C NMR (126 MHz, CDCl3)
f1 (ppm)

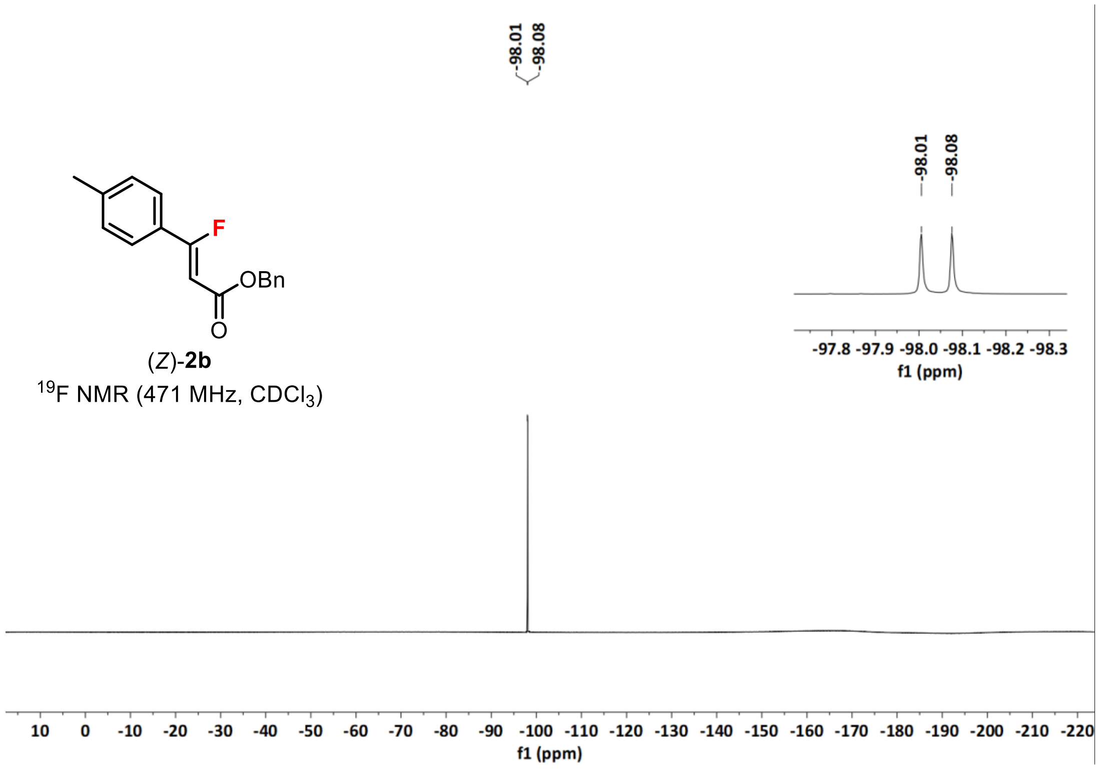
-98.01
-98.08
F
OBn
O
(Z)-2b
19F NMR (471 MHz, CDCl3)
f1 (ppm)

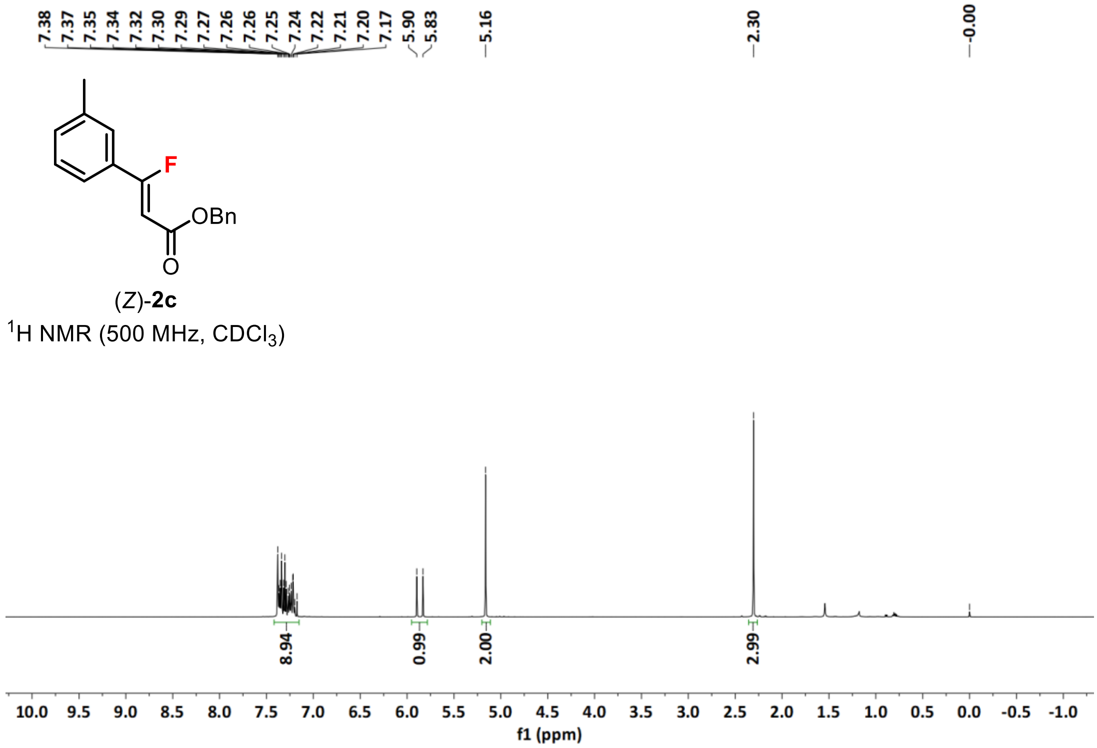
F
OBn
O
(Z)-2c
1H NMR (500 MHz, CDCl3)
f1 (ppm)

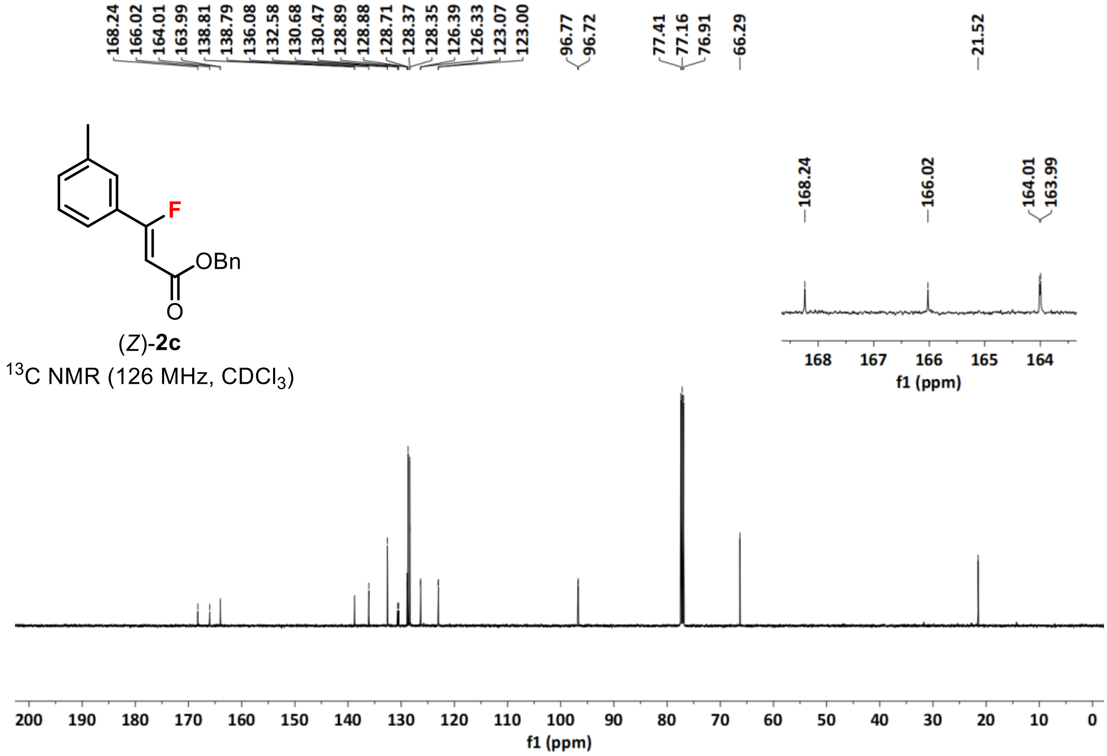
F
OBn
O
(Z)-2c
13C NMR (126 MHz, CDCl3)
f1 (ppm)

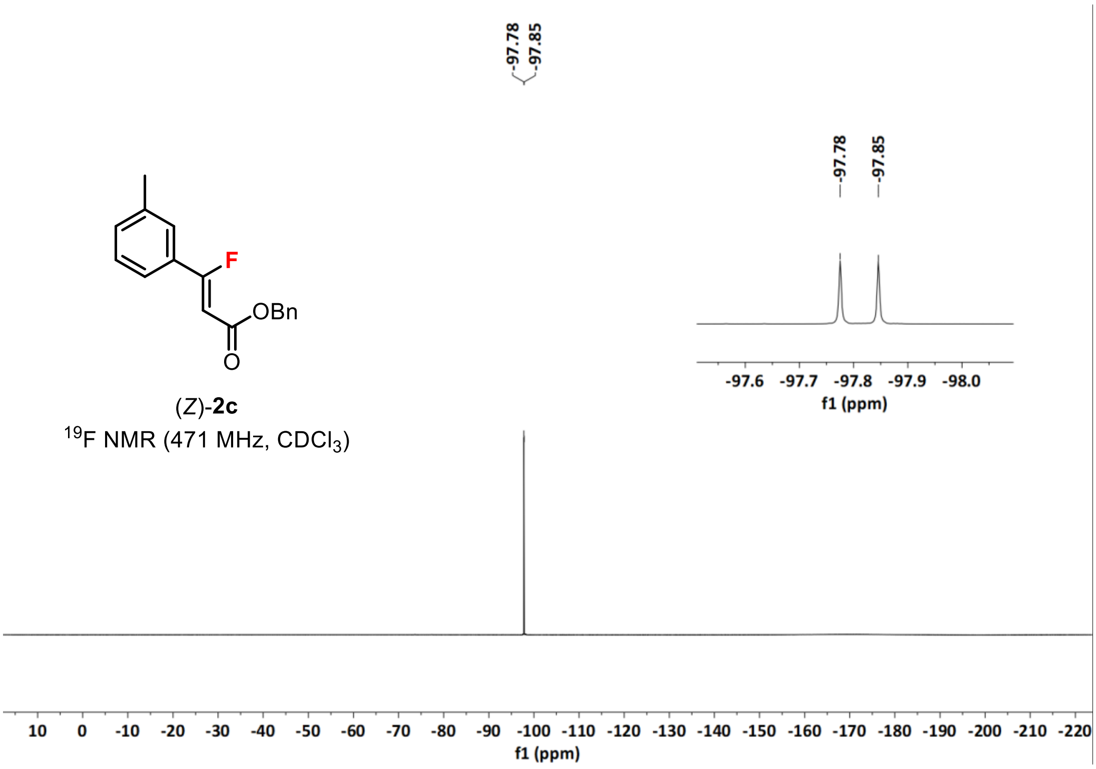

(*Z*)-**2c**
^{19}F NMR (471 MHz, $CDCl_3$)

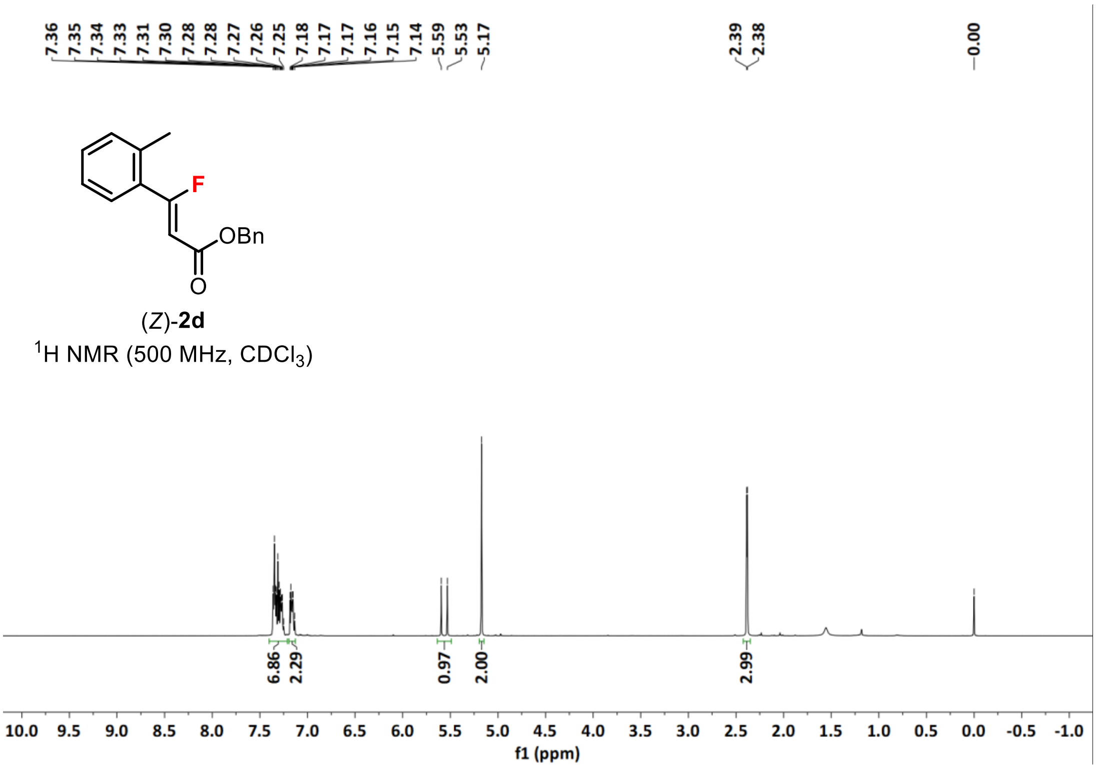

(*Z*)-**2d**
^{1}H NMR (500 MHz, $CDCl_3$)

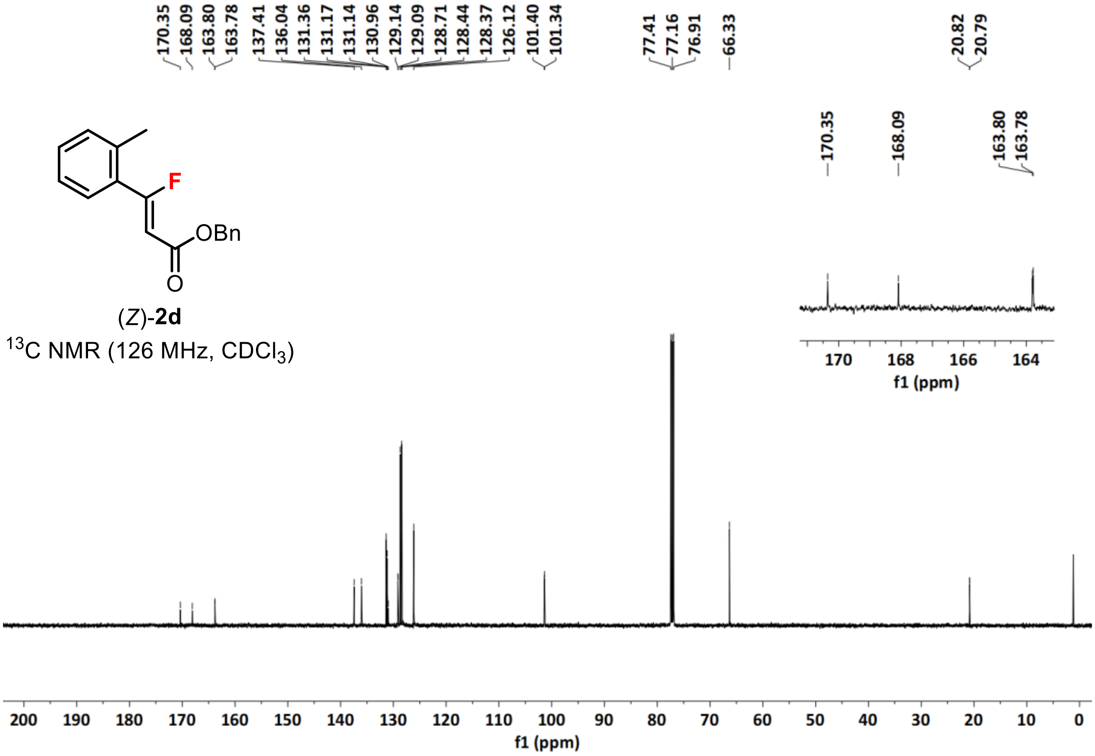

(*Z*)-**2d**

^{13}C NMR (126 MHz, $CDCl_3$)

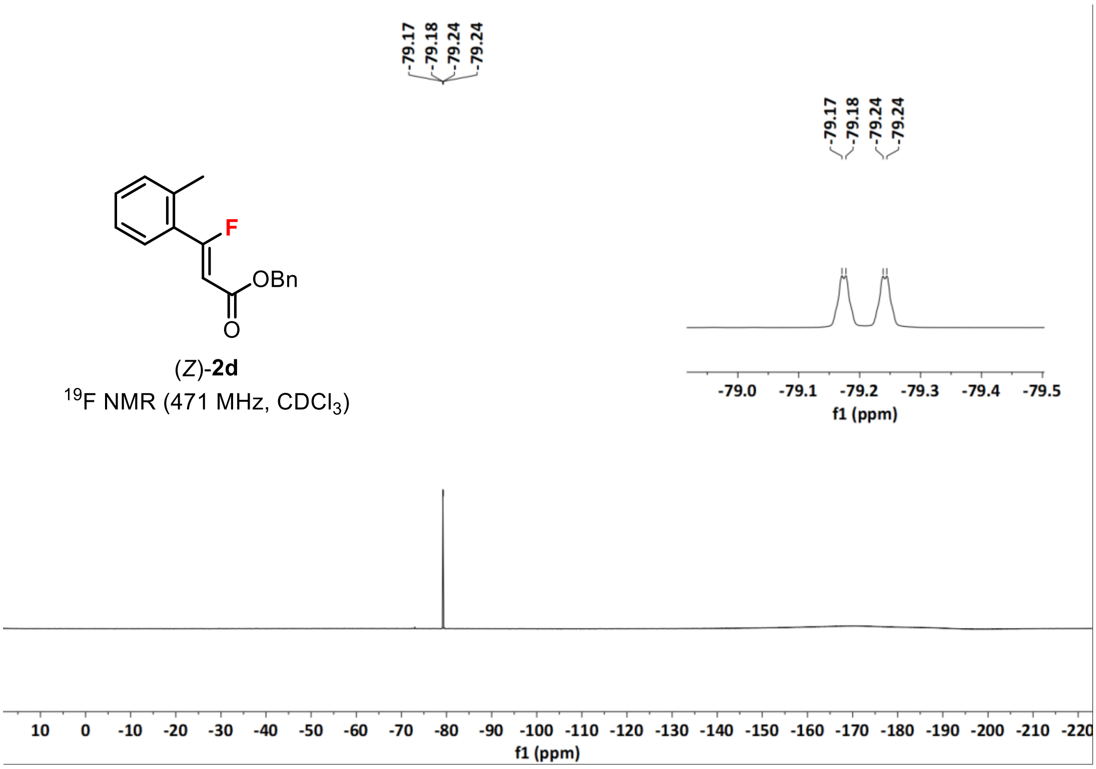

(*Z*)-**2d**

^{19}F NMR (471 MHz, $CDCl_3$)

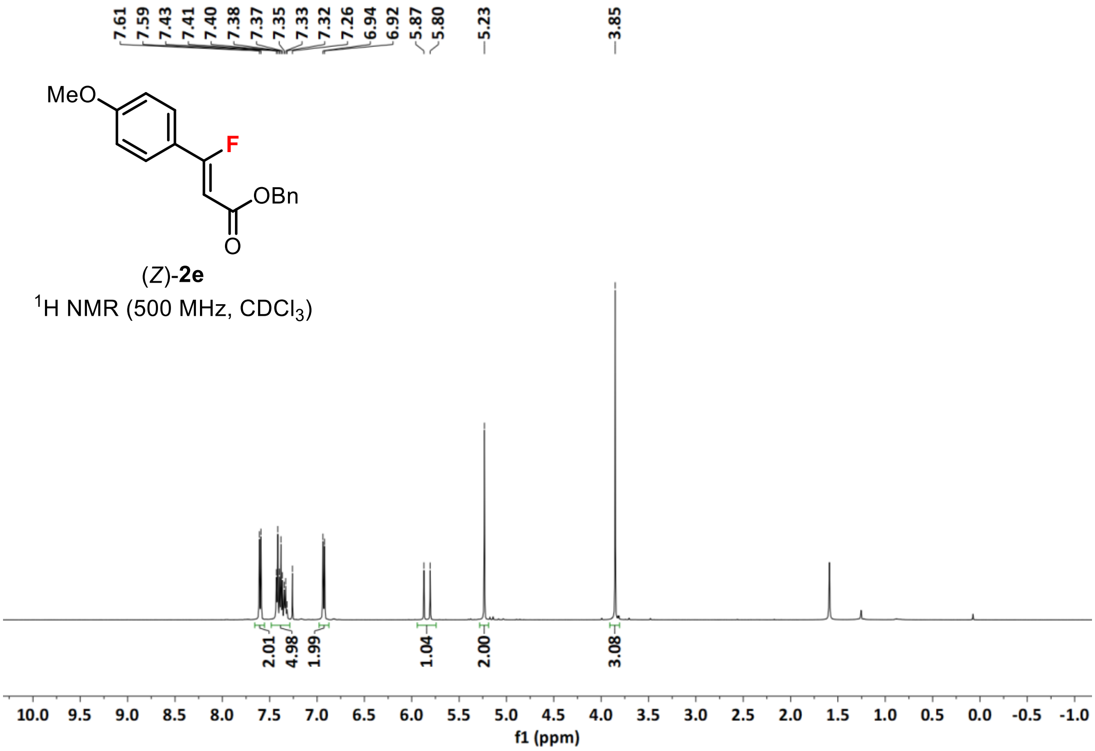
7.61 7.59 7.43 7.41 7.40 7.38 7.37 7.35 7.33 7.32 7.26 6.94 6.92 5.87 5.80 5.23 3.85
MeO
F
OBn
O
(Z)-2e
1H NMR (500 MHz, CDCl3)
2.01 4.98 1.99 1.04 2.00 3.08
10.0 9.5 9.0 8.5 8.0 7.5 7.0 6.5 6.0 5.5 5.0 4.5 4.0 3.5 3.0 2.5 2.0 1.5 1.0 0.5 0.0 -0.5 -1.0
f1 (ppm)

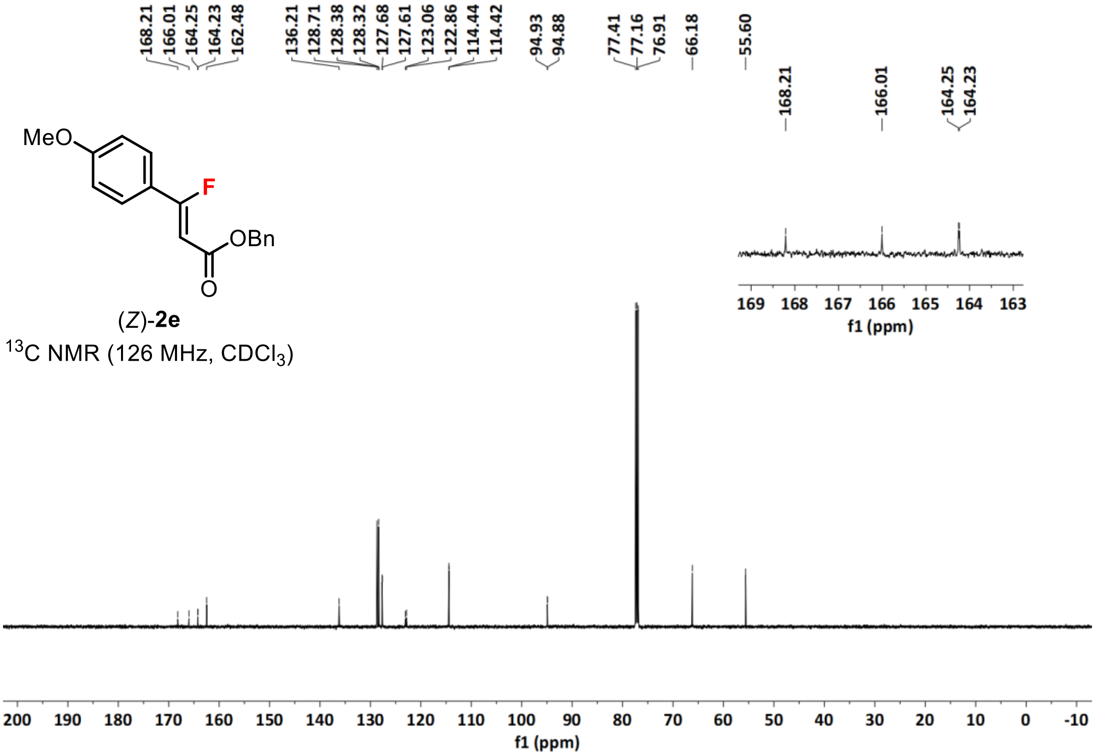
168.21 166.01 164.25 164.23 162.48 136.21 128.71 128.38 128.32 127.68 127.61 123.06 122.86 114.44 114.42 94.93 94.88 77.41 77.16 76.91 66.18 55.60
MeO
F
OBn
O
(Z)-2e
13C NMR (126 MHz, CDCl3)
168.21 166.01 164.25 164.23
169 168 167 166 165 164 163
f1 (ppm)
200 190 180 170 160 150 140 130 120 110 100 90 80 70 60 50 40 30 20 10 0 -10
f1 (ppm)

(*Z*)-**2e**

^{19}F NMR (471 MHz, $CDCl_3$)

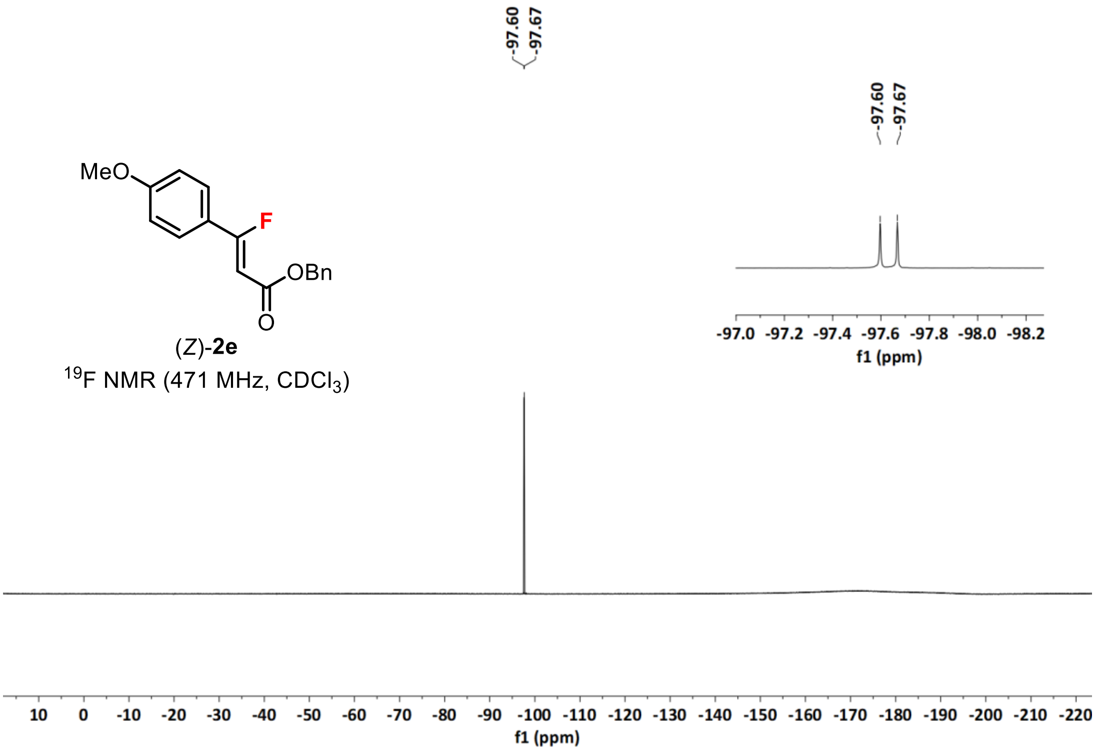

(*Z*)-**2f**

^{1}H NMR (500 MHz, $CDCl_3$)

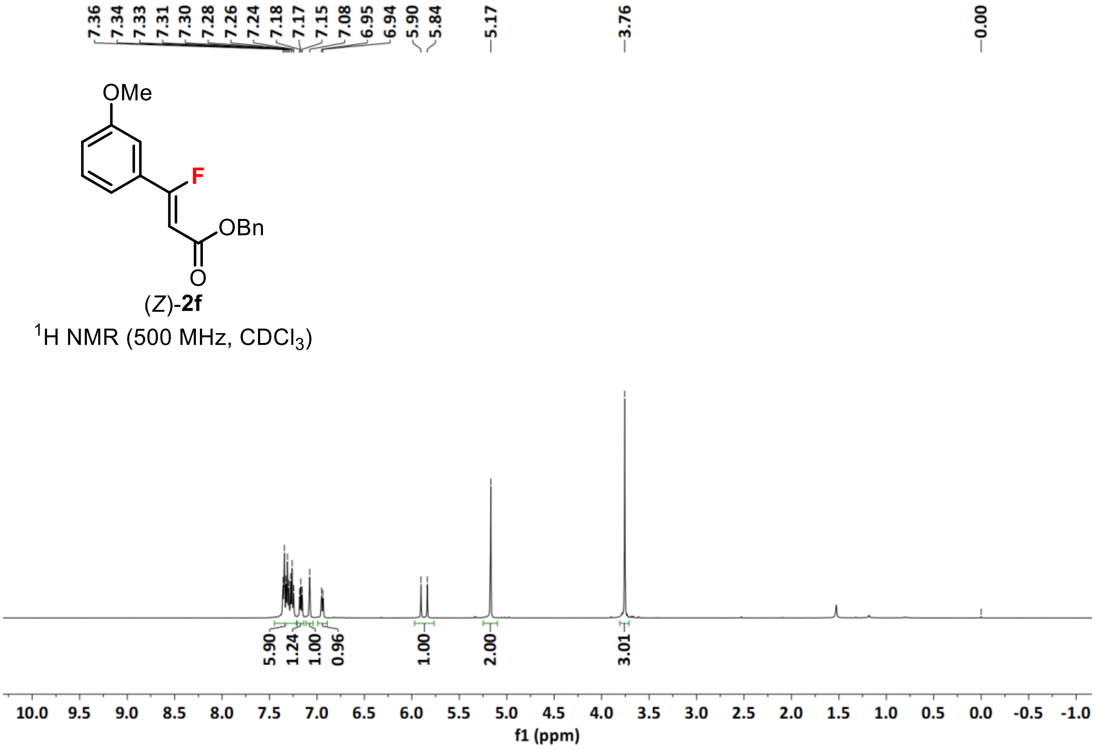

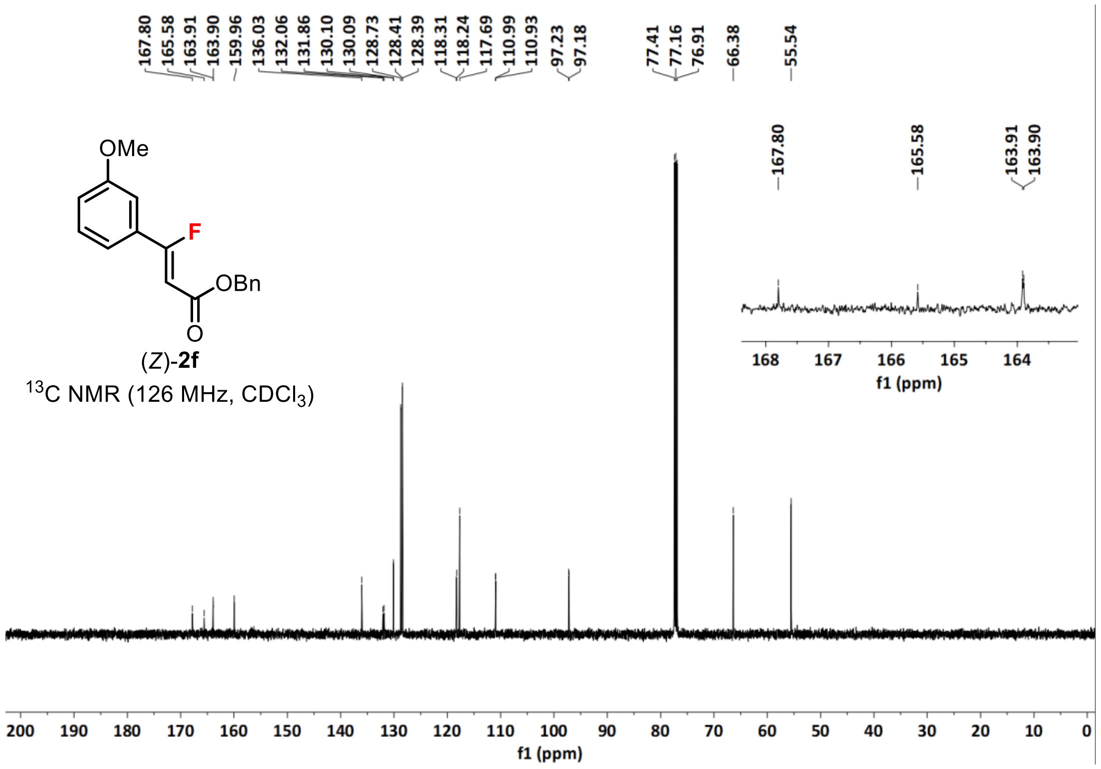
167.80
165.58
163.91
163.90
159.96
136.03
132.06
131.86
130.10
130.09
128.73
128.41
128.39
118.31
118.24
117.69
110.99
110.93
97.23
97.18
77.41
77.16
76.91
66.38
55.54
OMe
F
OBn
O
(Z)-2f
13C NMR (126 MHz, CDCl3)
f1 (ppm)

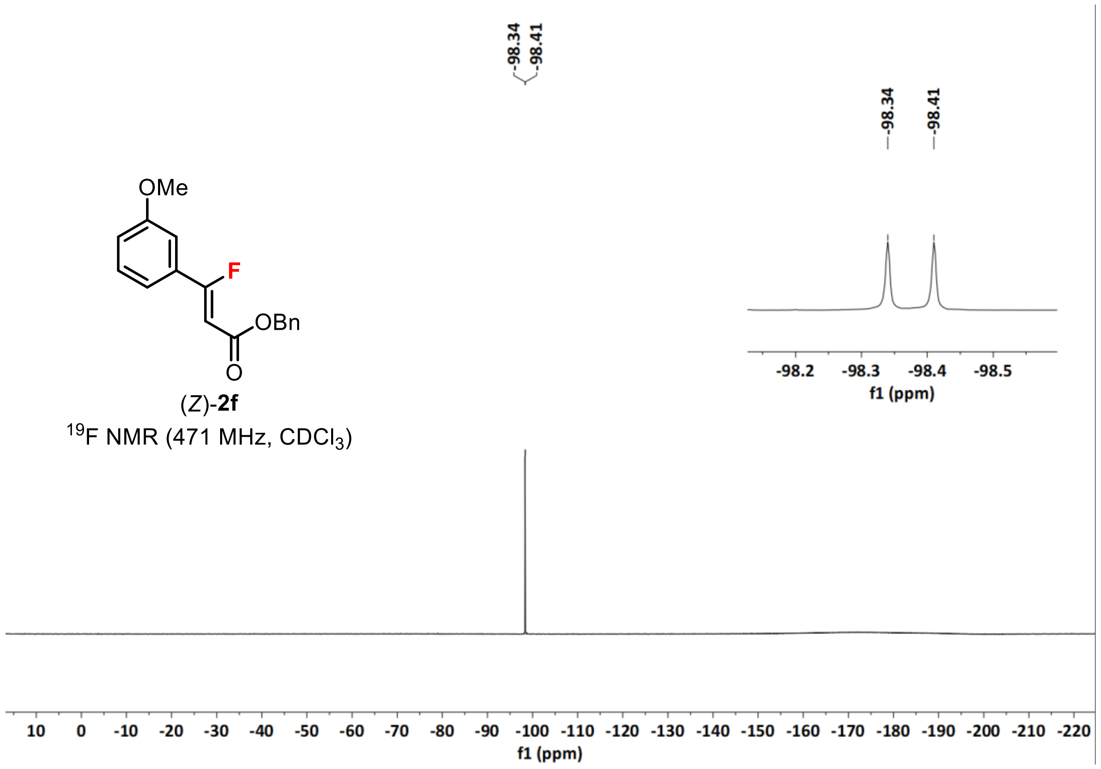
-98.34
-98.41
OMe
F
OBn
O
(Z)-2f
19F NMR (471 MHz, CDCl3)
f1 (ppm)

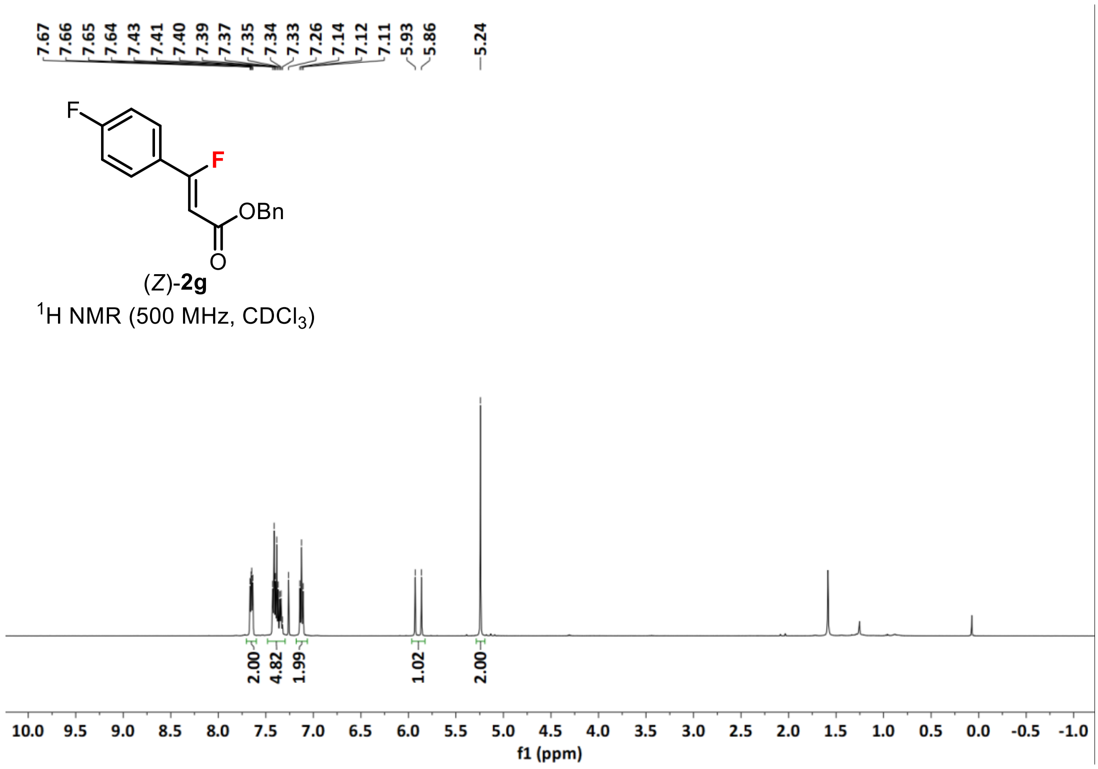
7.67 7.66 7.65 7.64 7.43 7.41 7.40 7.39 7.37 7.35 7.34 7.33 7.26 7.14 7.12 7.11 5.93 5.86 5.24
F
F
OBn
O
(Z)-2g
1H NMR (500 MHz, CDCl3)
2.00 4.82 1.99 1.02 2.00
10.0 9.5 9.0 8.5 8.0 7.5 7.0 6.5 6.0 5.5 5.0 4.5 4.0 3.5 3.0 2.5 2.0 1.5 1.0 0.5 0.0 -0.5 -1.0
f1 (ppm)

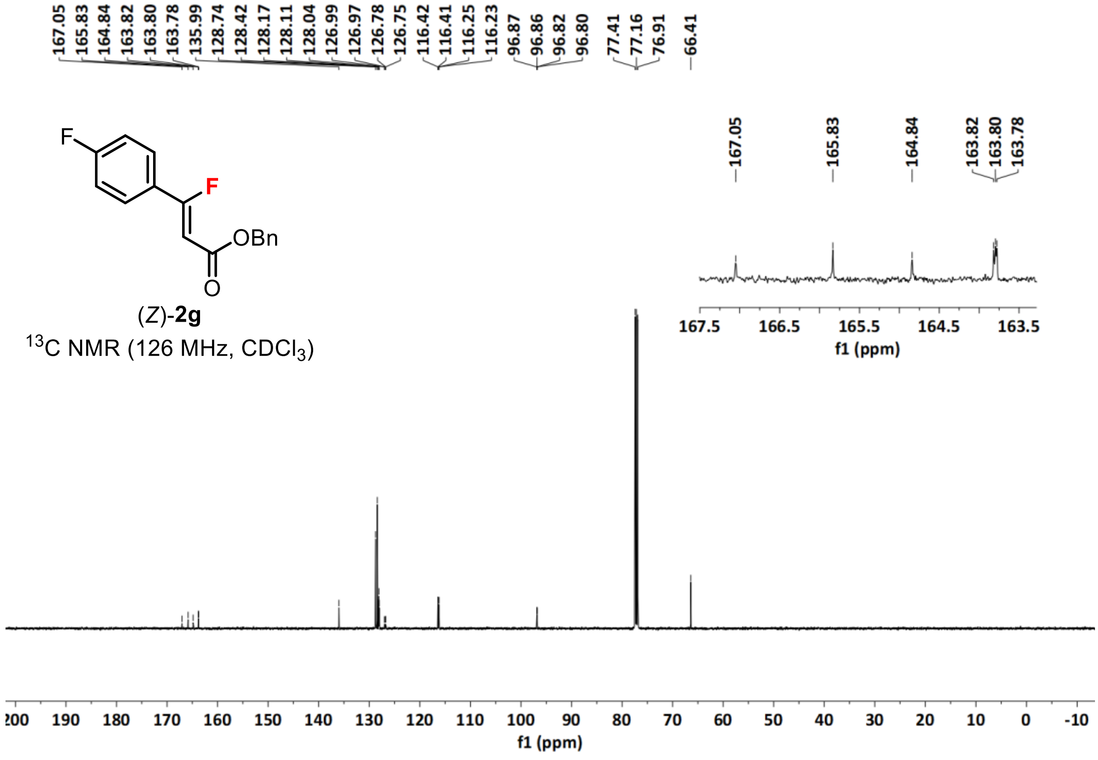
167.05 165.83 164.84 163.82 163.80 163.78 135.99 128.74 128.42 128.17 128.11 128.04 126.99 126.97 126.78 126.75 116.42 116.41 116.25 116.23 96.87 96.86 96.82 96.80 77.41 77.16 76.91 66.41
F
F
OBn
O
(Z)-2g
13C NMR (126 MHz, CDCl3)
167.05 165.83 164.84 163.82 163.80 163.78
167.5 166.5 165.5 164.5 163.5
f1 (ppm)
200 190 180 170 160 150 140 130 120 110 100 90 80 70 60 50 40 30 20 10 0 -10
f1 (ppm)

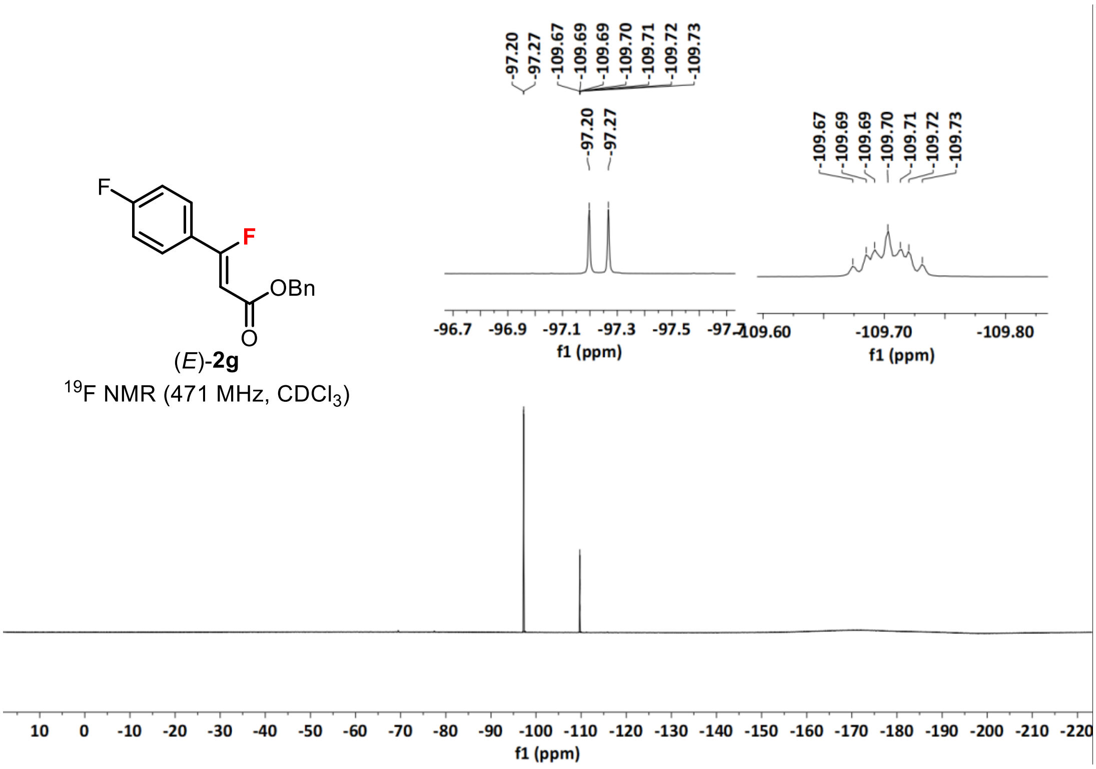

(*E*)-**2g**

^{19}F NMR (471 MHz, $CDCl_3$)

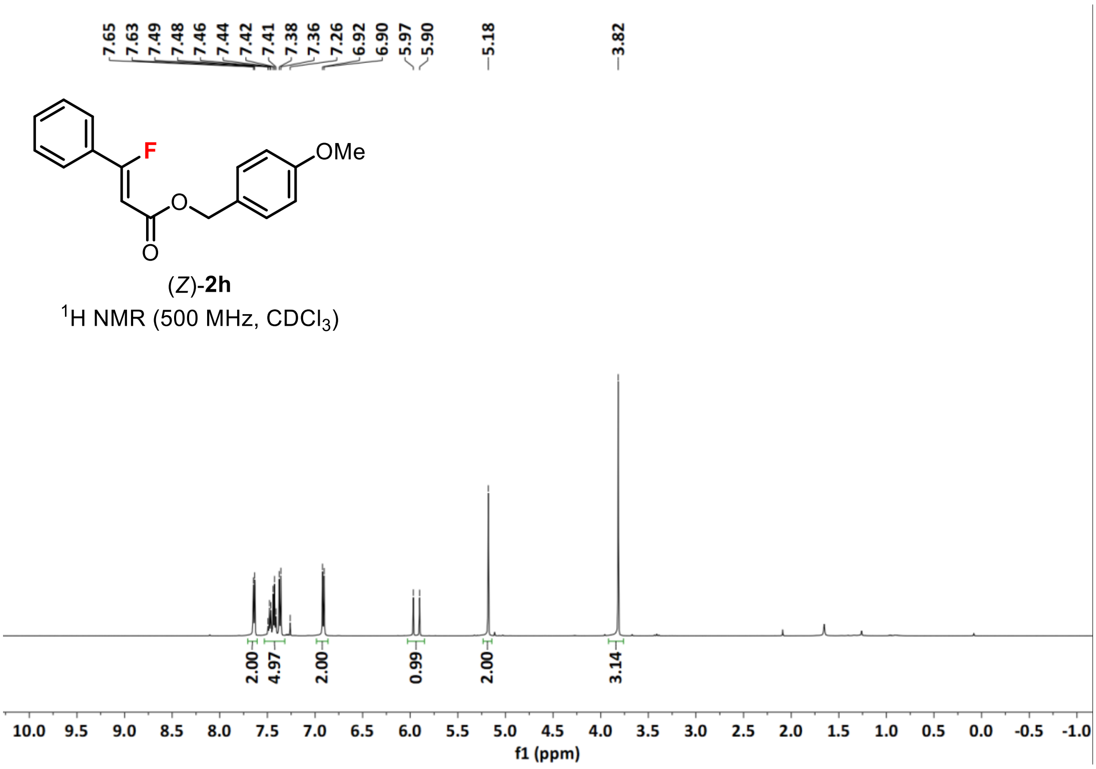

(*Z*)-**2h**

^{1}H NMR (500 MHz, $CDCl_3$)

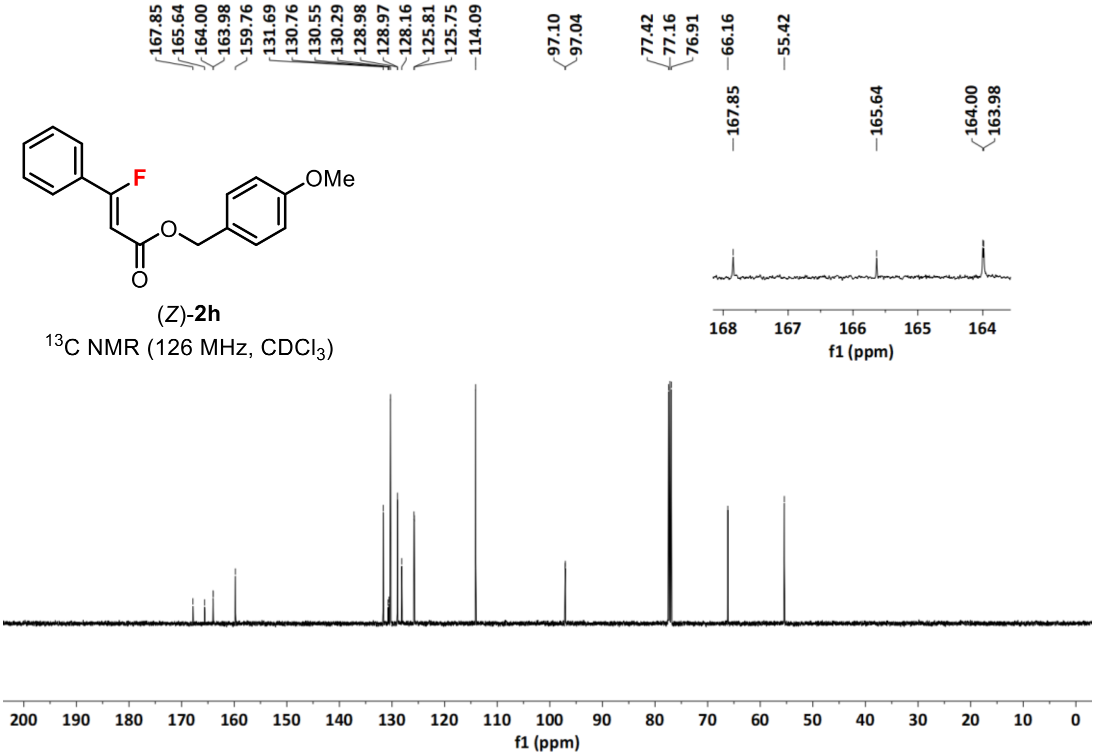

(*Z*)-**2h**

^{13}C NMR (126 MHz, $CDCl_3$)

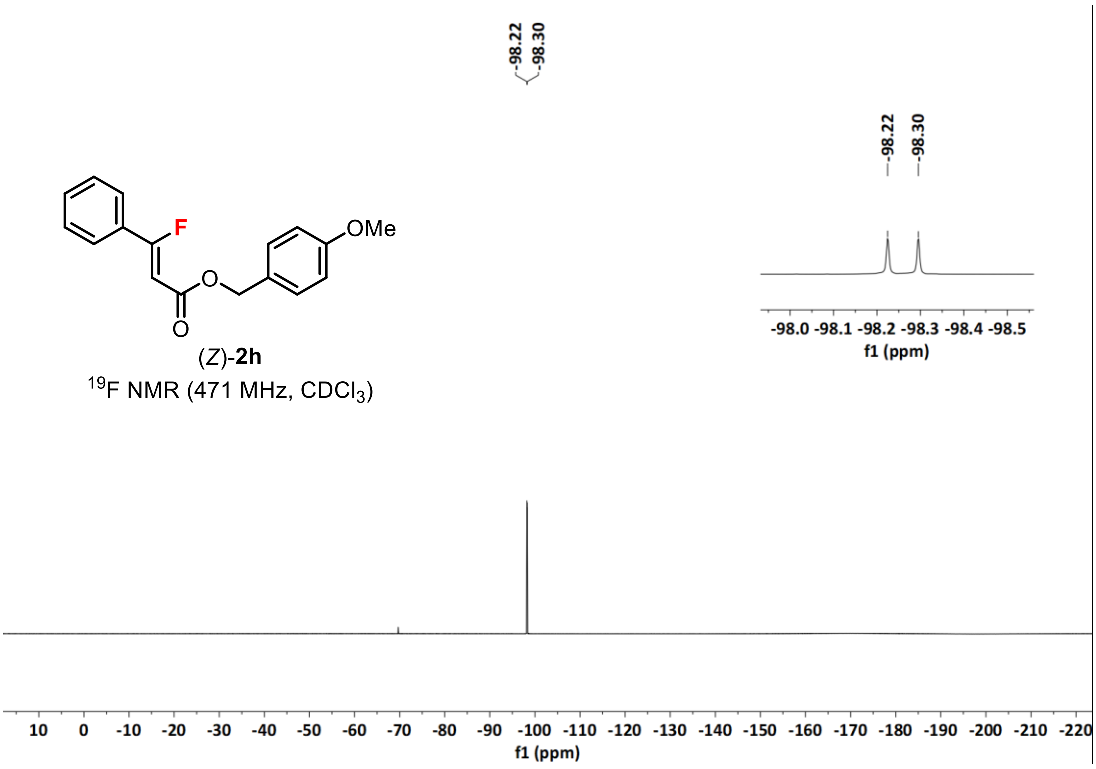

(*Z*)-**2h**

^{19}F NMR (471 MHz, $CDCl_3$)

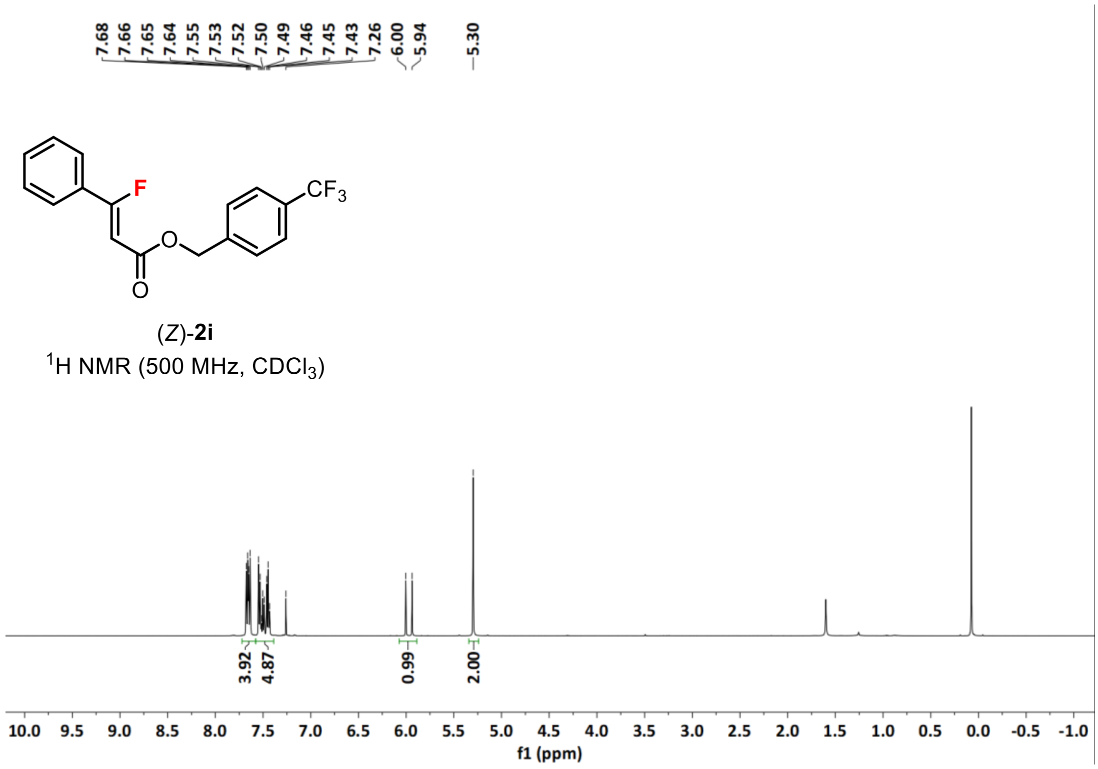
7.68
7.66
7.65
7.64
7.55
7.53
7.52
7.50
7.49
7.46
7.45
7.43
7.26
6.00
5.94
5.30
CF3
F
O
O
(Z)-2i
1H NMR (500 MHz, CDCl3)
3.92
4.87
0.99
2.00
f1 (ppm)

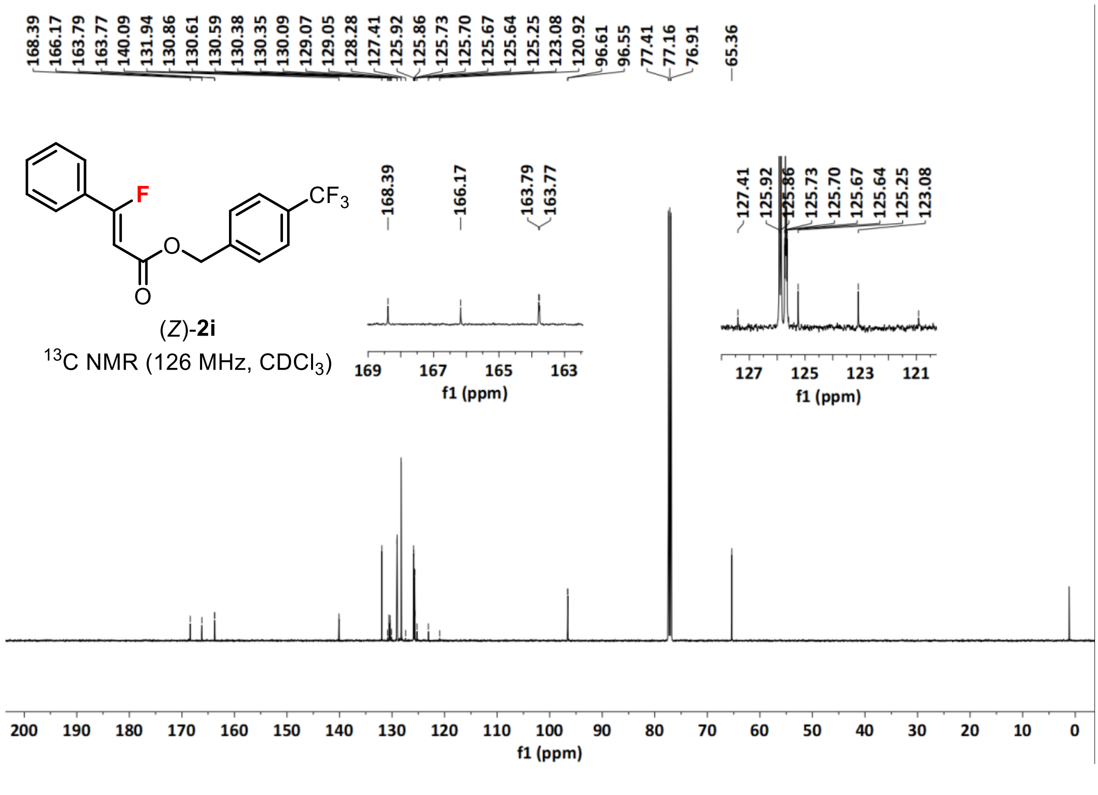
168.39
166.17
163.79
163.77
140.09
131.94
130.86
130.61
130.59
130.38
130.35
130.09
129.07
129.05
128.28
127.41
125.92
125.86
125.73
125.70
125.67
125.64
125.25
123.08
120.92
96.61
96.55
77.41
77.16
76.91
65.36
CF3
F
O
O
(Z)-2i
13C NMR (126 MHz, CDCl3)
f1 (ppm)

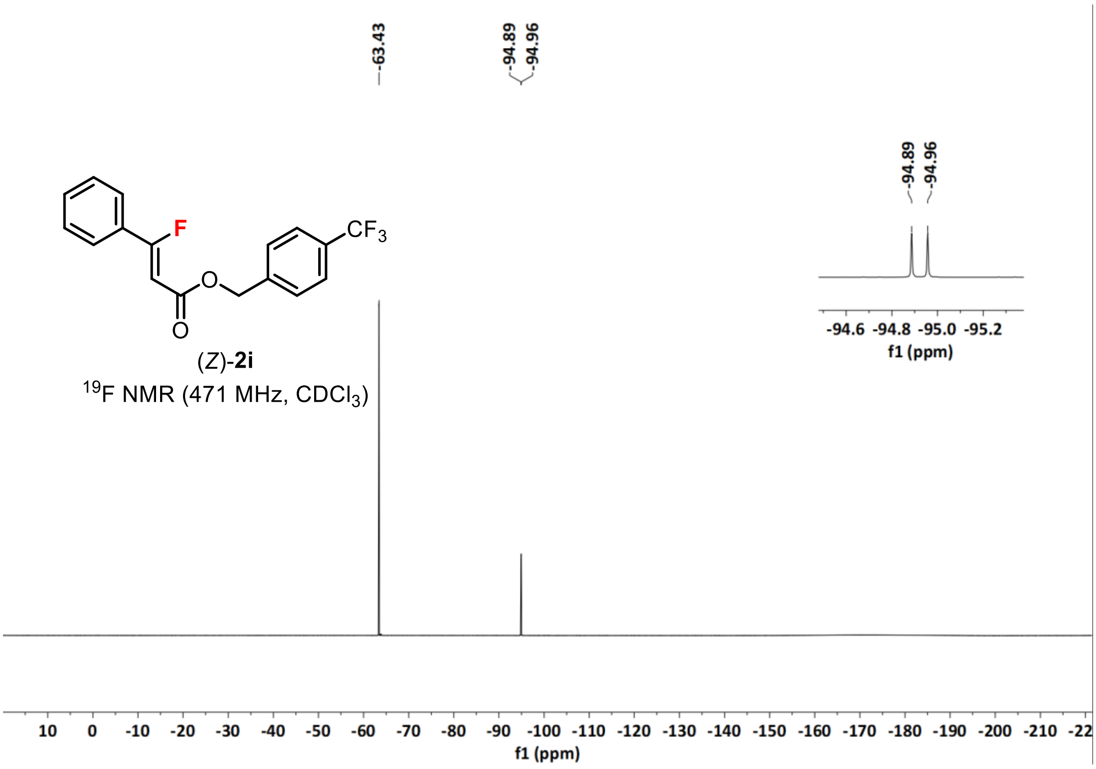

(Z)-2i
19F NMR (471 MHz, CDCl3)
CF3
F
O
-63.43
-94.89
-94.96
f1 (ppm)

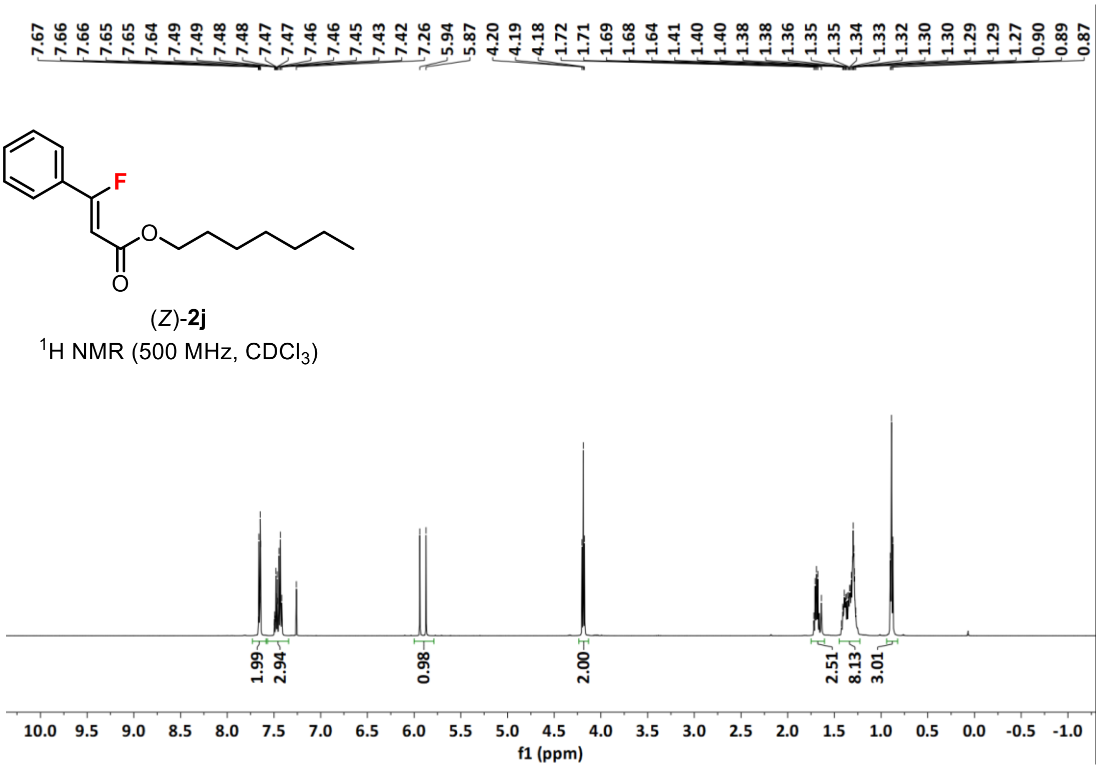

(Z)-2j
1H NMR (500 MHz, CDCl3)
F
O
7.67 7.66 7.66 7.65 7.65 7.64 7.49 7.49 7.48 7.48 7.47 7.47 7.46 7.46 7.45 7.43 7.42 7.26 5.94 5.87 4.20 4.19 4.18 1.72 1.71 1.69 1.68 1.64 1.41 1.40 1.40 1.38 1.38 1.36 1.35 1.35 1.34 1.33 1.32 1.30 1.30 1.29 1.29 1.27 0.90 0.89 0.87
1.99 2.94 0.98 2.00 2.51 8.13 3.01
f1 (ppm)

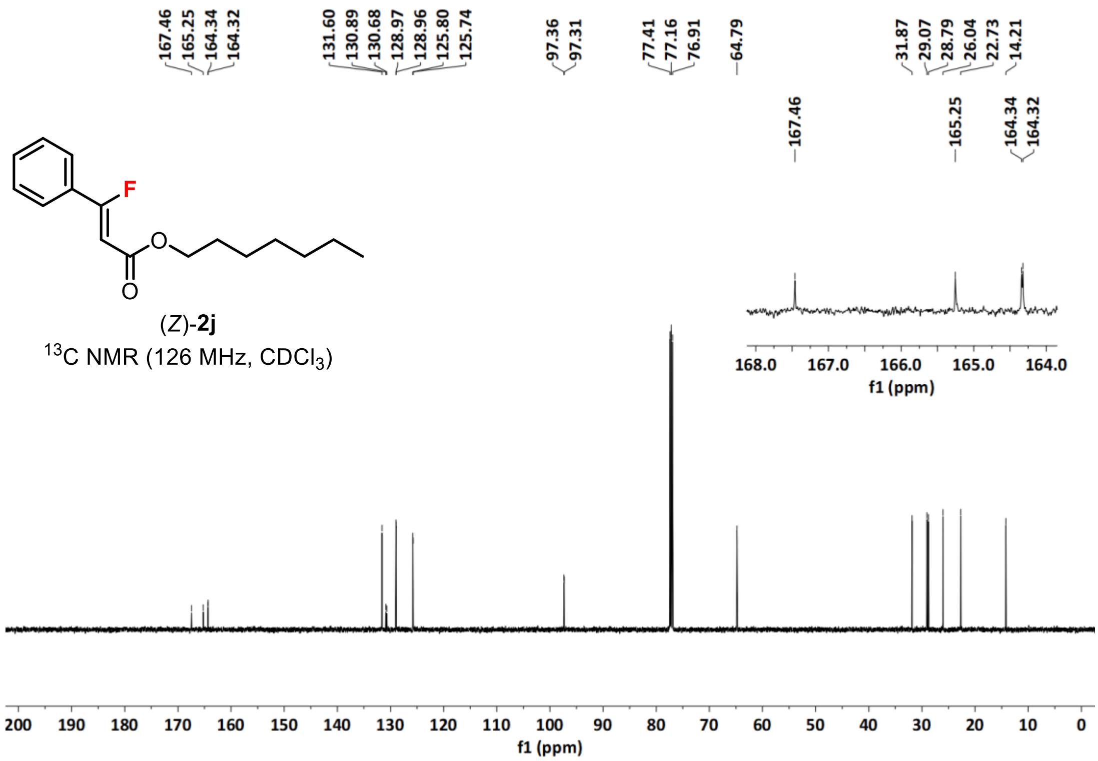
(Z)-2j
^{13}C NMR (126 MHz, $CDCl_3$)
f1 (ppm)

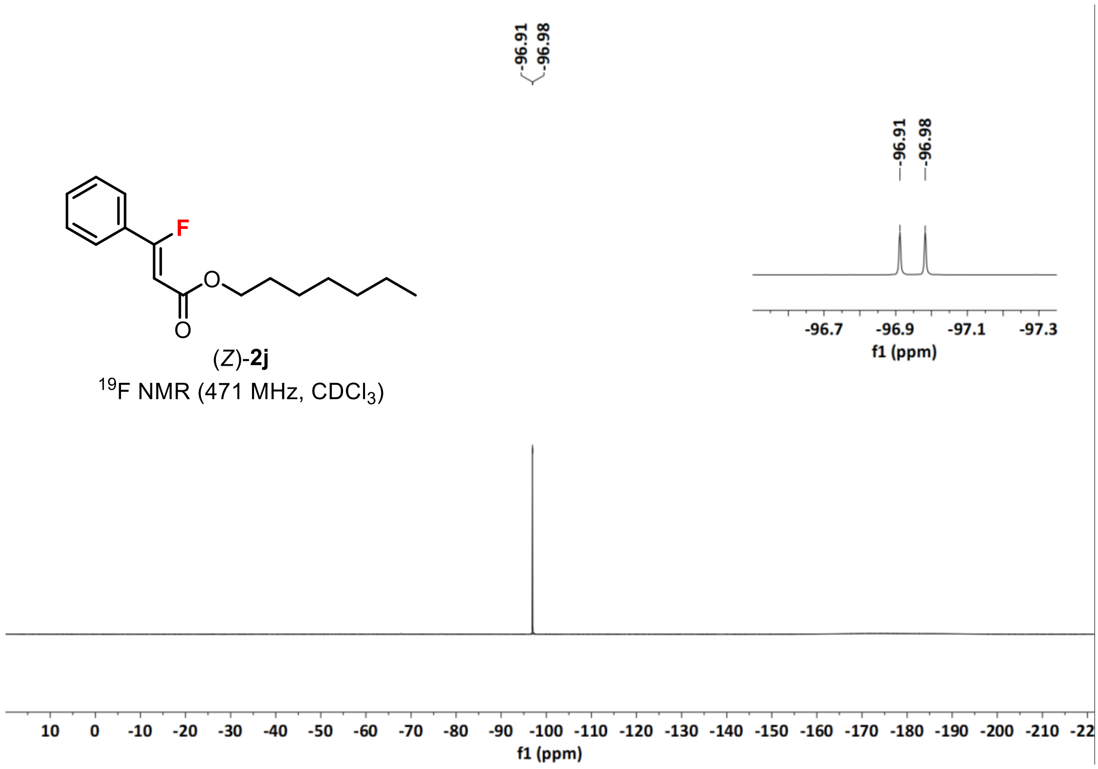
(Z)-2j
^{19}F NMR (471 MHz, $CDCl_3$)
f1 (ppm)

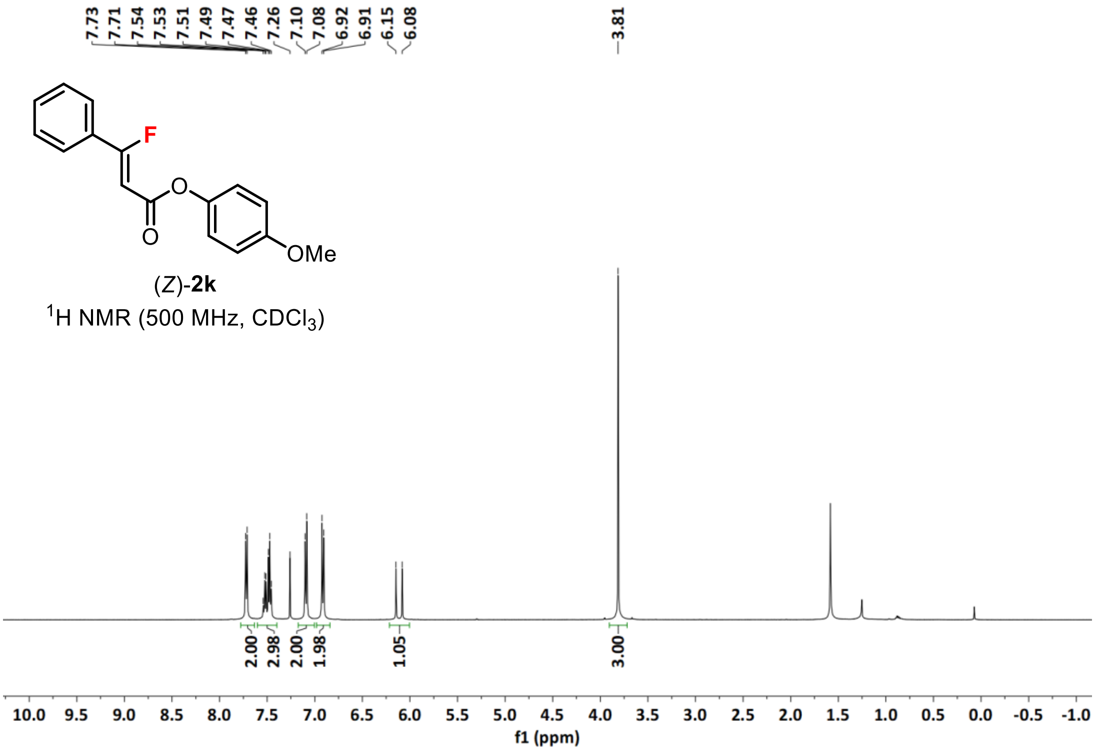

(*Z*)-**2k**

^{1}H NMR (500 MHz, $CDCl_3$)

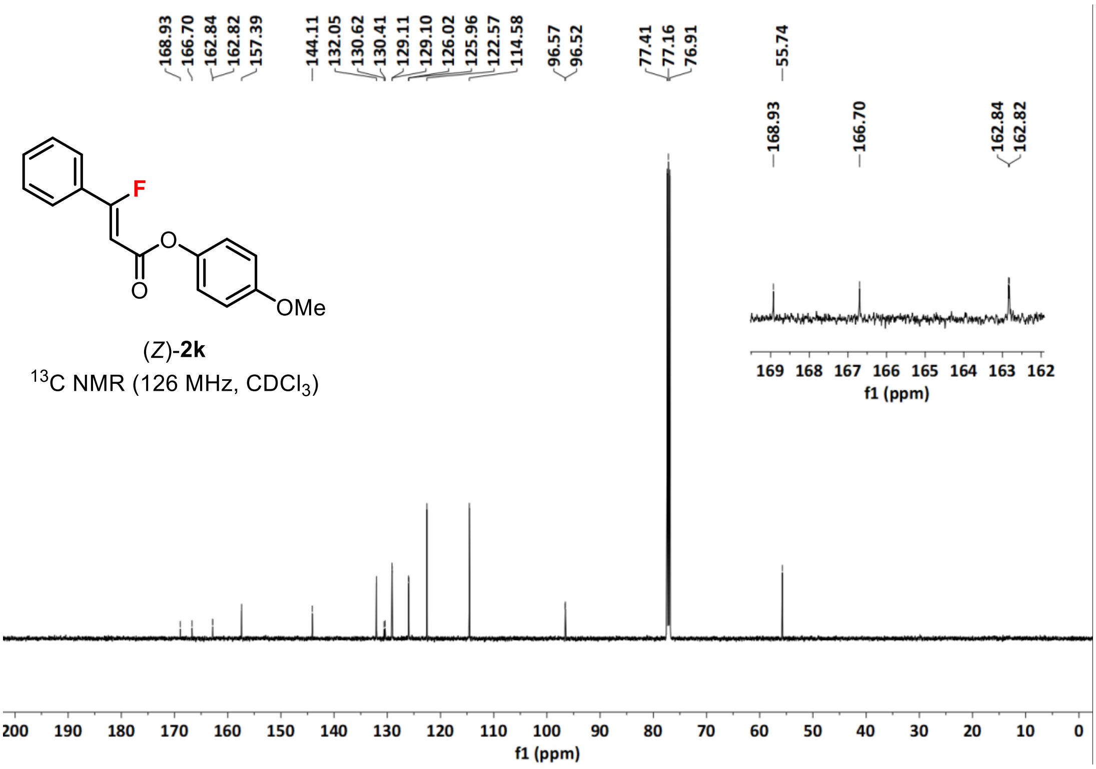

(*Z*)-**2k**

^{13}C NMR (126 MHz, $CDCl_3$)

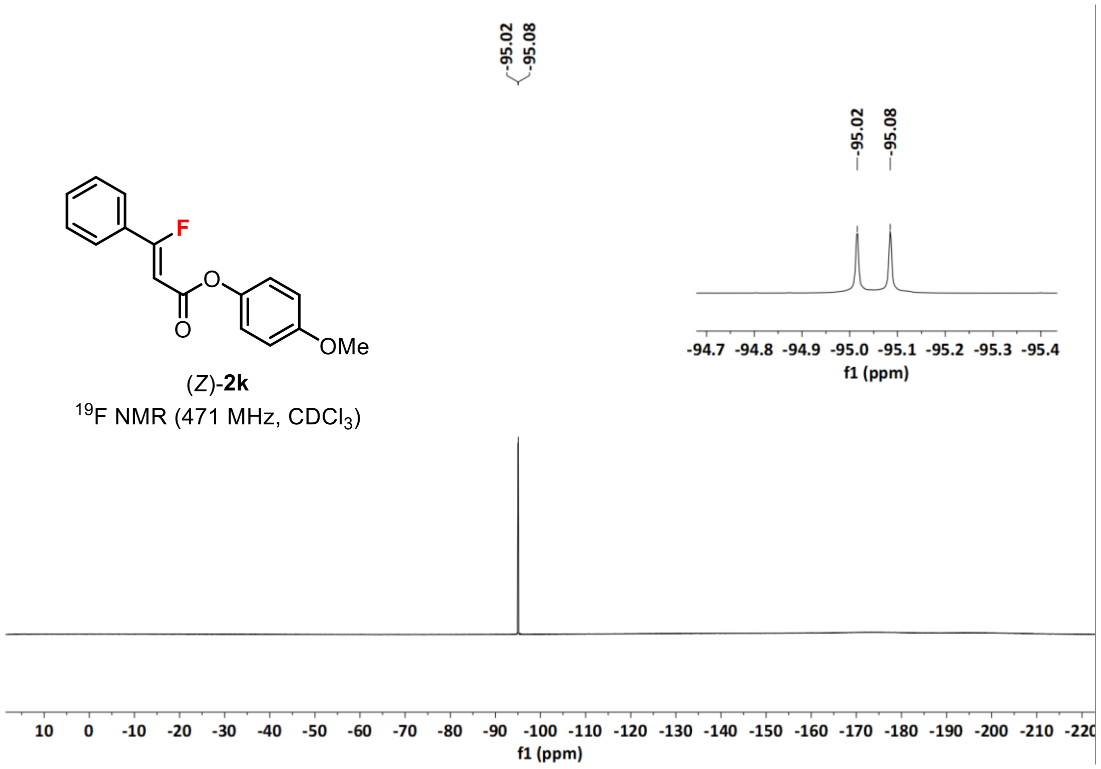

(*Z*)-**2k**

^{19}F NMR (471 MHz, $CDCl_3$)

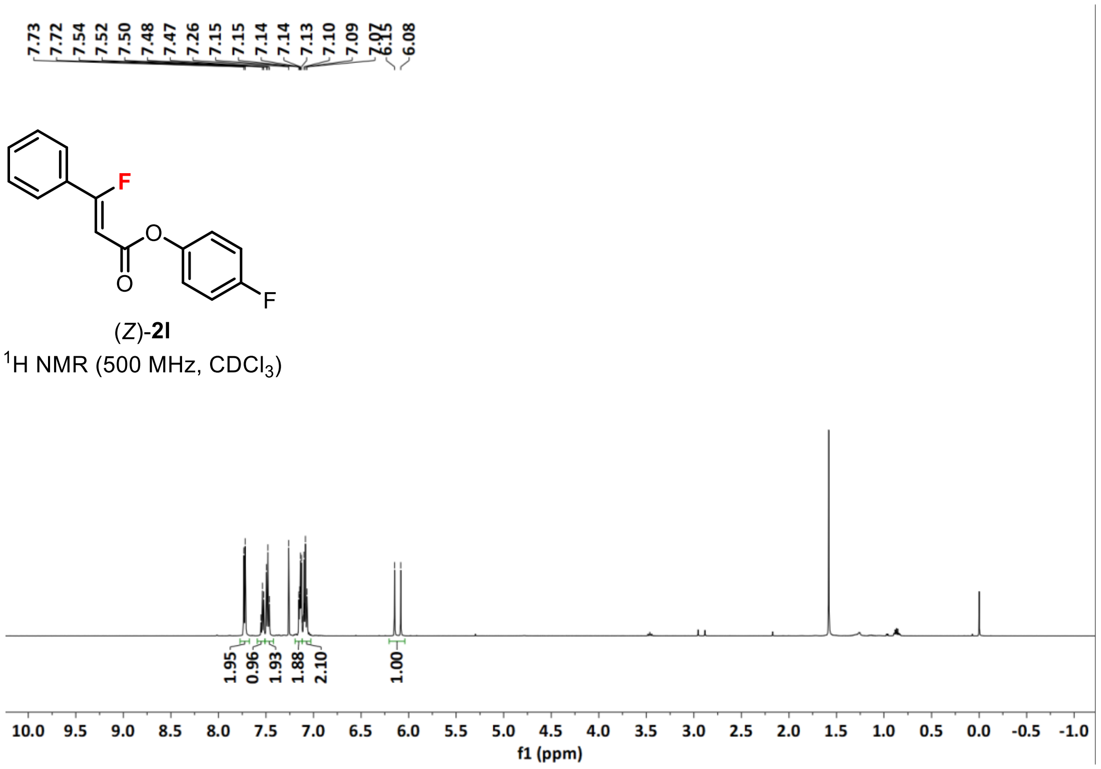

(*Z*)-**2l**

^{1}H NMR (500 MHz, $CDCl_3$)

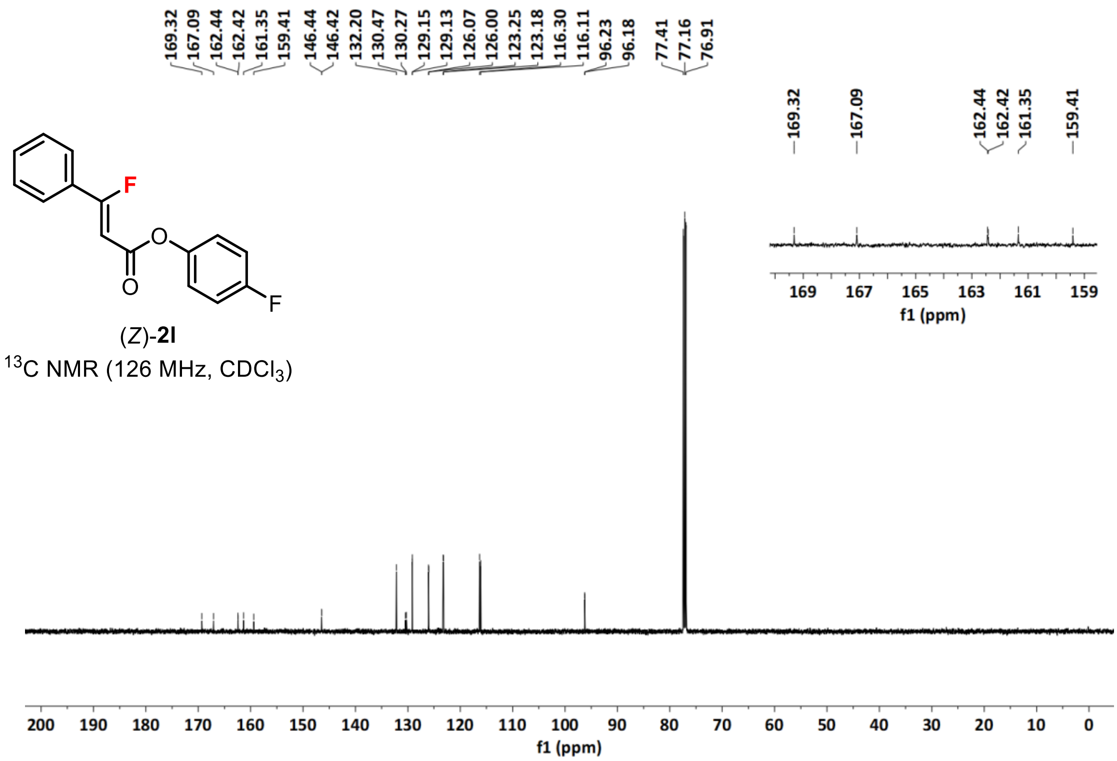

(*Z*)-**2l**

^{13}C NMR (126 MHz, $CDCl_3$)

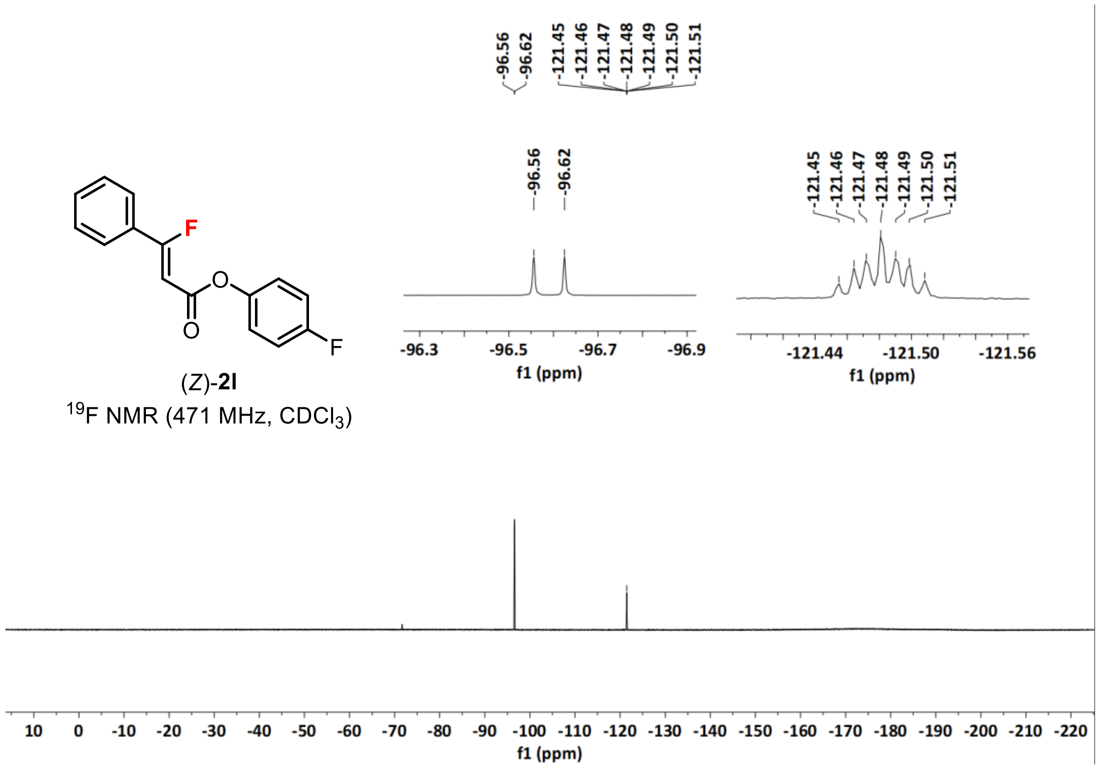

(*Z*)-**2l**

^{19}F NMR (471 MHz, $CDCl_3$)

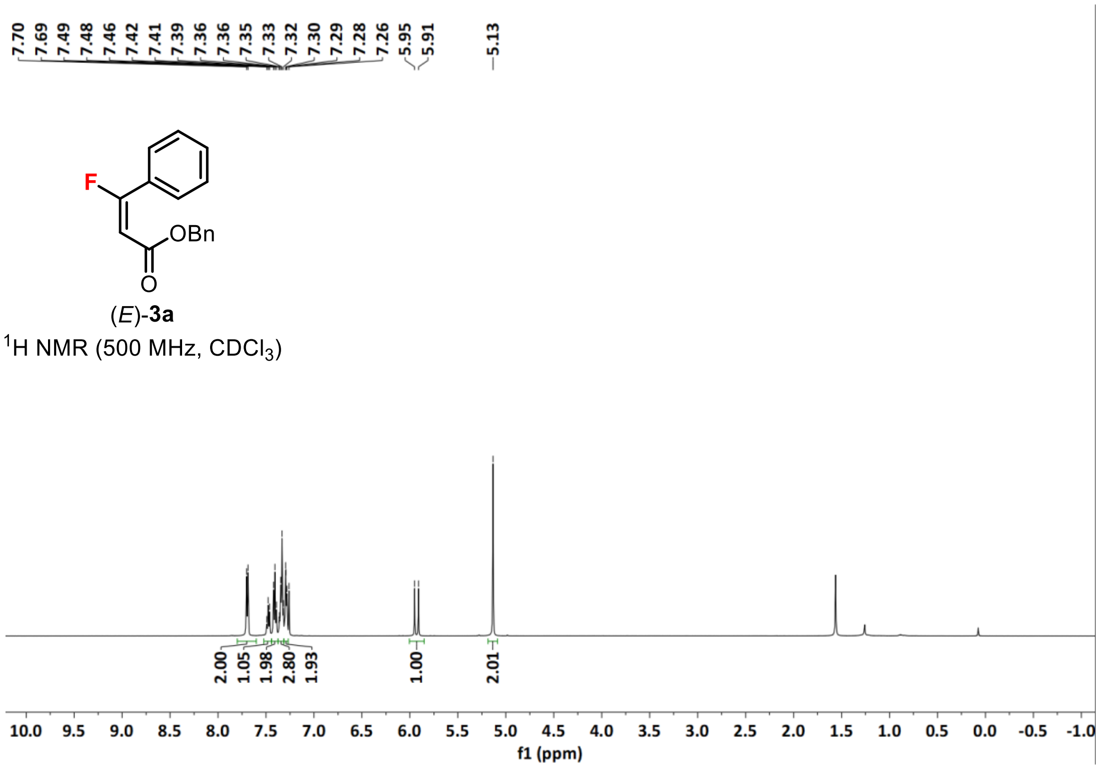
7.70
7.69
7.49
7.48
7.46
7.42
7.41
7.39
7.36
7.36
7.35
7.33
7.32
7.30
7.29
7.28
7.26
5.95
5.91
5.13
F
OBn
O
(E)-3a
1H NMR (500 MHz, CDCl3)
2.00
1.05
1.98
2.80
1.93
1.00
2.01
10.0 9.5 9.0 8.5 8.0 7.5 7.0 6.5 6.0 5.5 5.0 4.5 4.0 3.5 3.0 2.5 2.0 1.5 1.0 0.5 0.0 -0.5 -1.0
f1 (ppm)

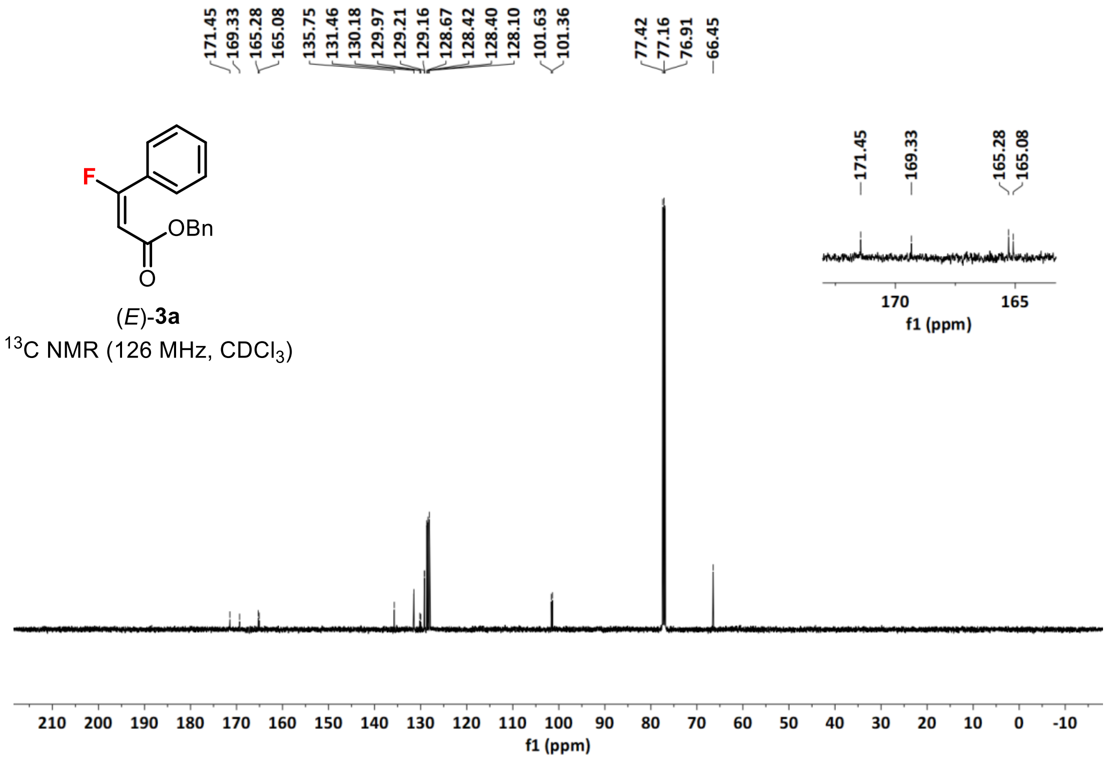
171.45
169.33
165.28
165.08
135.75
131.46
130.18
129.97
129.21
129.16
128.67
128.42
128.40
128.10
101.63
101.36
77.42
77.16
76.91
66.45
F
OBn
O
(E)-3a
13C NMR (126 MHz, CDCl3)
171.45
169.33
165.28
165.08
170
165
f1 (ppm)
210 200 190 180 170 160 150 140 130 120 110 100 90 80 70 60 50 40 30 20 10 0 -10
f1 (ppm)

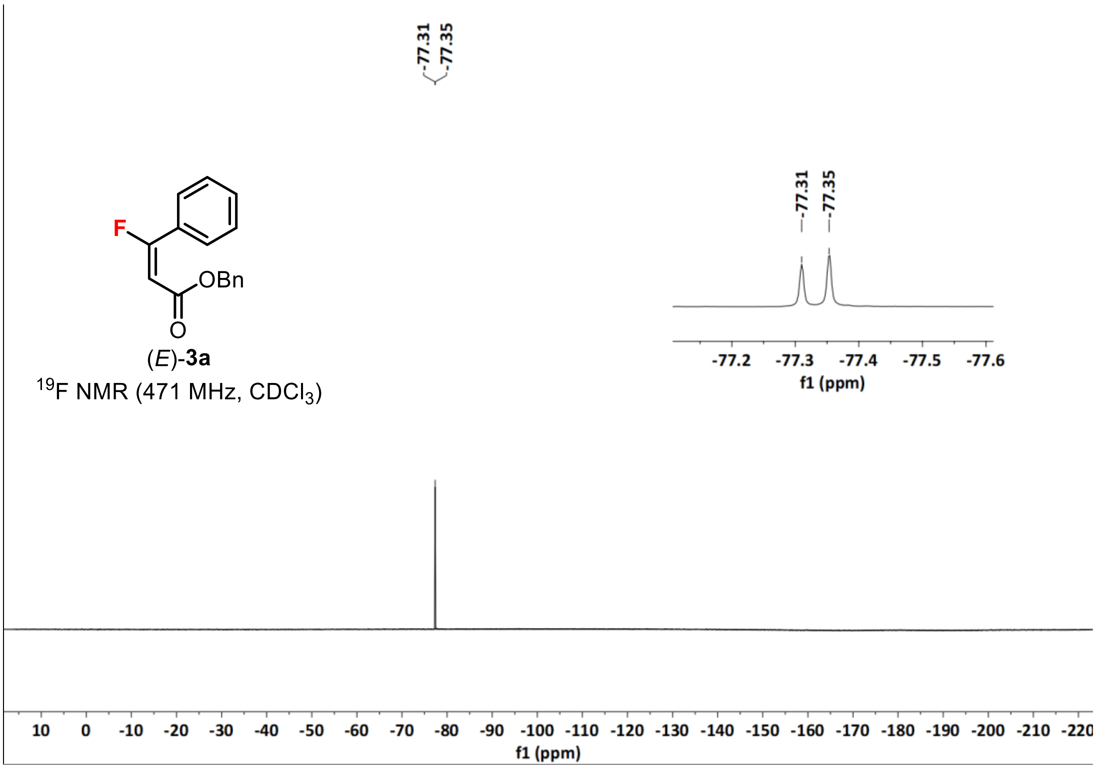

-77.31
-77.35
F
OBn
O
(E)-3a
19F NMR (471 MHz, CDCl3)
-77.2 -77.3 -77.4 -77.5 -77.6
f1 (ppm)
10 0 -10 -20 -30 -40 -50 -60 -70 -80 -90 -100 -110 -120 -130 -140 -150 -160 -170 -180 -190 -200 -210 -220
f1 (ppm)

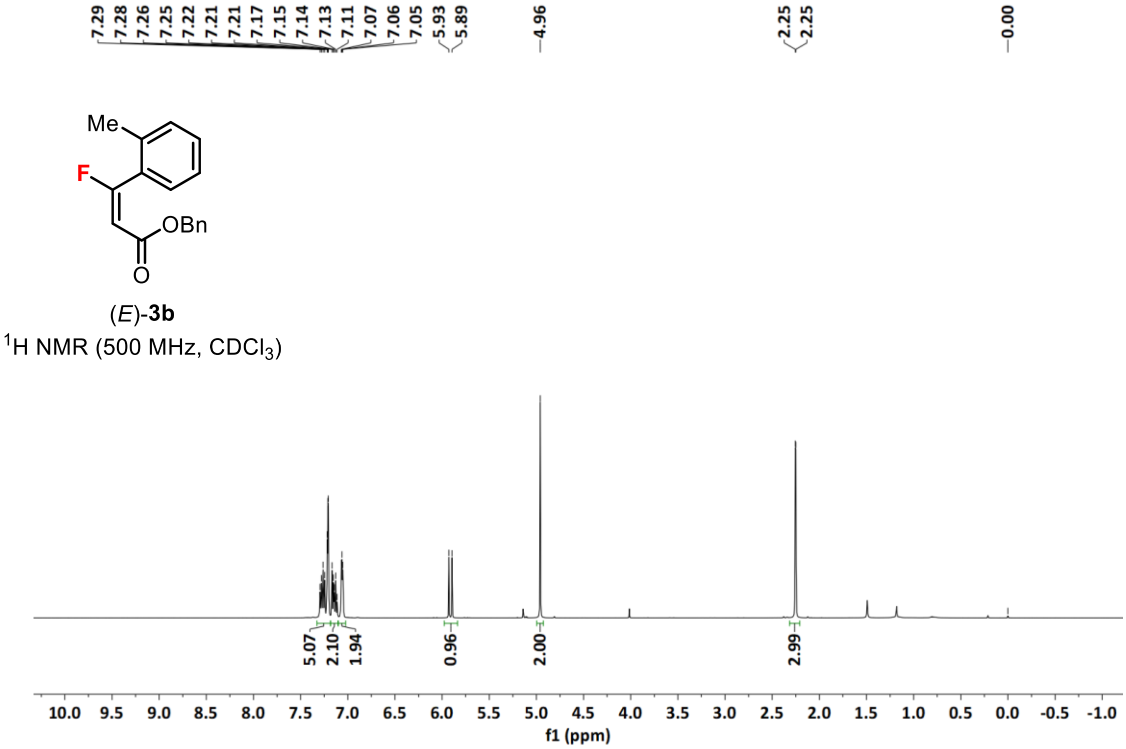

7.29 7.28 7.26 7.25 7.22 7.21 7.21 7.17 7.15 7.14 7.13 7.11 7.07 7.06 7.05 5.93 5.89 4.96 2.25 2.25 0.00
Me
F
OBn
O
(E)-3b
1H NMR (500 MHz, CDCl3)
5.07 2.10 1.94 0.96 2.00 2.99
10.0 9.5 9.0 8.5 8.0 7.5 7.0 6.5 6.0 5.5 5.0 4.5 4.0 3.5 3.0 2.5 2.0 1.5 1.0 0.5 0.0 -0.5 -1.0
f1 (ppm)

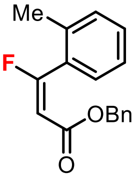

(*E*)-**3b**

^{13}C NMR (126 MHz, CDCl$_3$)

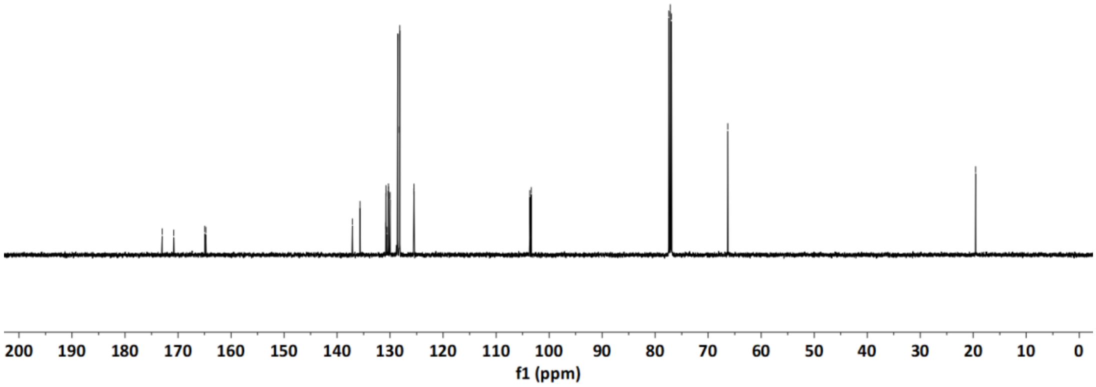

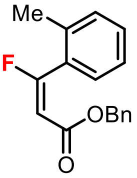

(*E*)-**3b**

^{19}F NMR (471 MHz, CDCl$_3$)

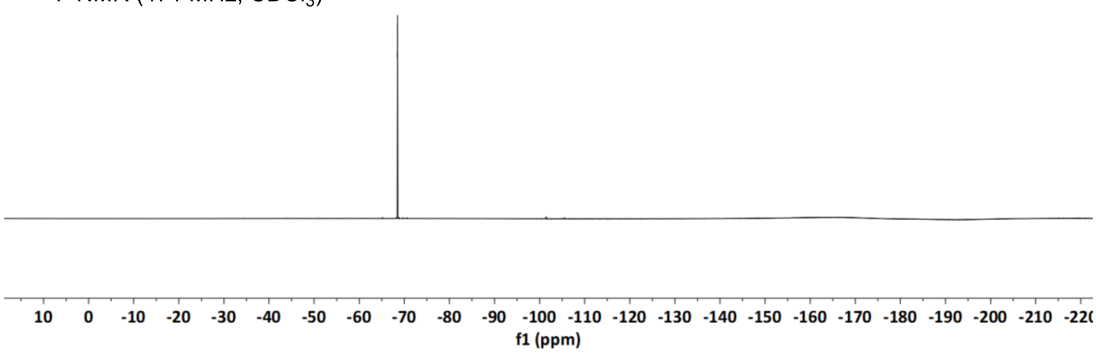

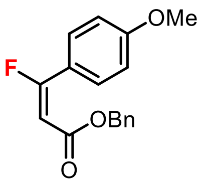

(*E*)-**3c**

^{1}H NMR (500 MHz, $CDCl_3$)

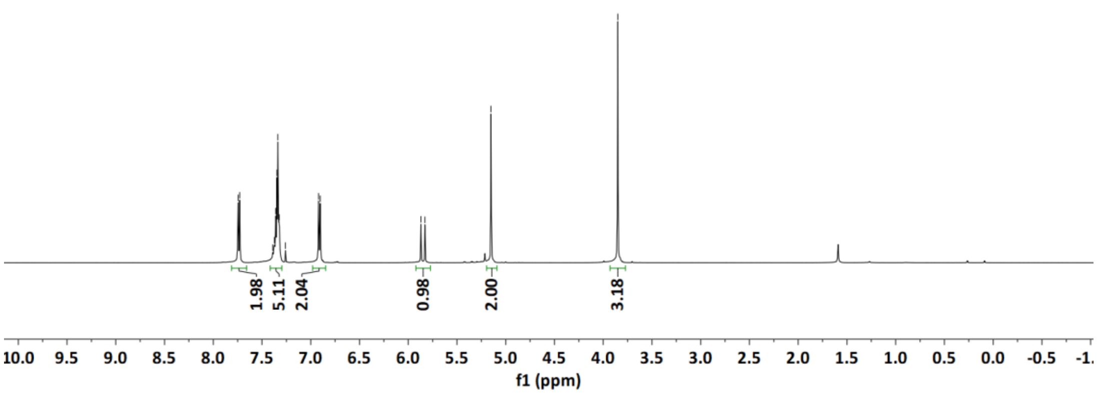

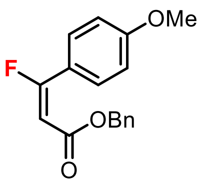

(*E*)-**3c**

^{13}C NMR (126 MHz, $CDCl_3$)

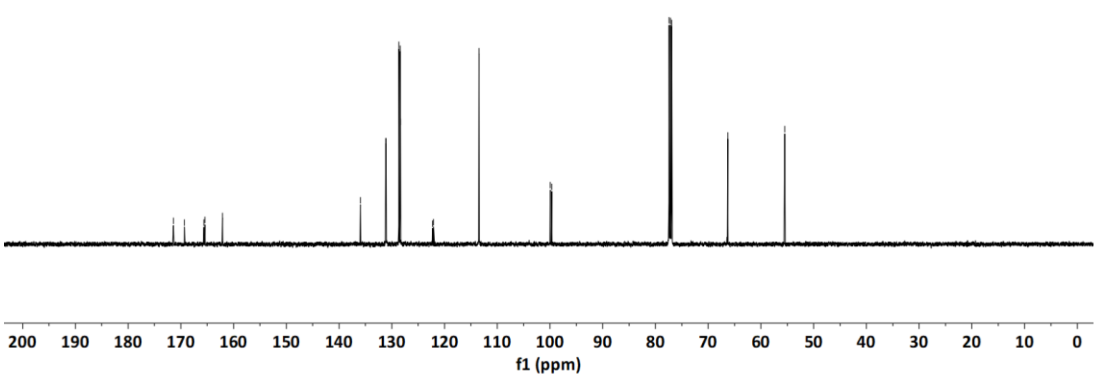

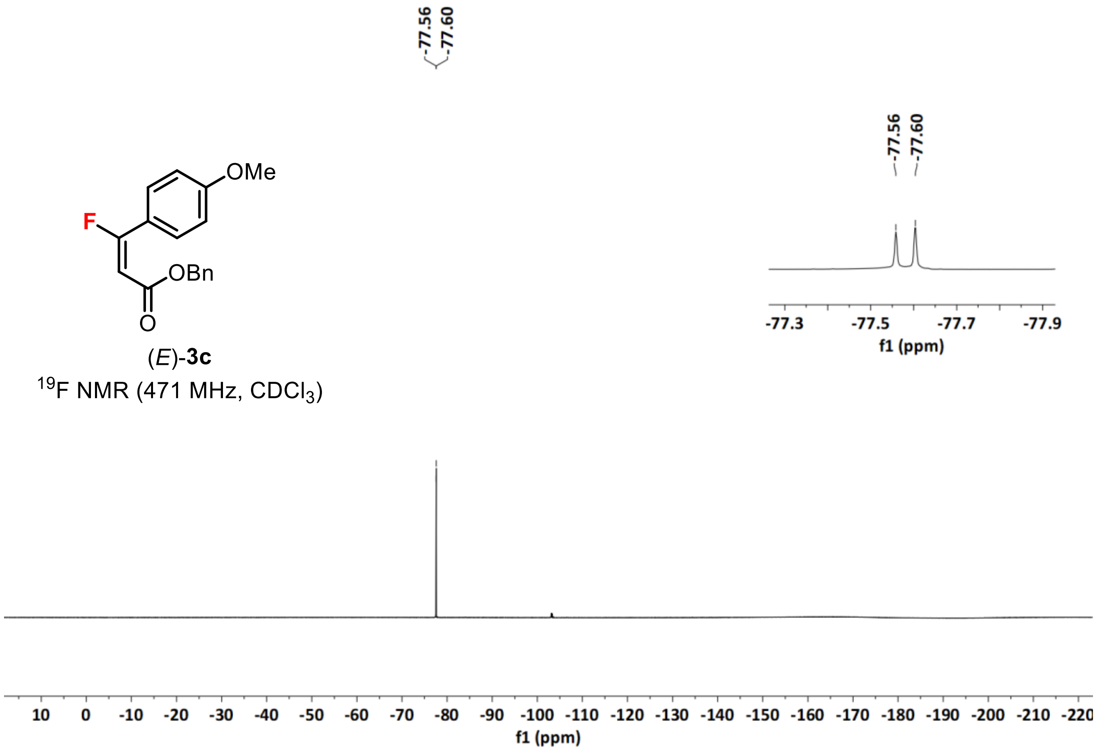

(*E*)-**3c**

^{19}F NMR (471 MHz, $CDCl_3$)

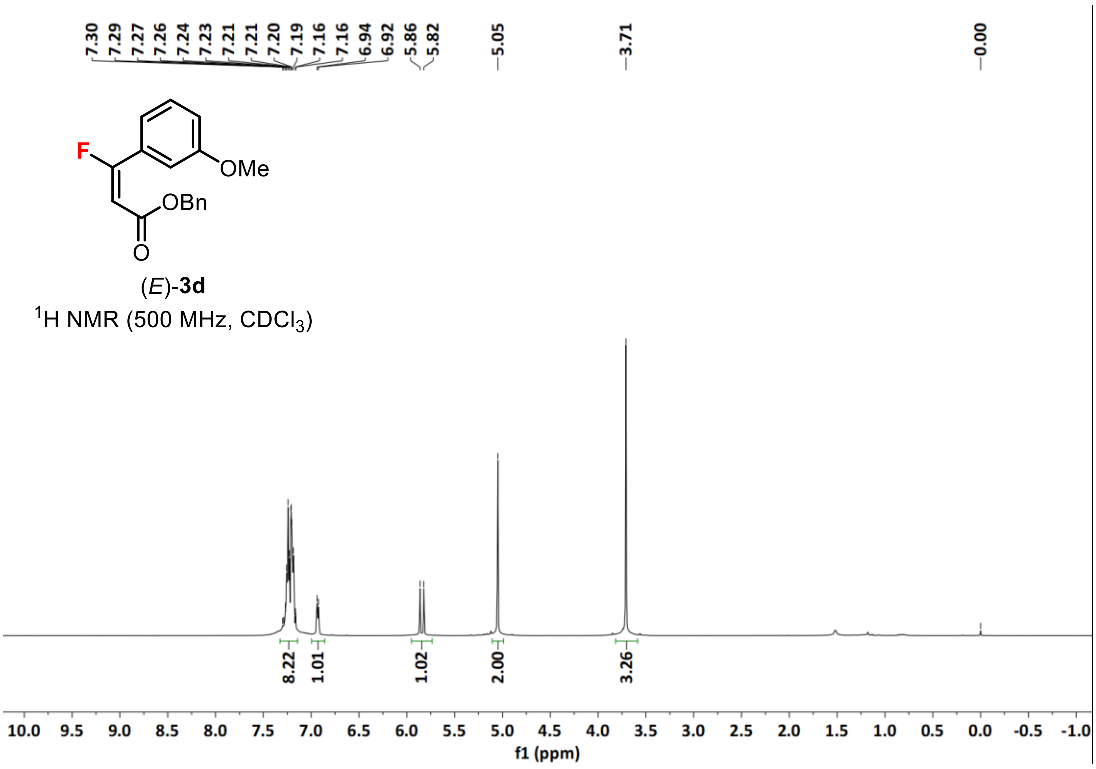

(*E*)-**3d**

^{1}H NMR (500 MHz, $CDCl_3$)

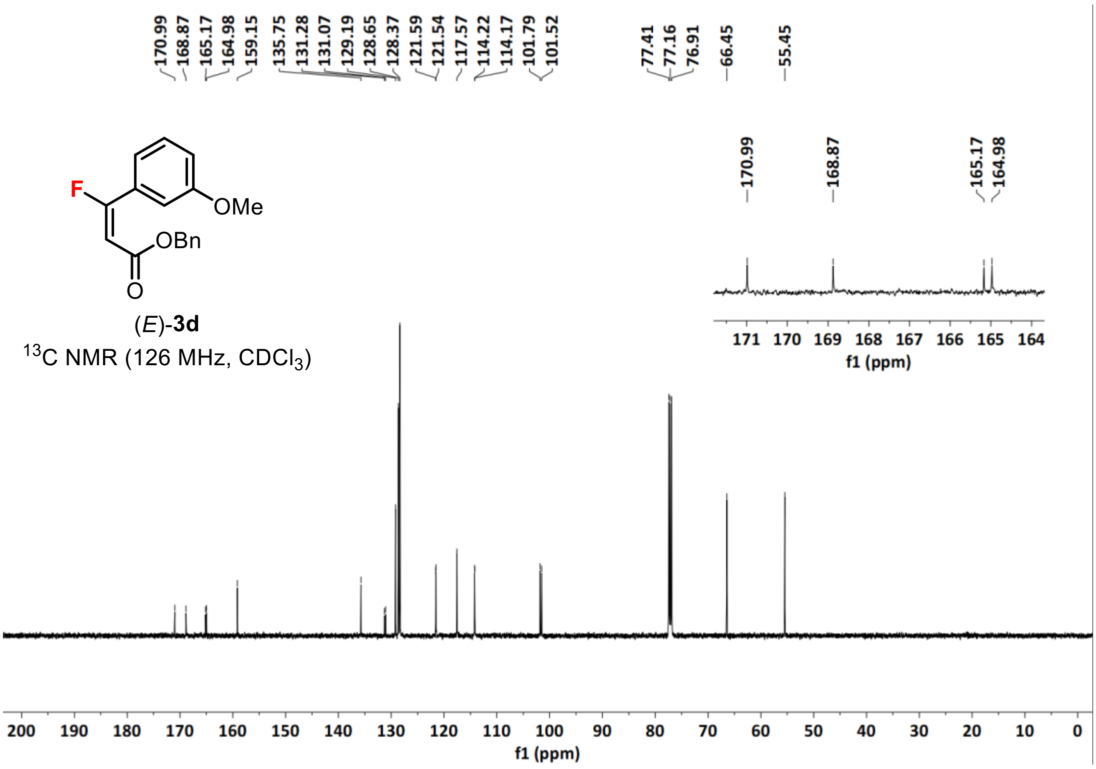
170.99
168.87
165.17
164.98
159.15
135.75
131.28
131.07
129.19
128.65
128.37
121.59
121.54
117.57
114.22
114.17
101.79
101.52
77.41
77.16
76.91
66.45
55.45
F
OMe
OBn
O
(E)-3d
13C NMR (126 MHz, CDCl3)
171 170 169 168 167 166 165 164
f1 (ppm)
200 190 180 170 160 150 140 130 120 110 100 90 80 70 60 50 40 30 20 10 0
f1 (ppm)

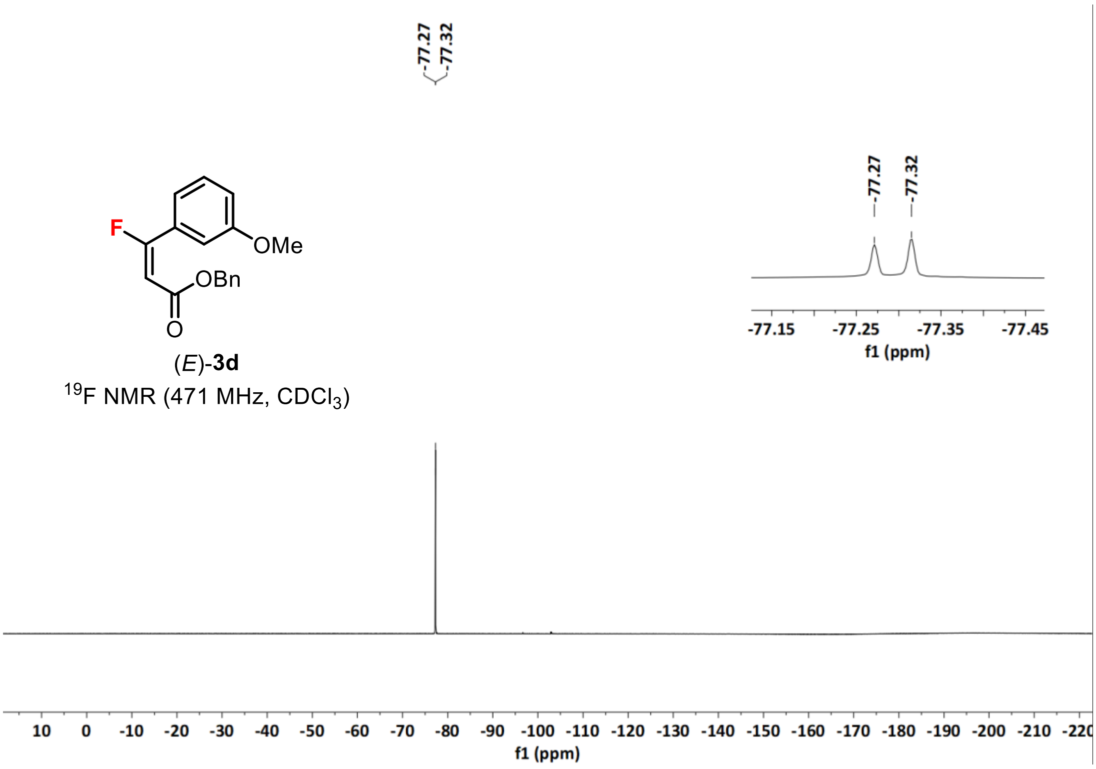
-77.27
-77.32
F
OMe
OBn
O
(E)-3d
19F NMR (471 MHz, CDCl3)
-77.15 -77.25 -77.35 -77.45
f1 (ppm)
10 0 -10 -20 -30 -40 -50 -60 -70 -80 -90 -100 -110 -120 -130 -140 -150 -160 -170 -180 -190 -200 -210 -220
f1 (ppm)

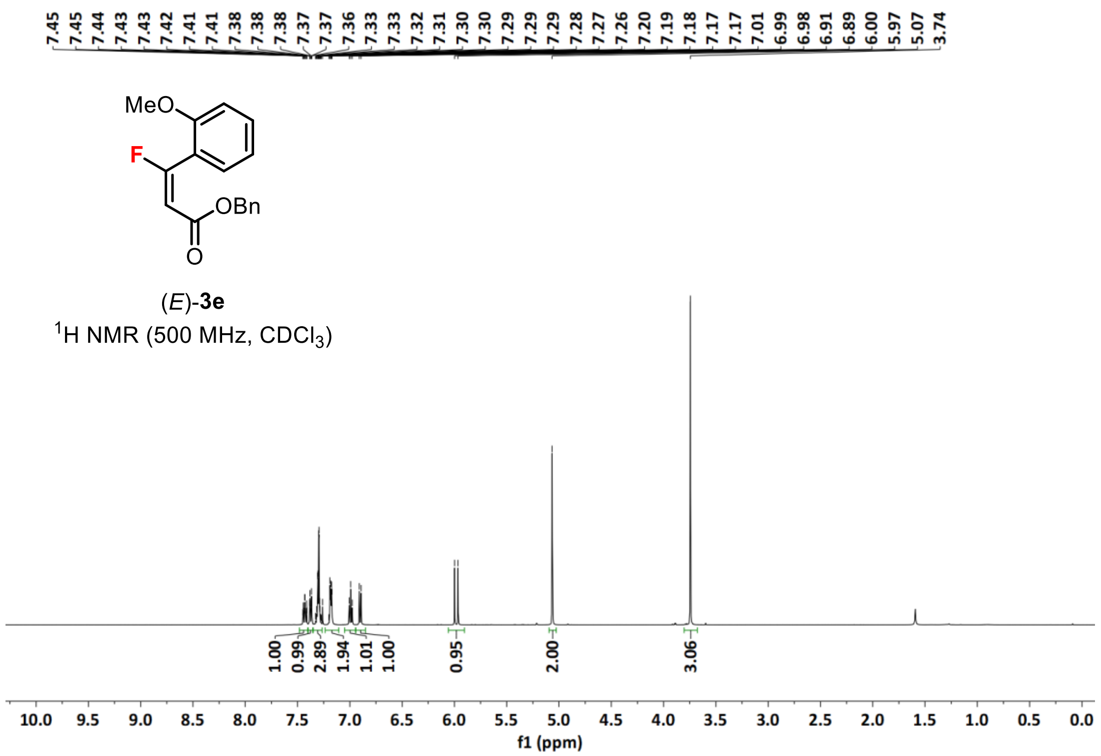
7.45 7.45 7.44 7.43 7.43 7.42 7.41 7.41 7.38 7.38 7.38 7.37 7.37 7.36 7.33 7.33 7.32 7.31 7.30 7.30 7.29 7.29 7.29 7.28 7.27 7.26 7.20 7.19 7.18 7.17 7.17 7.01 6.99 6.98 6.91 6.89 6.00 5.97 5.07 3.74
MeO
F
OBn
O
(*E*)-**3e**
^{1}H NMR (500 MHz, $CDCl_3$)
1.00 0.99 2.89 1.94 1.01 1.00 0.95 2.00 3.06
f1 (ppm)

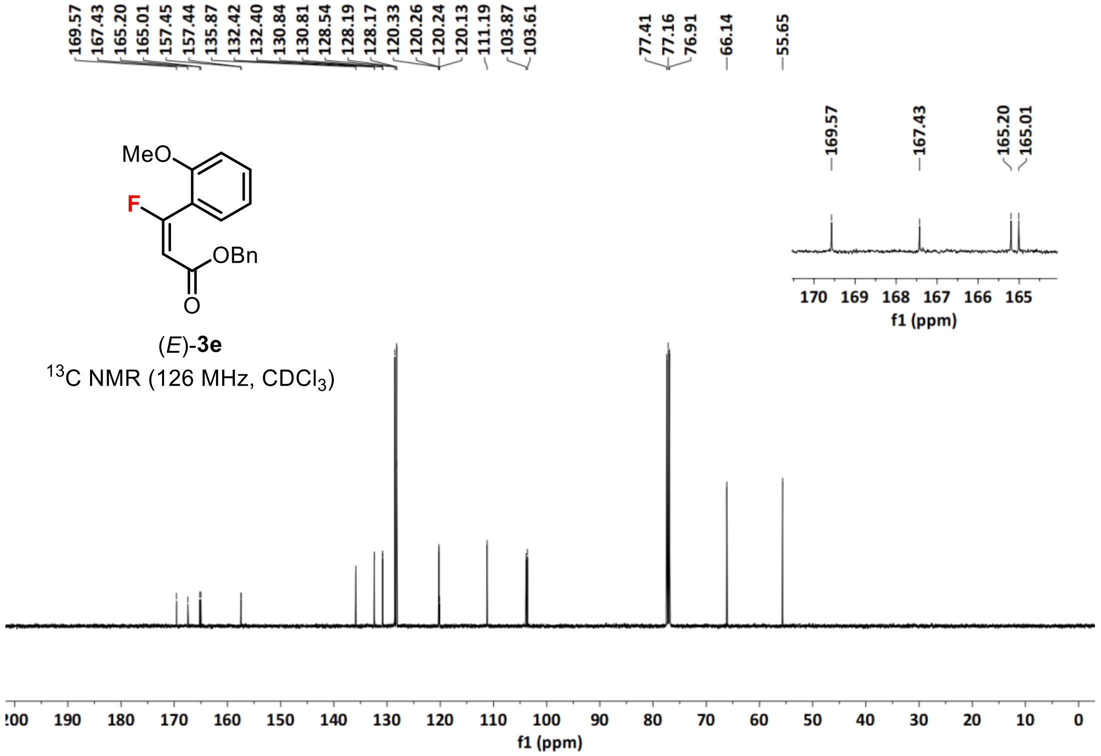
169.57 167.43 165.20 165.01 157.45 157.44 135.87 132.42 132.40 130.84 130.81 128.54 128.19 128.17 120.33 120.26 120.24 120.13 111.19 103.87 103.61
77.41 77.16 76.91 66.14 55.65
MeO
F
OBn
O
(*E*)-**3e**
^{13}C NMR (126 MHz, $CDCl_3$)
169.57 167.43 165.20 165.01
f1 (ppm)
f1 (ppm)

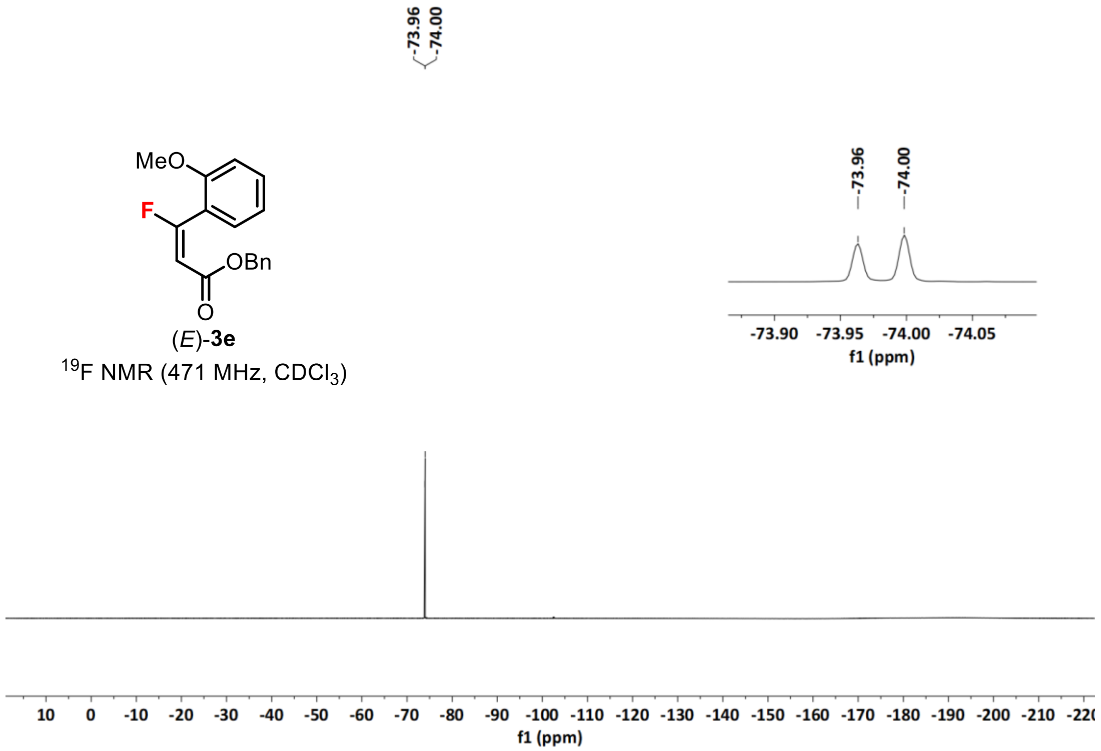

(*E*)-**3e**

^{19}F NMR (471 MHz, $CDCl_3$)

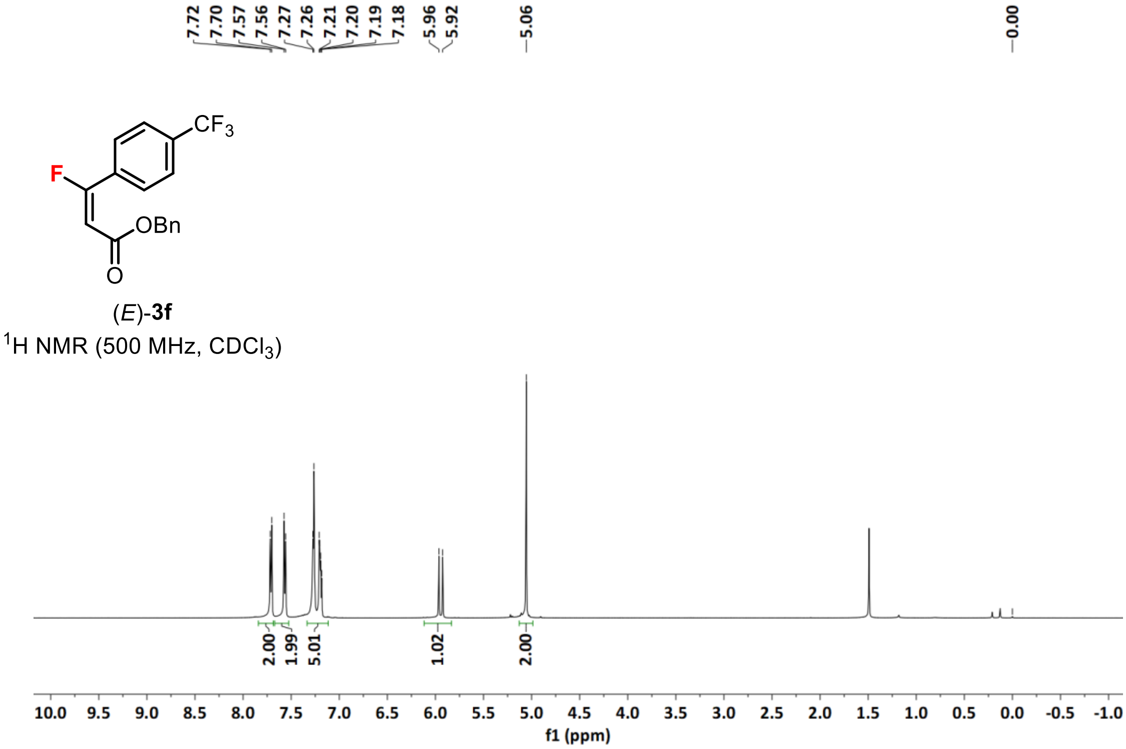

(*E*)-**3f**

^{1}H NMR (500 MHz, $CDCl_3$)

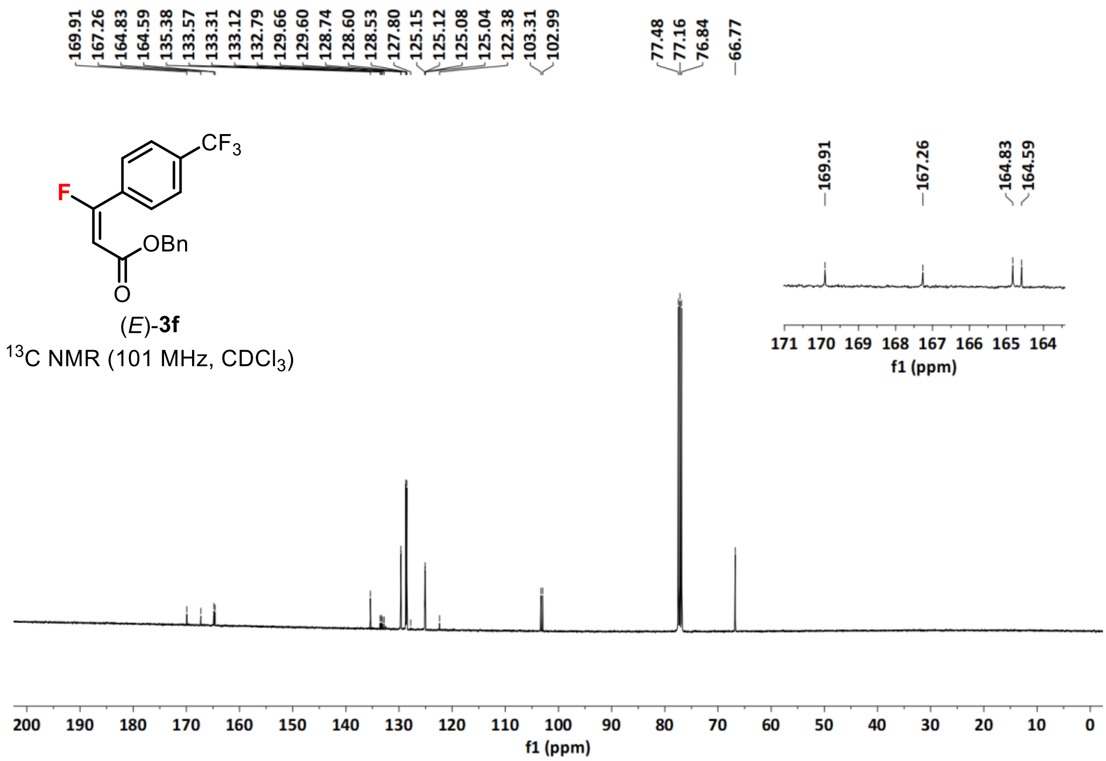
169.91
167.26
164.83
164.59
135.38
133.57
133.31
133.12
132.79
129.66
129.60
128.74
128.60
128.53
127.80
125.15
125.12
125.08
125.04
122.38
103.31
102.99
77.48
77.16
76.84
66.77
CF3
F
OBn
O
(E)-3f
13C NMR (101 MHz, CDCl3)
f1 (ppm)

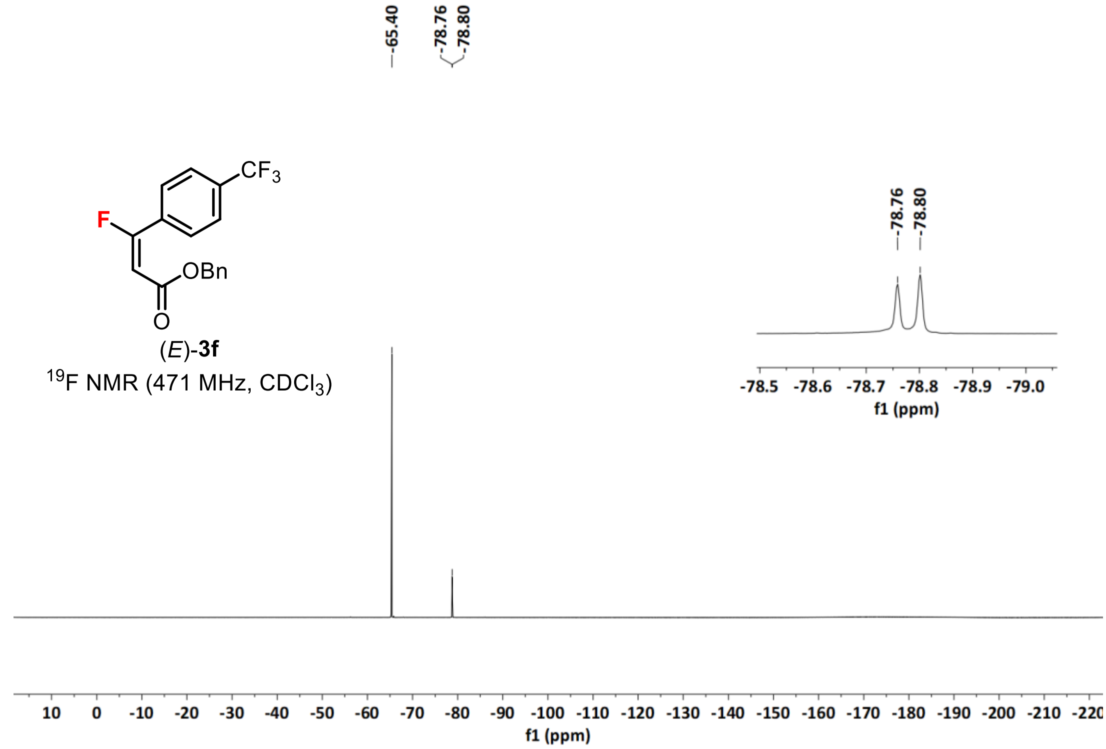
-65.40
-78.76
-78.80
CF3
F
OBn
O
(E)-3f
19F NMR (471 MHz, CDCl3)
f1 (ppm)

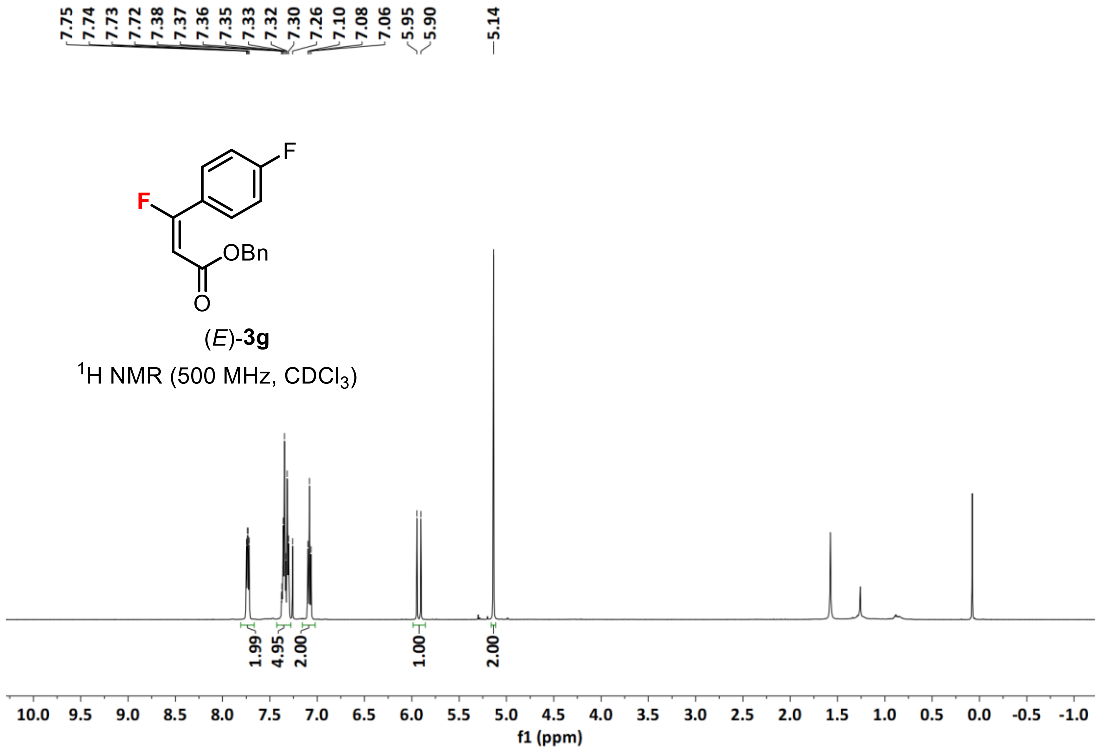

(*E*)-**3g**

^{1}H NMR (500 MHz, $CDCl_3$)

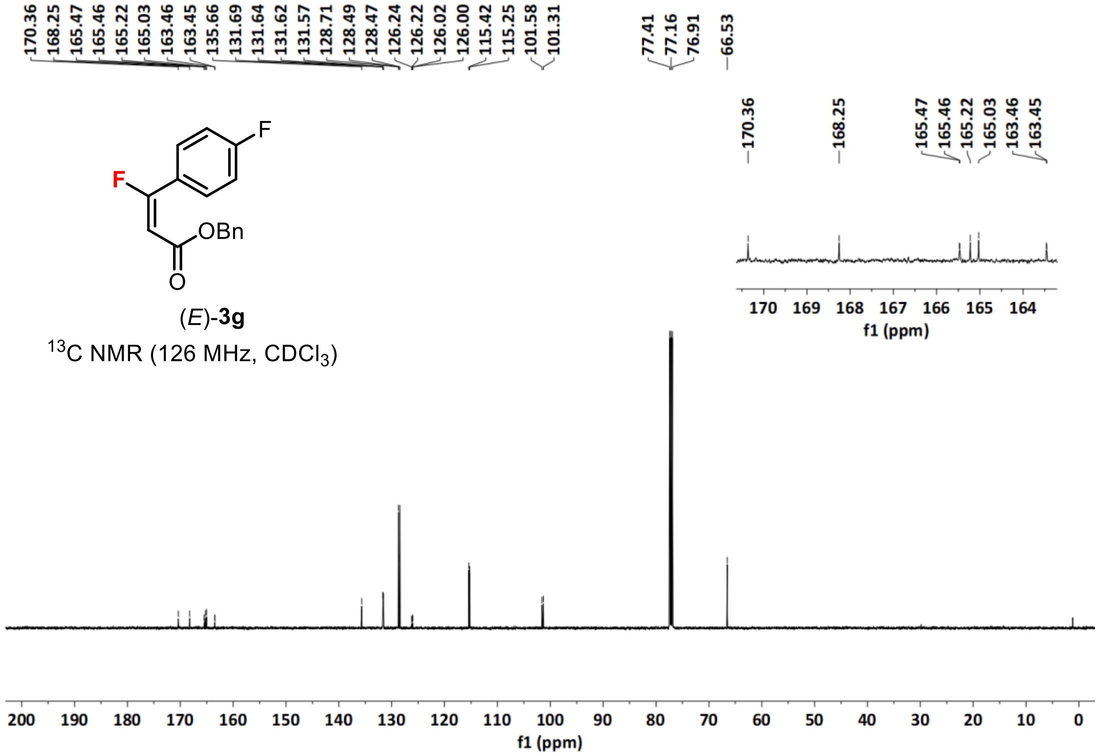

(*E*)-**3g**

^{13}C NMR (126 MHz, $CDCl_3$)

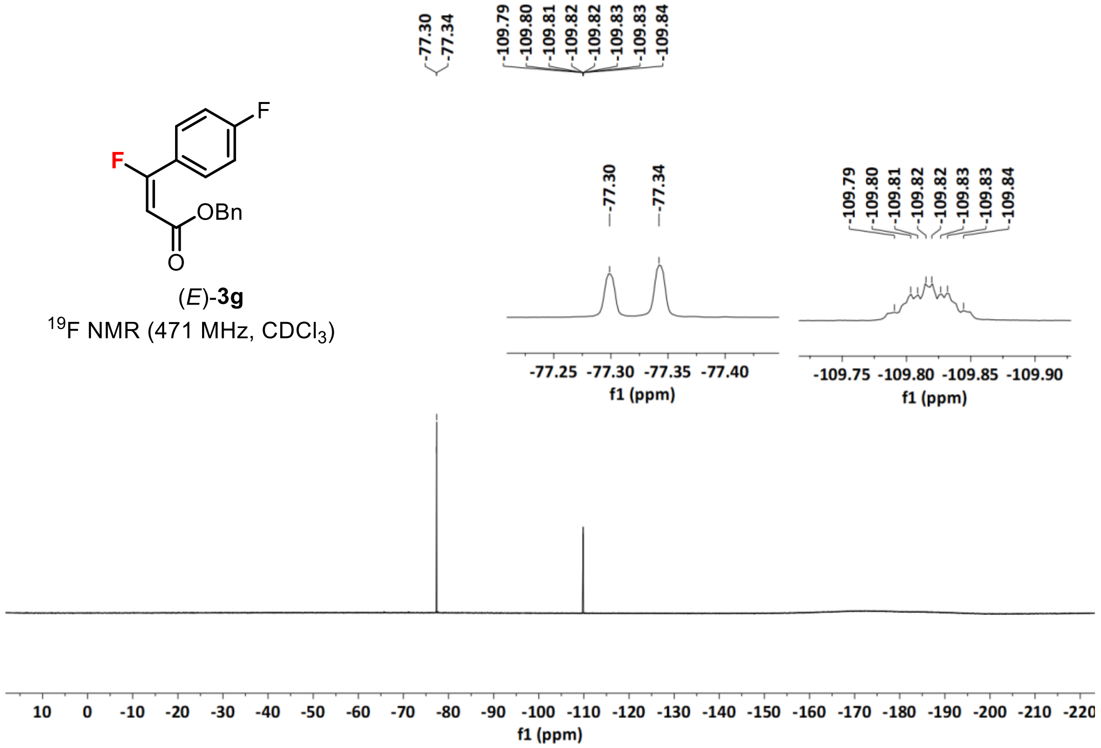

-77.30
-77.34
-109.79
-109.80
-109.81
-109.82
-109.82
-109.83
-109.83
-109.84
F
F
OBn
O
(E)-3g
19F NMR (471 MHz, CDCl3)
-77.25 -77.30 -77.35 -77.40
f1 (ppm)
-109.75 -109.80 -109.85 -109.90
f1 (ppm)
10 0 -10 -20 -30 -40 -50 -60 -70 -80 -90 -100 -110 -120 -130 -140 -150 -160 -170 -180 -190 -200 -210 -220
f1 (ppm)

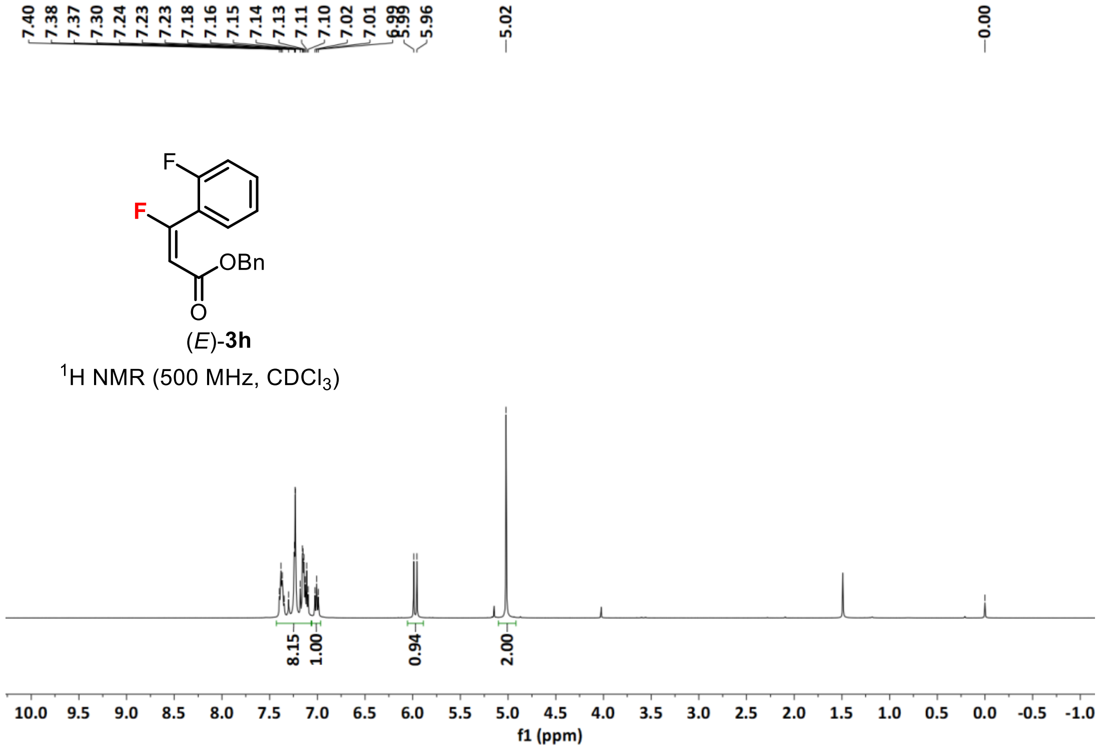

7.40
7.38
7.37
7.30
7.24
7.23
7.23
7.18
7.16
7.15
7.14
7.13
7.11
7.10
7.02
7.01
6.99
5.99
5.96
5.02
0.00
F
F
OBn
O
(E)-3h
1H NMR (500 MHz, CDCl3)
8.15
1.00
0.94
2.00
10.0 9.5 9.0 8.5 8.0 7.5 7.0 6.5 6.0 5.5 5.0 4.5 4.0 3.5 3.0 2.5 2.0 1.5 1.0 0.5 0.0 -0.5 -1.0
f1 (ppm)

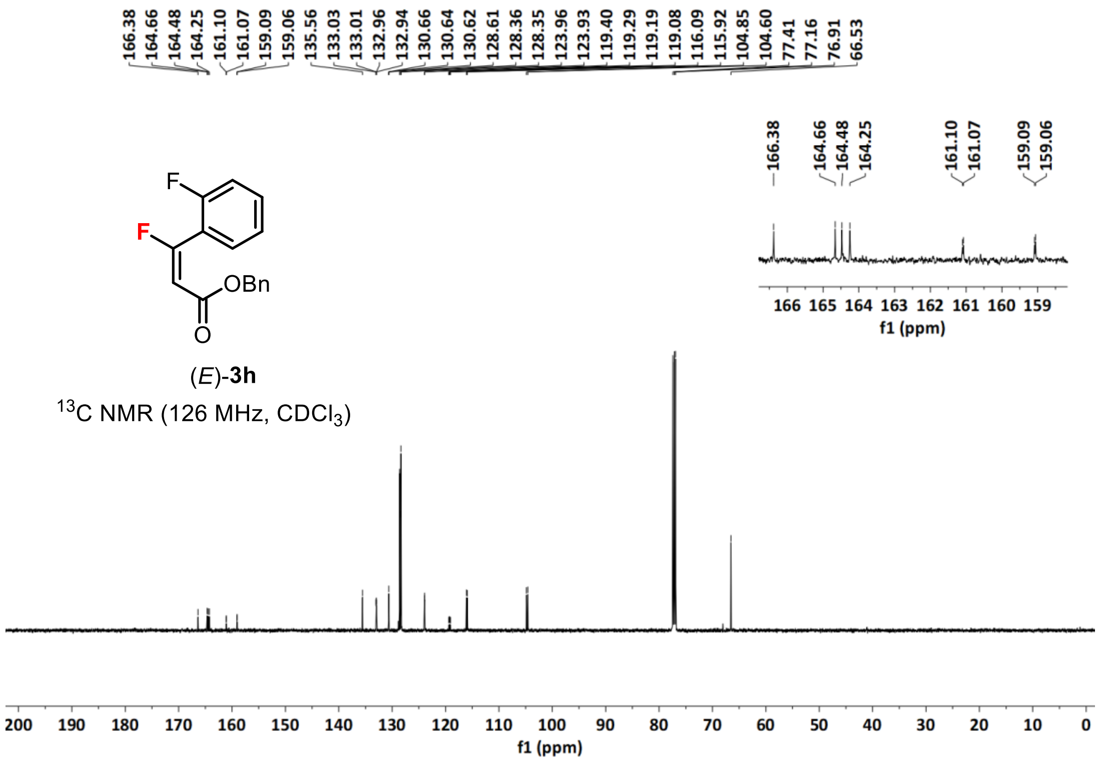

(*E*)-**3h**

^{13}C NMR (126 MHz, $CDCl_3$)

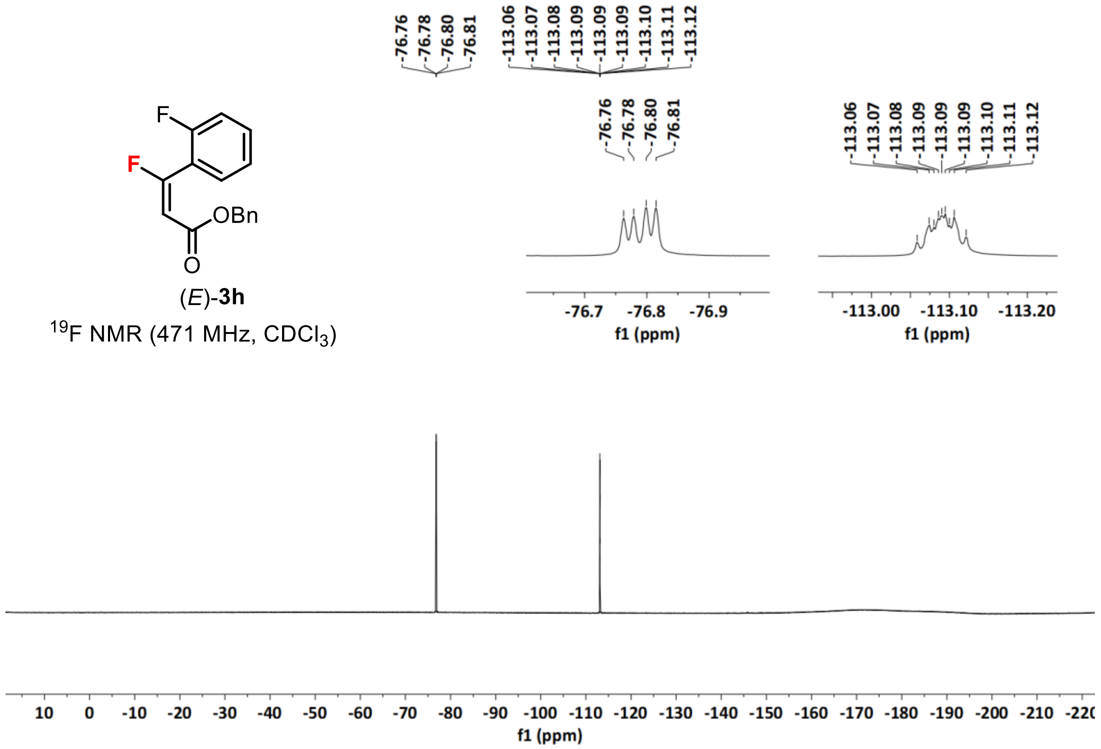

(*E*)-**3h**

^{19}F NMR (471 MHz, $CDCl_3$)

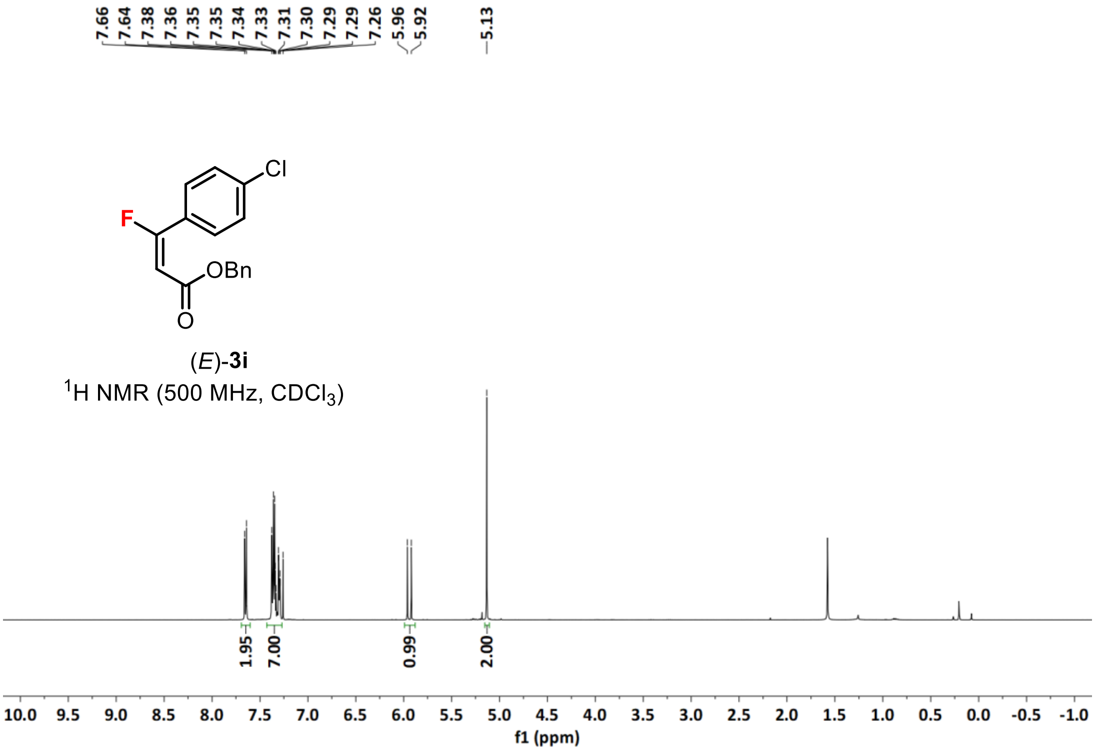

(*E*)-**3i**

^{1}H NMR (500 MHz, $CDCl_3$)

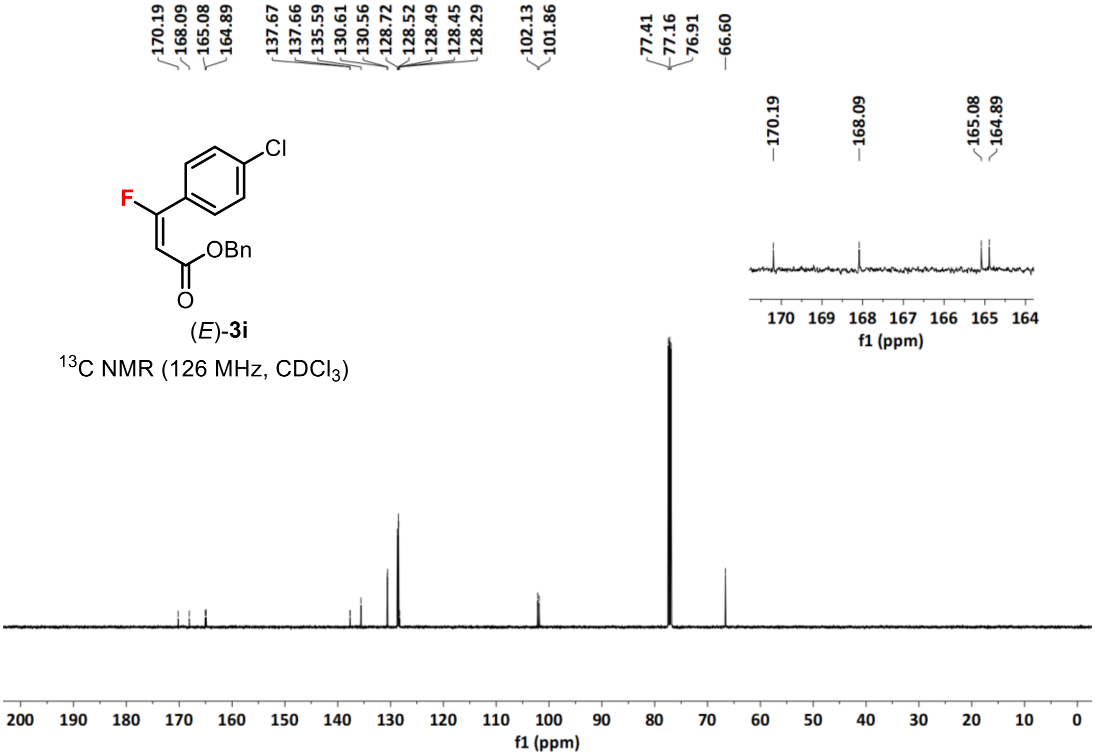

(*E*)-**3i**

^{13}C NMR (126 MHz, $CDCl_3$)

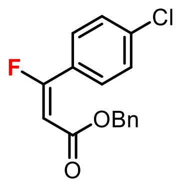

(*E*)-**3i**

^{19}F NMR (471 MHz, $CDCl_3$)

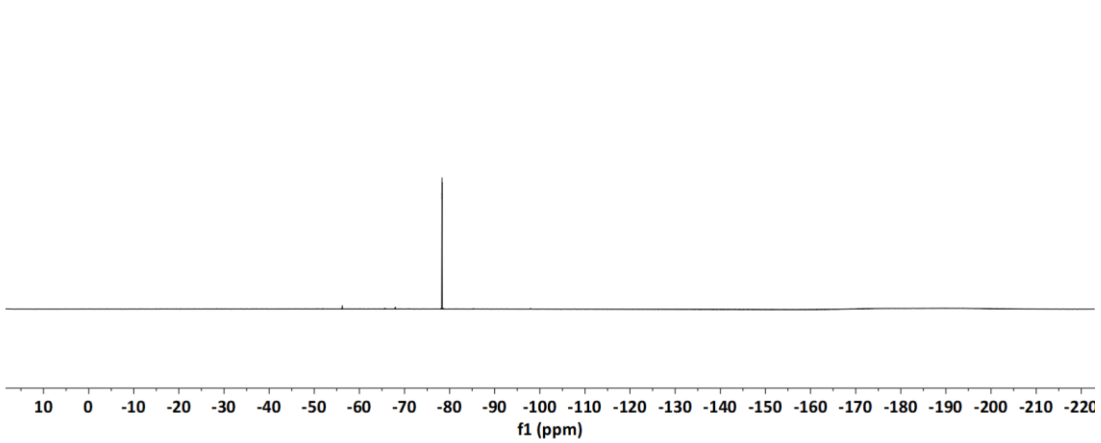

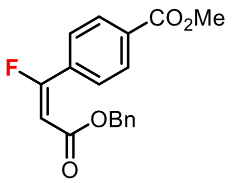

(*E*)-**3j**

^{1}H NMR (500 MHz, $CDCl_3$)

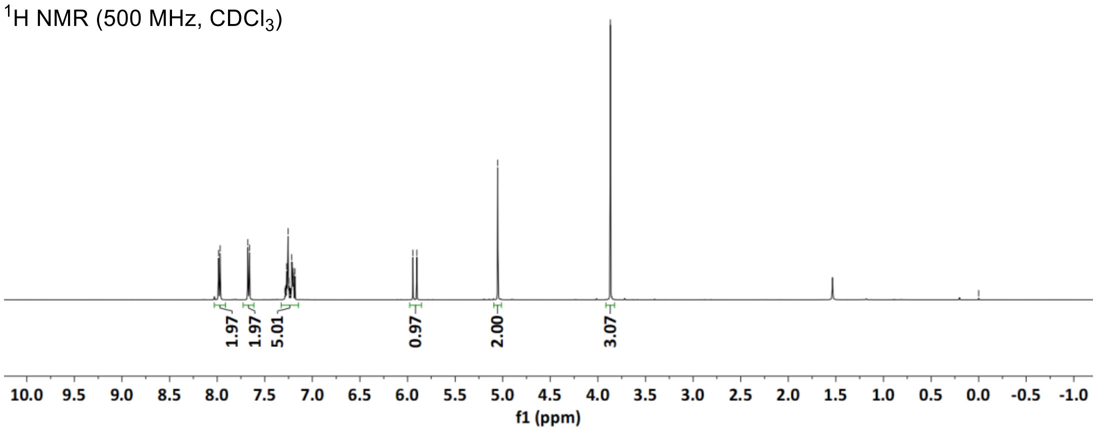

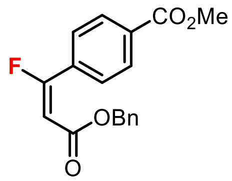

(*E*)-**3j**

^{13}C NMR (126 MHz, $CDCl_3$)

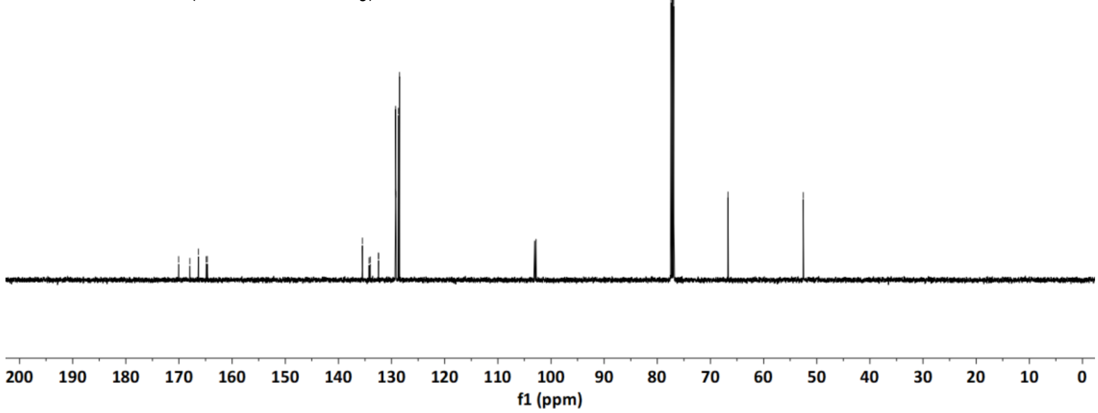

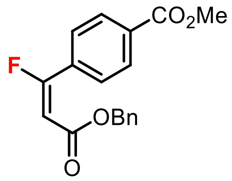

(*E*)-**3j**

^{19}F NMR (471 MHz, $CDCl_3$)

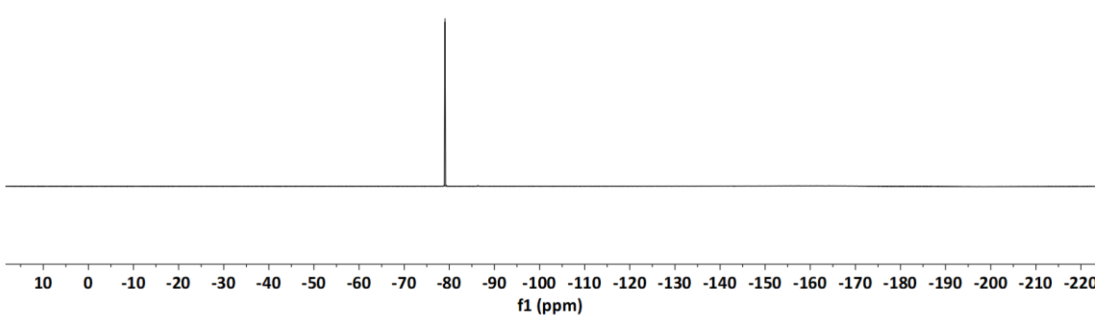

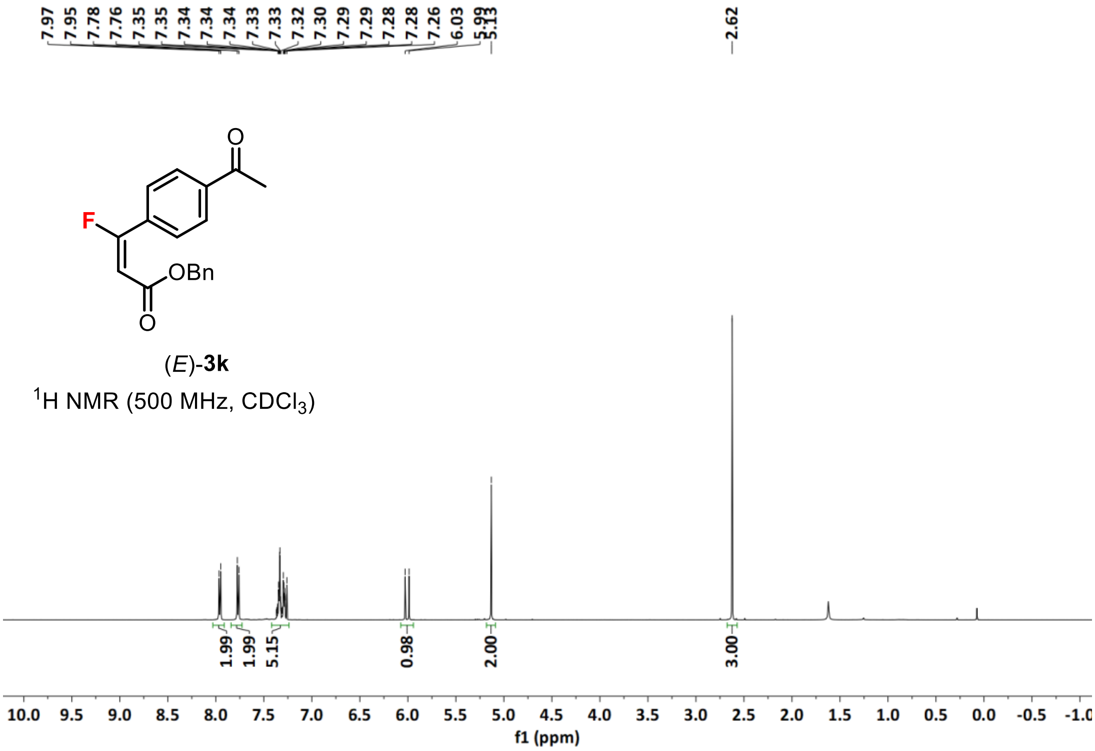
7.97
7.95
7.78
7.76
7.35
7.35
7.34
7.34
7.34
7.33
7.33
7.32
7.30
7.29
7.29
7.28
7.28
7.26
6.03
5.99
5.13
2.62
F
OBn
O
(E)-3k
1H NMR (500 MHz, CDCl3)
1.99
1.99
5.15
0.98
2.00
3.00
f1 (ppm)

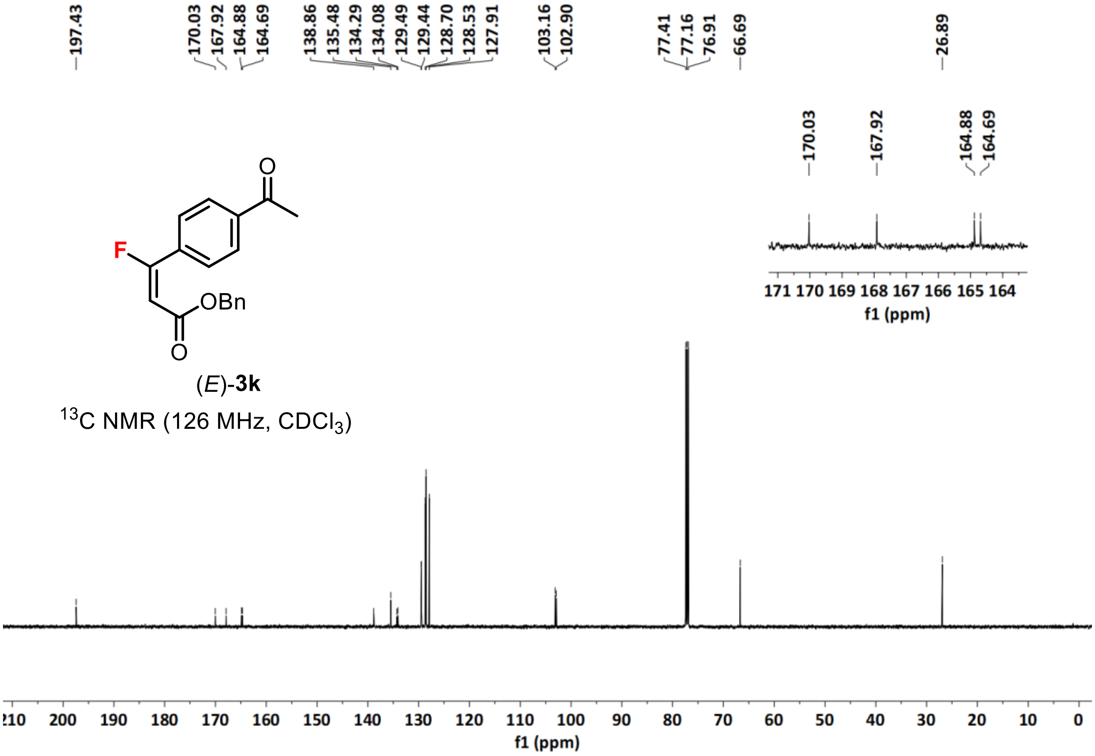
197.43
170.03
167.92
164.88
164.69
138.86
135.48
134.29
134.08
129.49
129.44
128.70
128.53
127.91
103.16
102.90
77.41
77.16
76.91
66.69
26.89
F
OBn
O
(E)-3k
13C NMR (126 MHz, CDCl3)
f1 (ppm)

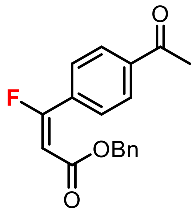

(*E*)-**3k**

^{19}F NMR (471 MHz, CDCl$_3$)

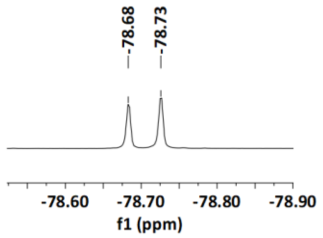

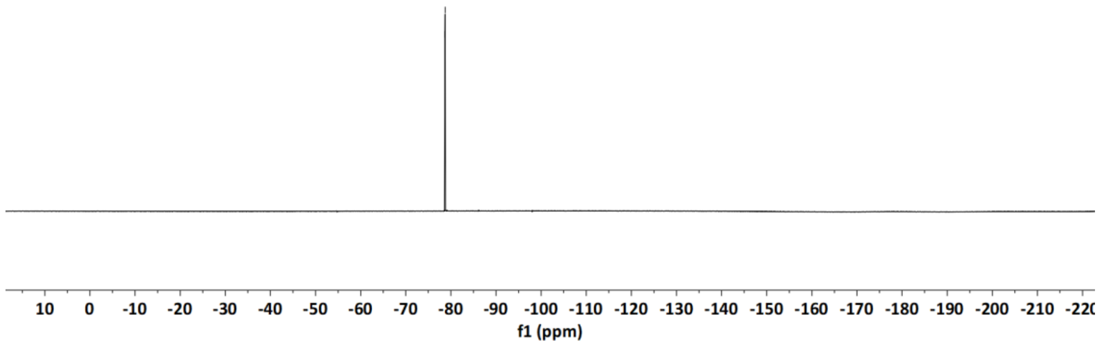

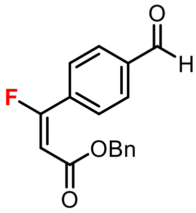

(*E*)-**3l**

^{1}H NMR (500 MHz, CDCl$_3$)

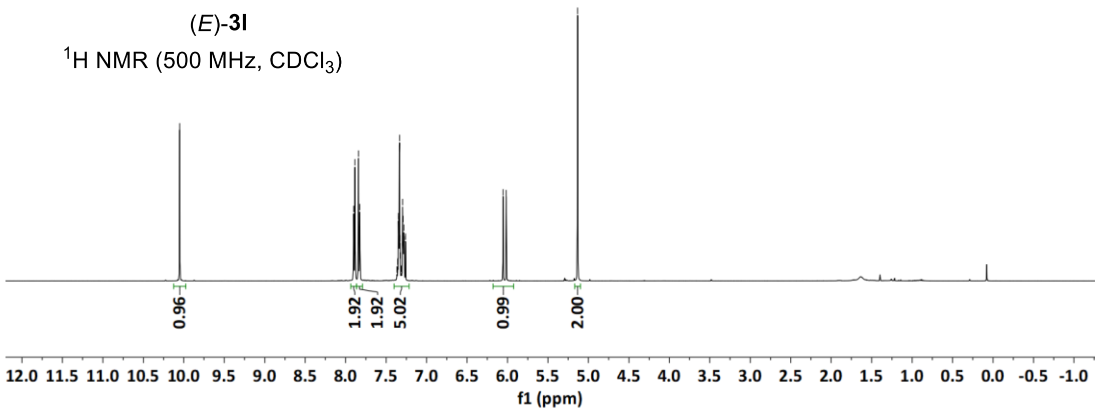

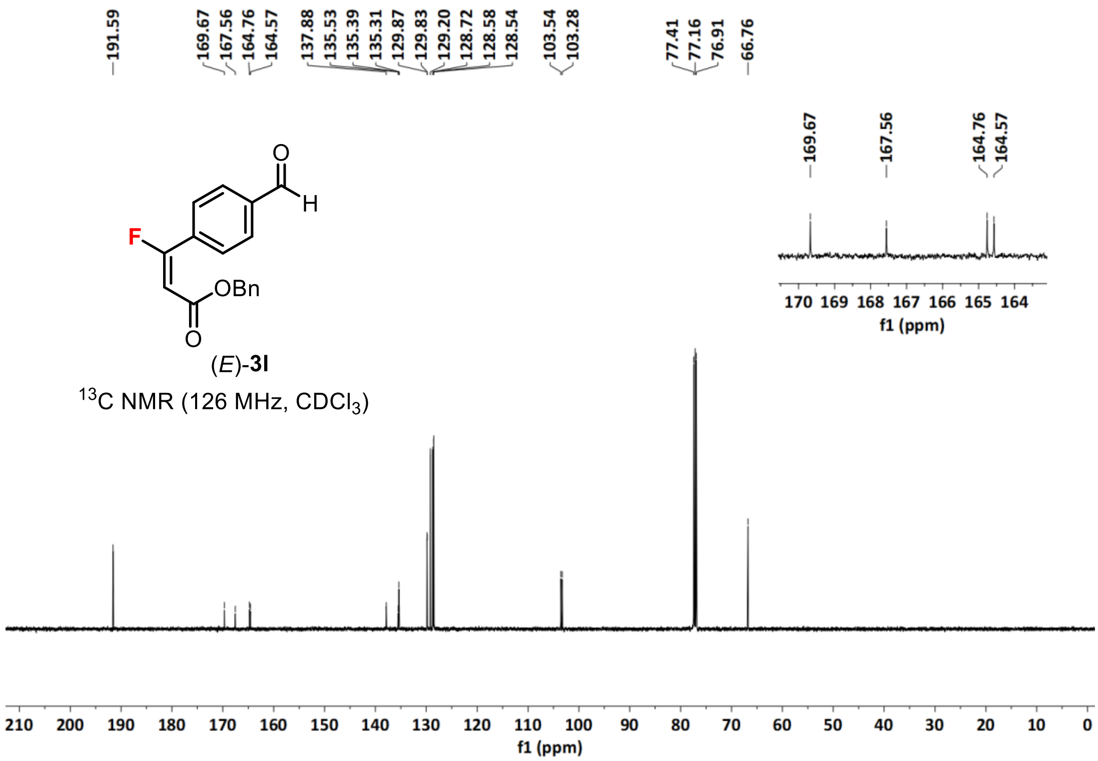

(*E*)-**3l**

^{13}C NMR (126 MHz, $CDCl_3$)

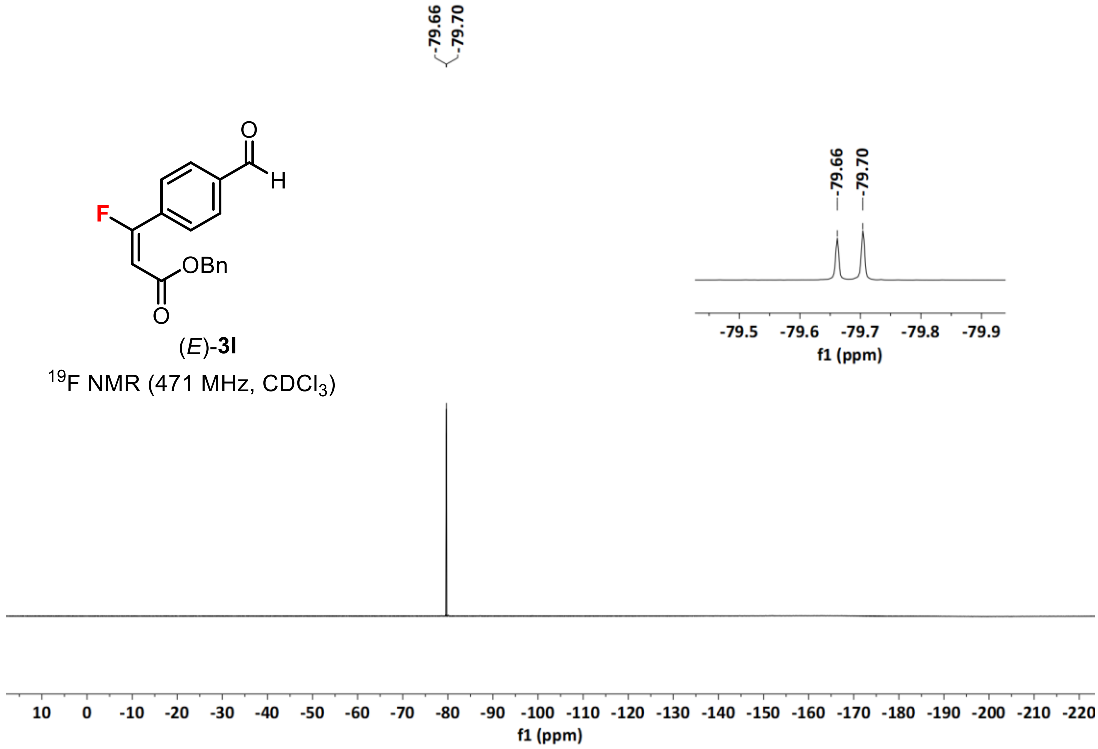

(*E*)-**3l**

^{19}F NMR (471 MHz, $CDCl_3$)

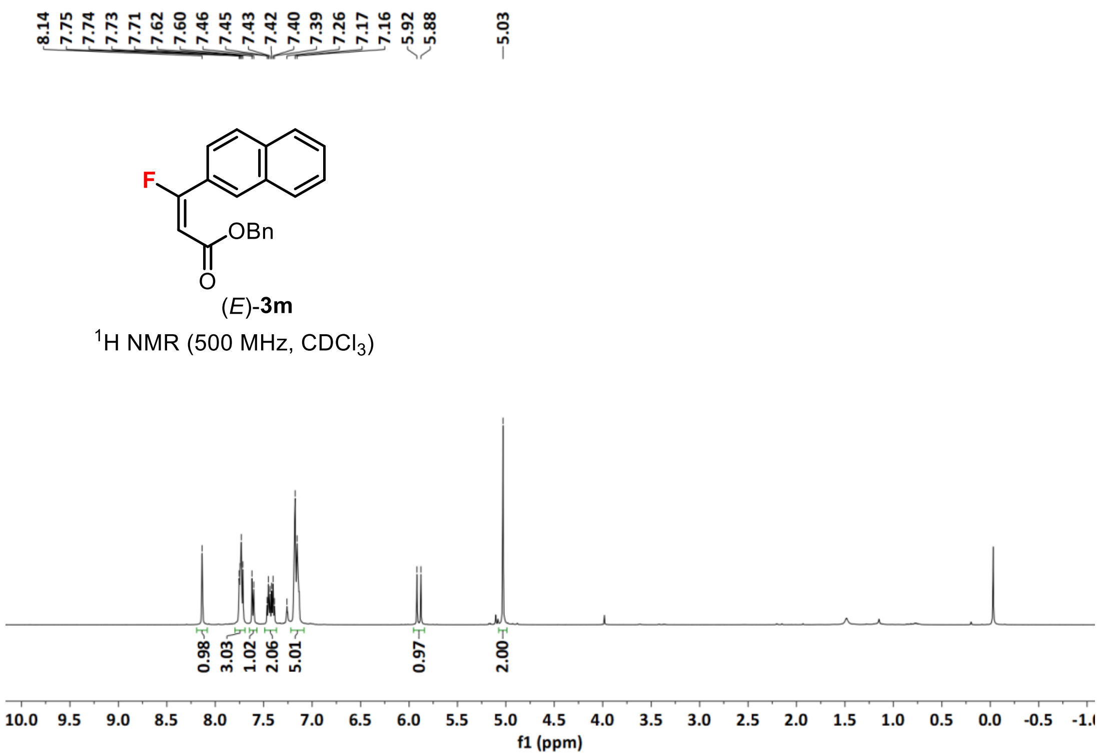

(*E*)-**3m**

^{1}H NMR (500 MHz, $CDCl_3$)

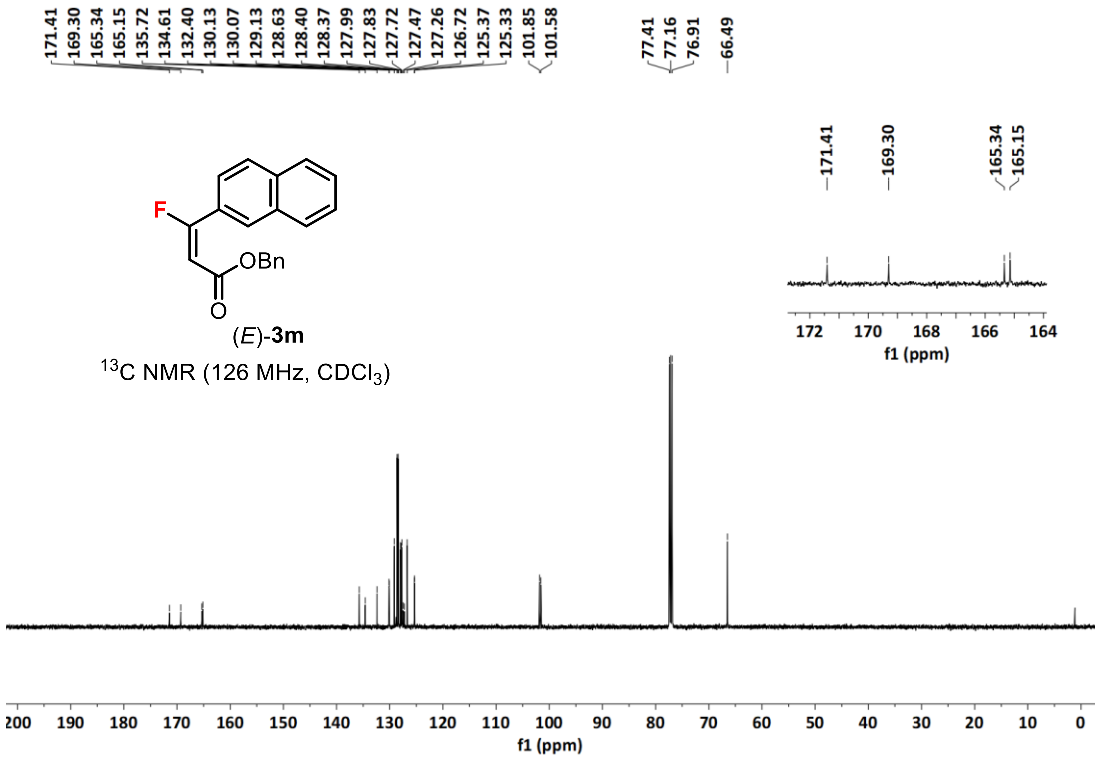

(*E*)-**3m**

^{13}C NMR (126 MHz, $CDCl_3$)

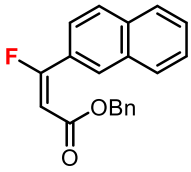

(*E*)-**3m**

^{19}F NMR (471 MHz, $CDCl_3$)

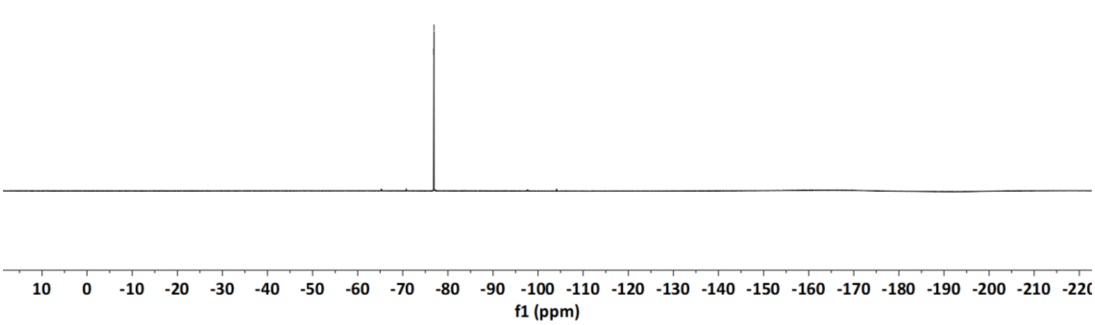

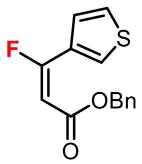

(*E*)-**3n**

^{1}H NMR (500 MHz, $CDCl_3$)

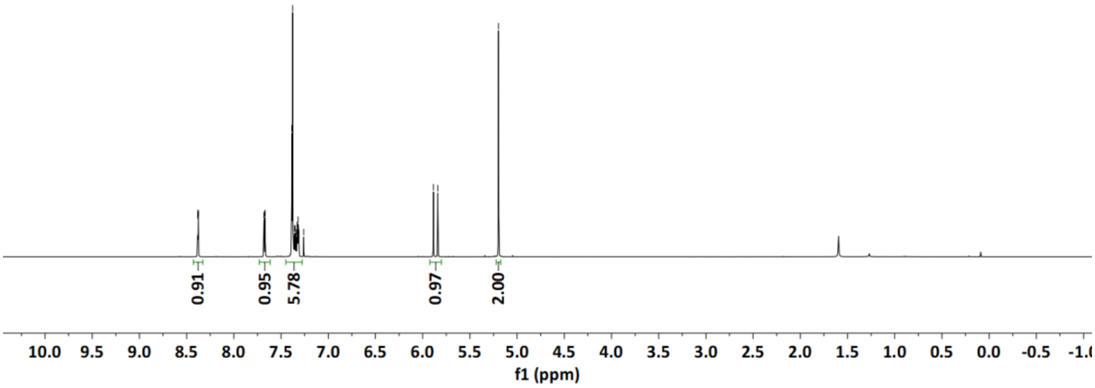

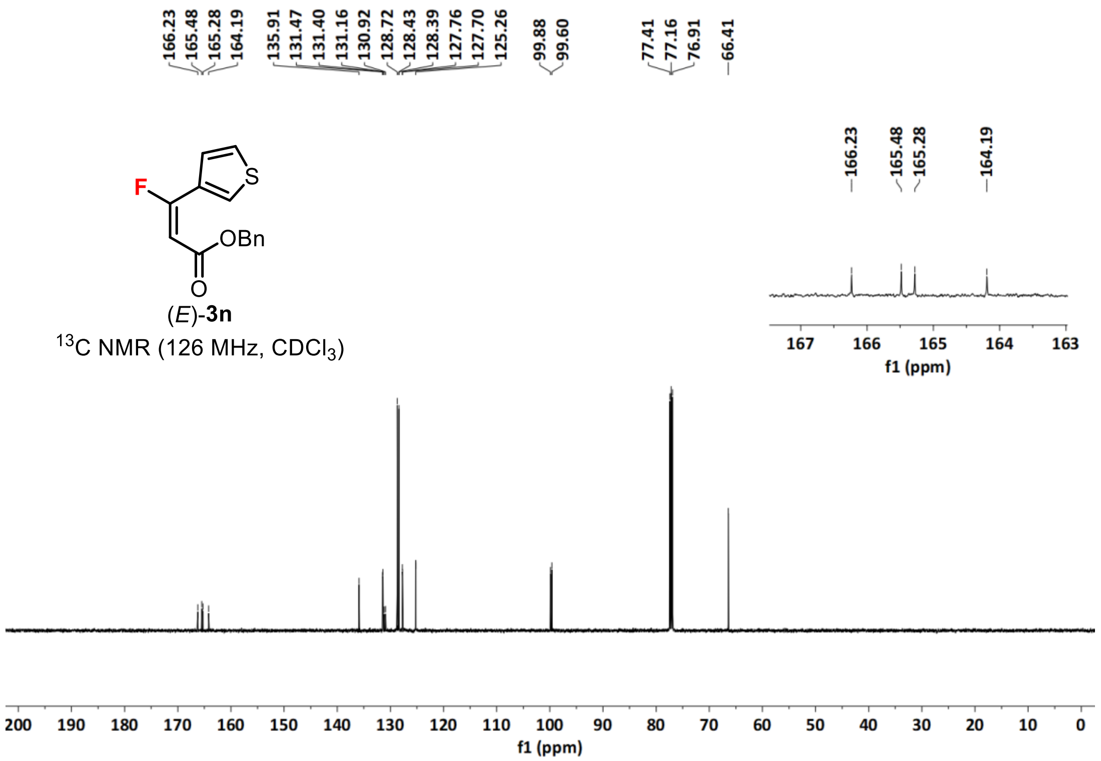
166.23
165.48
165.28
164.19
135.91
131.47
131.40
131.16
130.92
128.72
128.43
128.39
127.76
127.70
125.26
99.88
99.60
77.41
77.16
76.91
66.41
F
S
OBn
O
(E)-3n
13C NMR (126 MHz, CDCl3)
f1 (ppm)

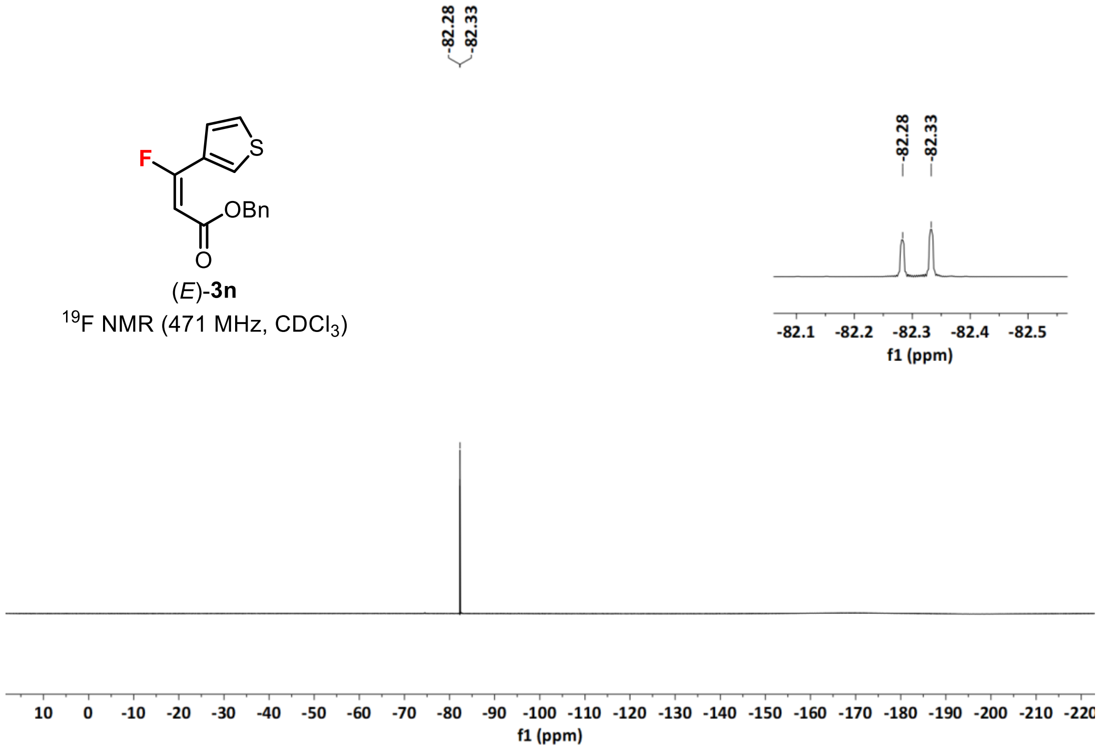
-82.28
-82.33
F
S
OBn
O
(E)-3n
19F NMR (471 MHz, CDCl3)
f1 (ppm)

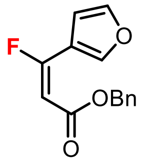

(*E*)-**3o**
^{1}H NMR (500 MHz, $CDCl_3$)

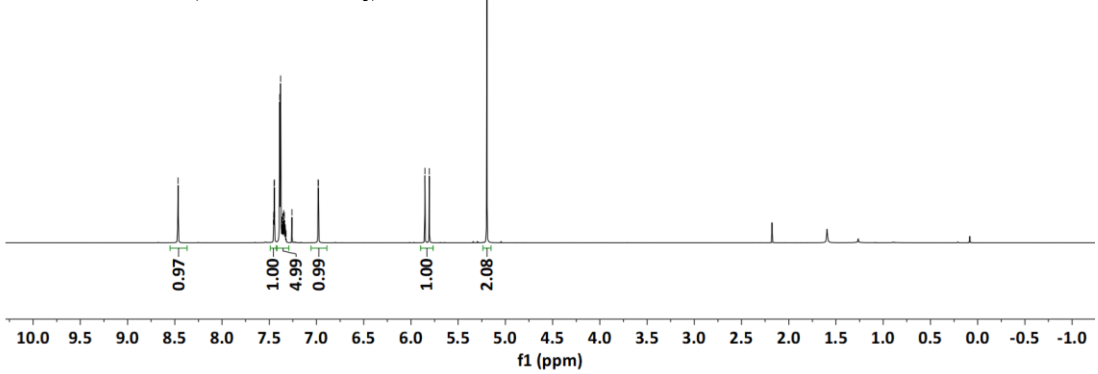

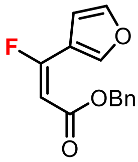

(*E*)-**3o**
^{13}C NMR (126 MHz, $CDCl_3$)

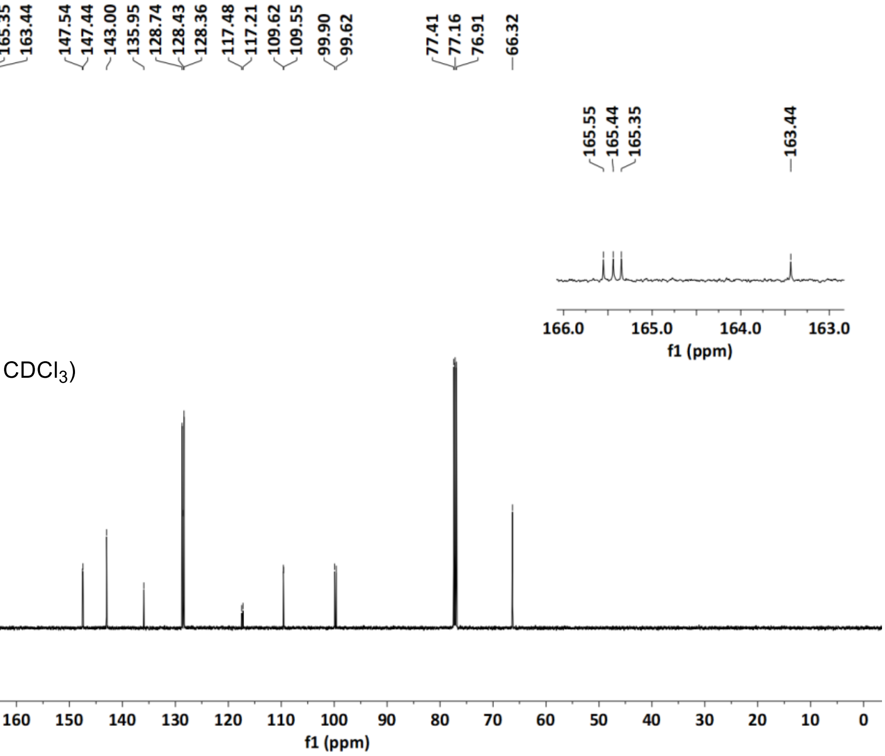

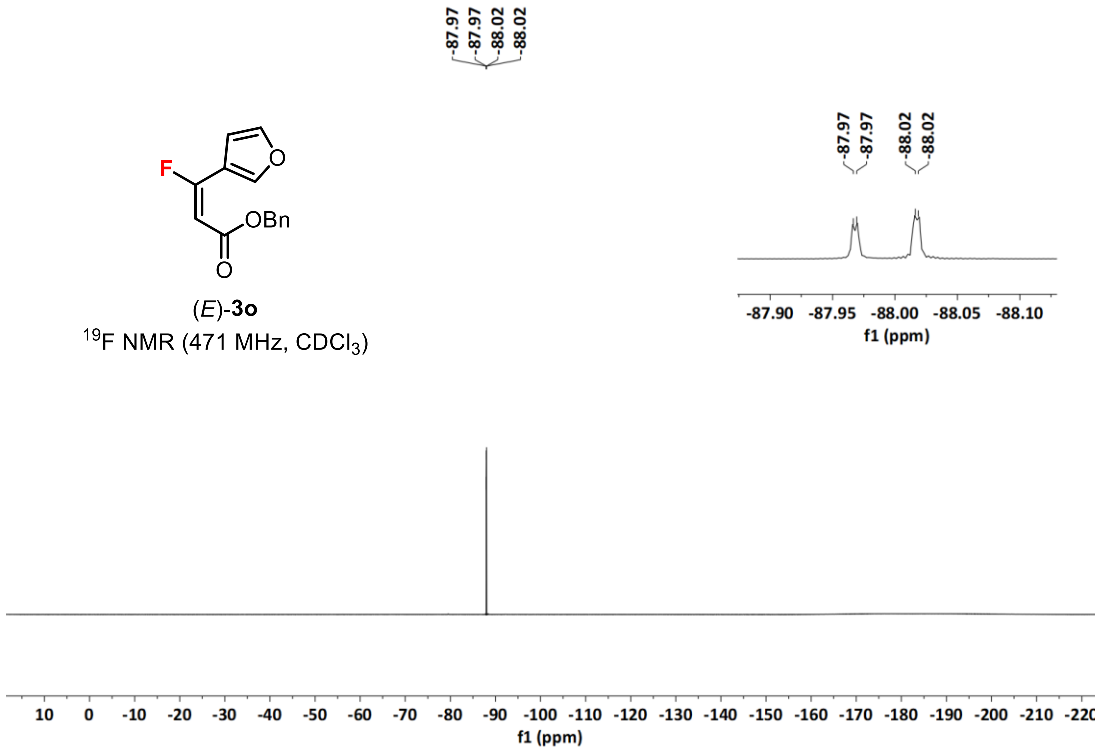

(*E*)-**3o**

^{19}F NMR (471 MHz, $CDCl_3$)

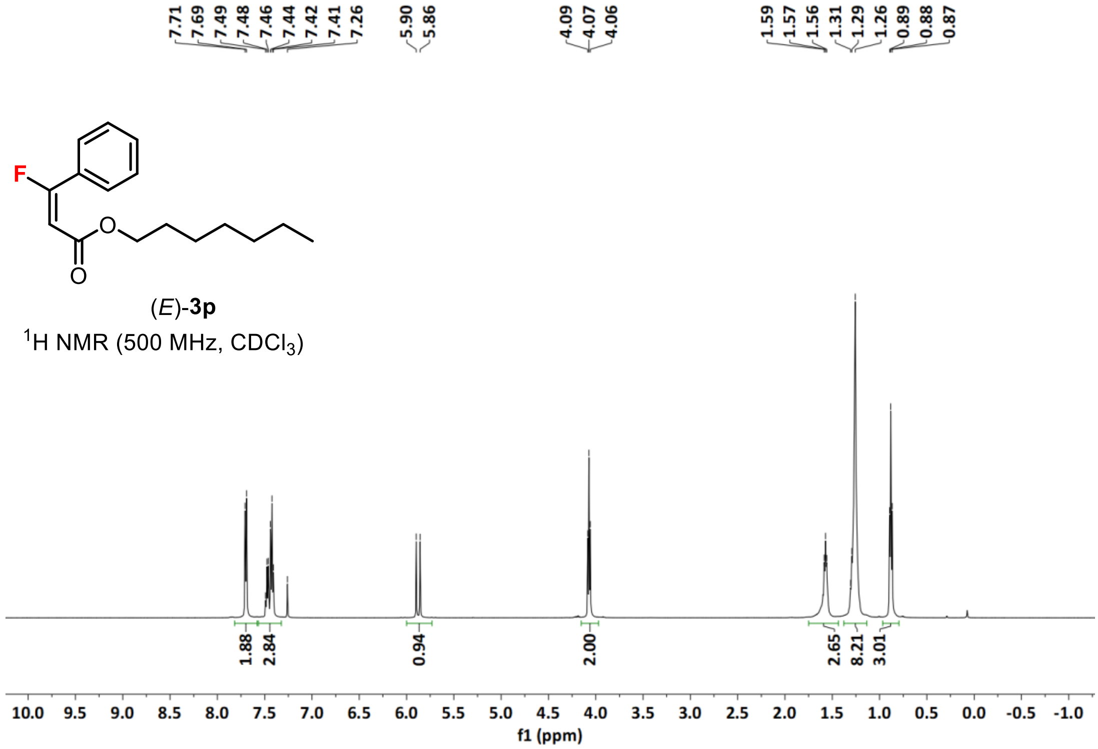

(*E*)-**3p**

^{1}H NMR (500 MHz, $CDCl_3$)

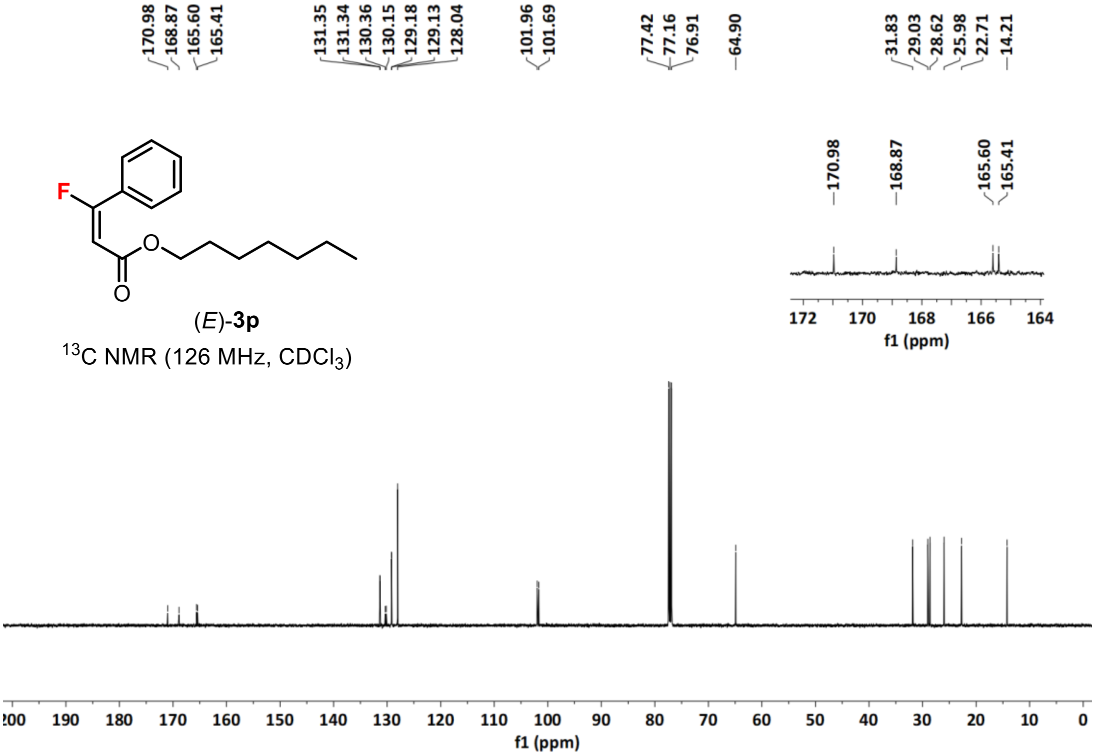
170.98
168.87
165.60
165.41
131.35
131.34
130.36
130.15
129.18
129.13
128.04
101.96
101.69
77.42
77.16
76.91
64.90
31.83
29.03
28.62
25.98
22.71
14.21
F
O
O
(*E*)-**3p**
^{13}C NMR (126 MHz, CDCl$_3$)
f1 (ppm)

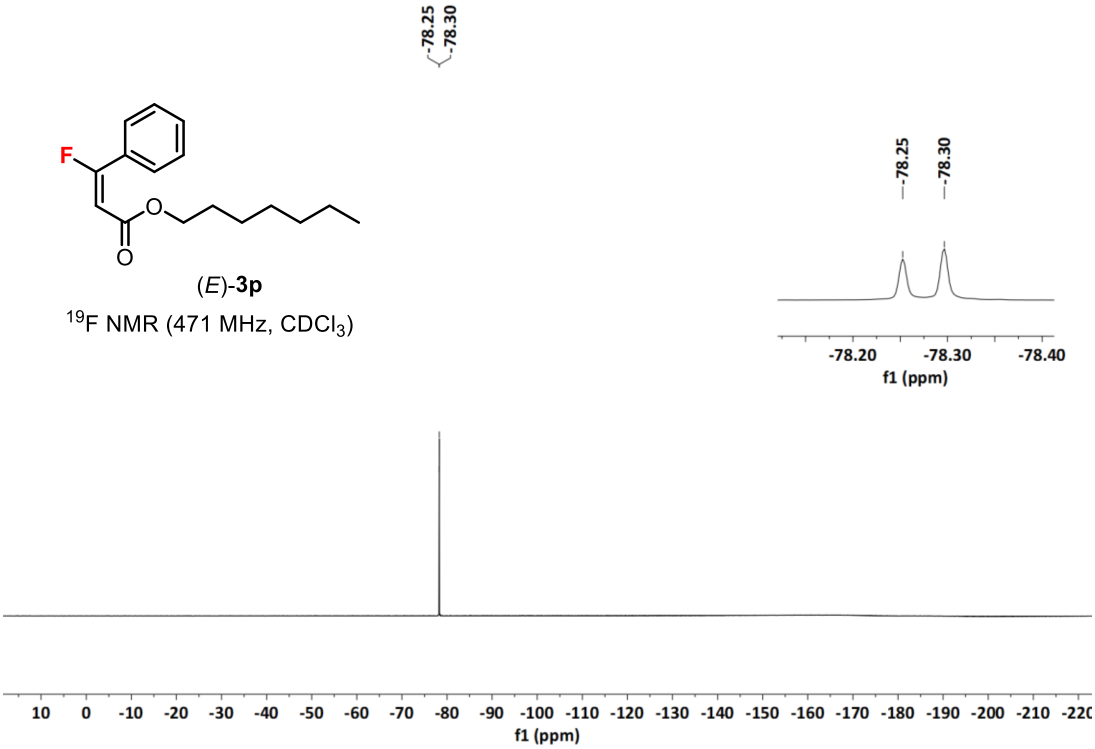
-78.25
-78.30
F
O
O
(*E*)-**3p**
^{19}F NMR (471 MHz, CDCl$_3$)
f1 (ppm)

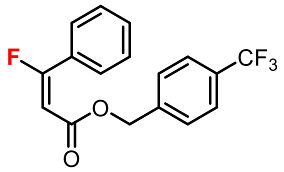

(*E*)-**3q**

^{1}H NMR (500 MHz, $CDCl_3$)

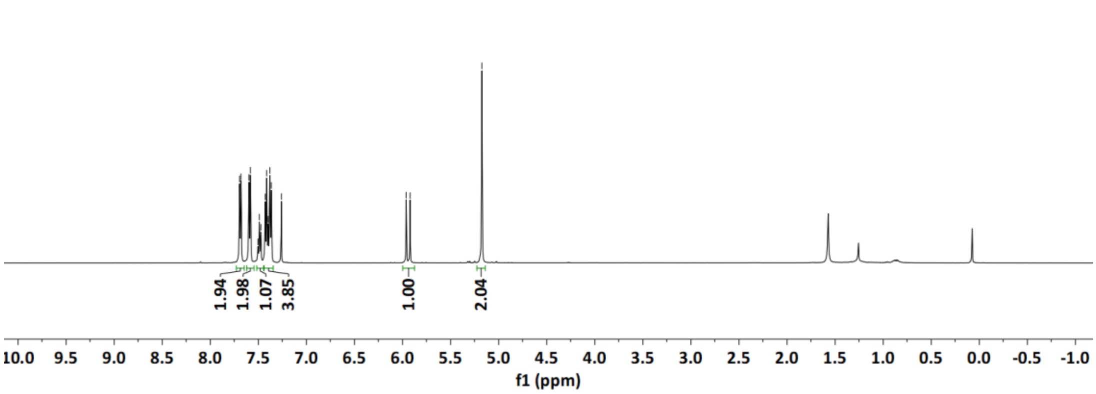

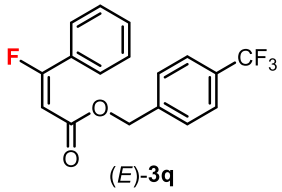

(*E*)-**3q**

^{13}C NMR (126 MHz, $CDCl_3$)

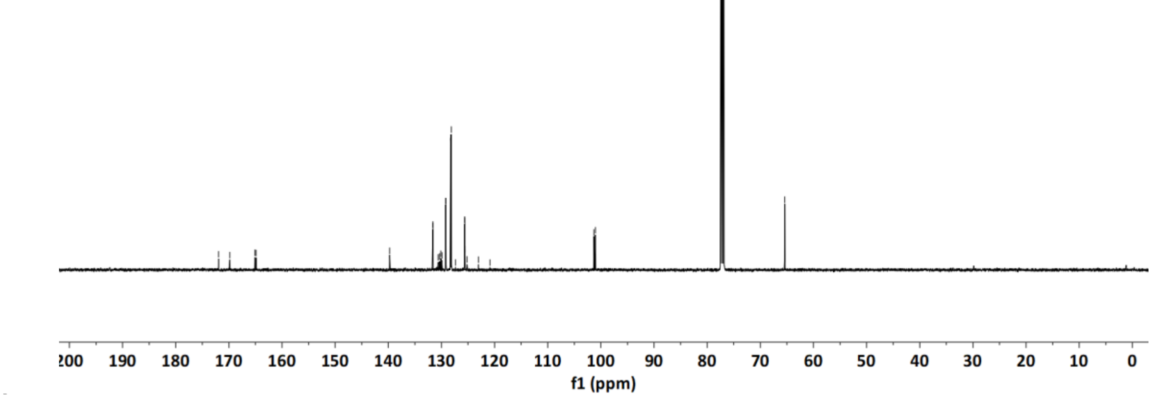

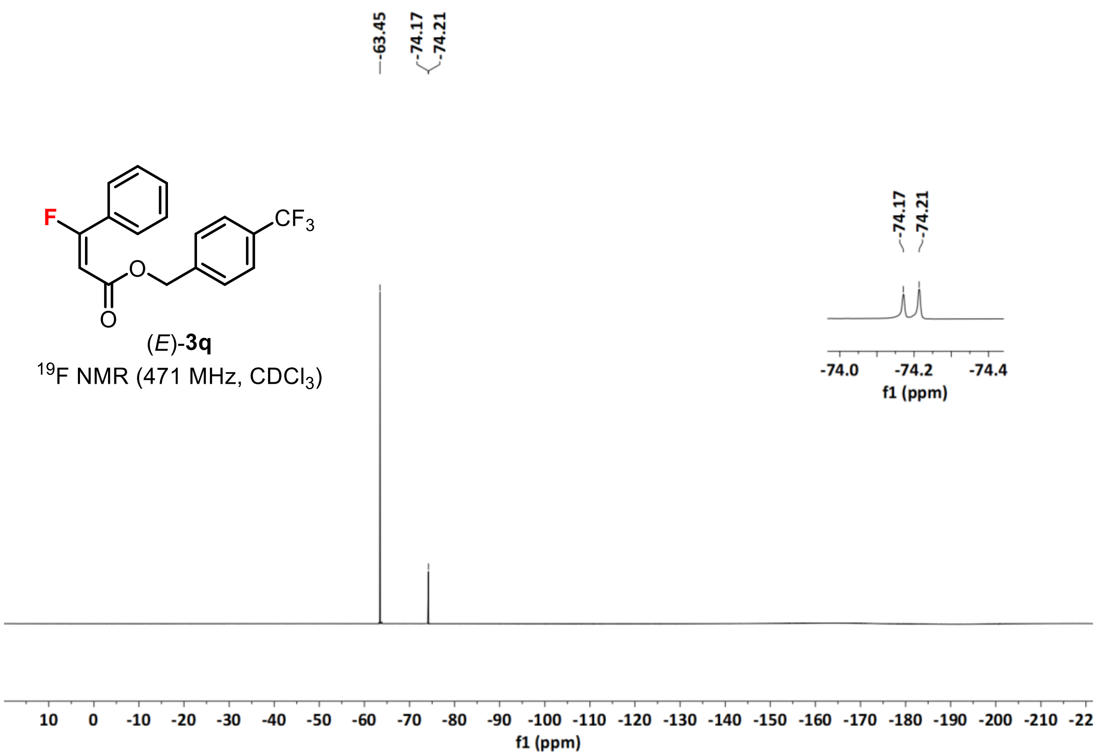

(*E*)-**3q**

^{19}F NMR (471 MHz, $CDCl_3$)

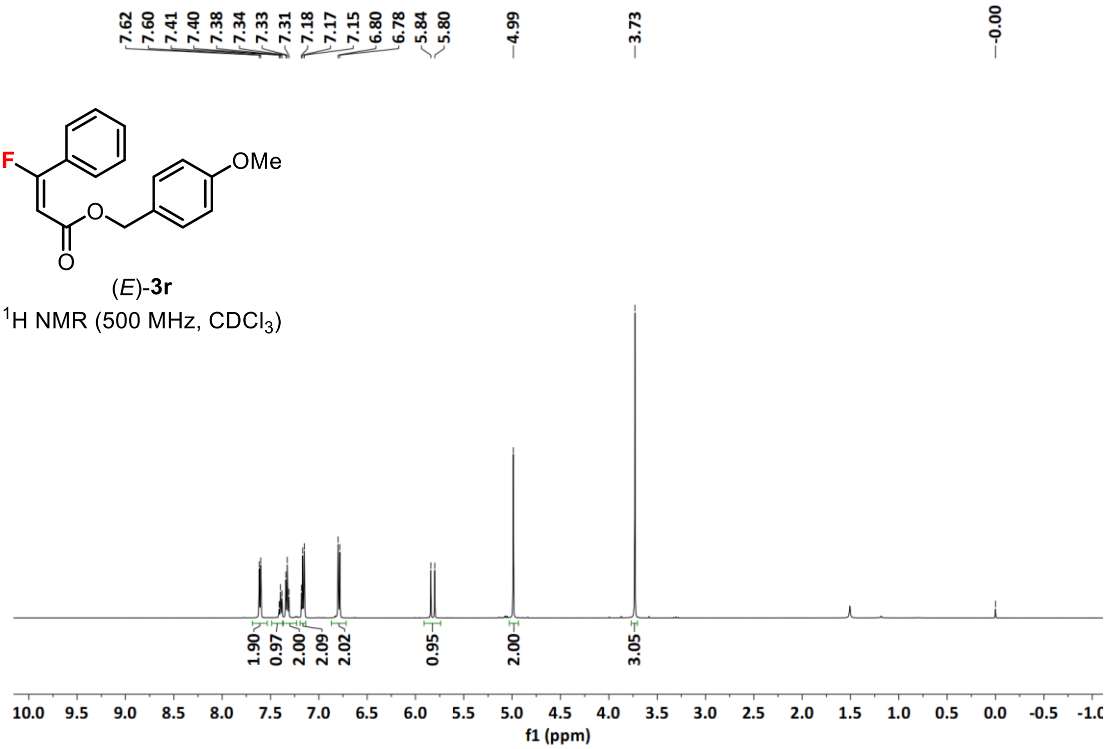

(*E*)-**3r**

^{1}H NMR (500 MHz, $CDCl_3$)

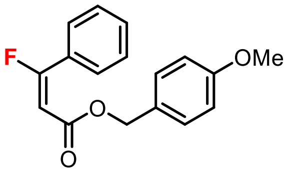

(*E*)-**3r**

^{13}C NMR (126 MHz, CDCl$_3$)

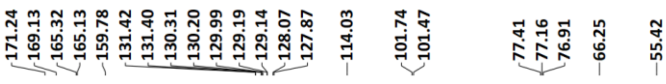
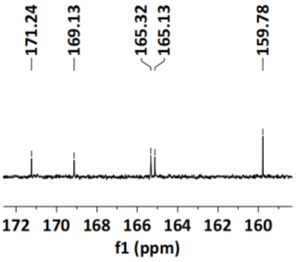
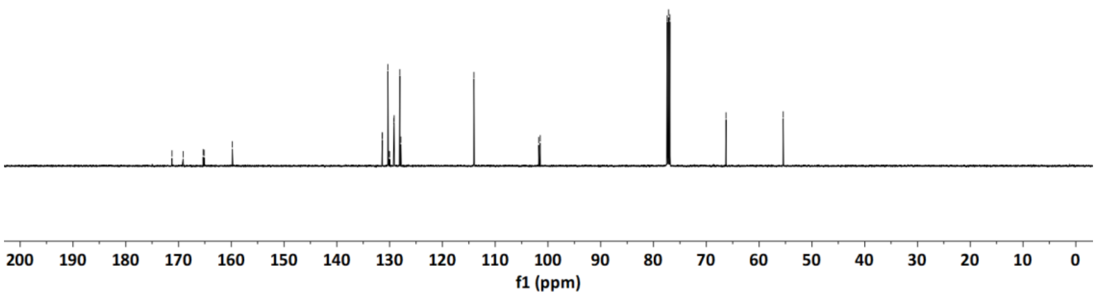

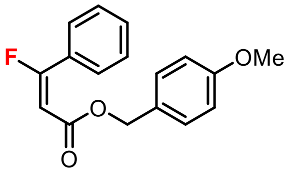

(*E*)-**3r**

^{19}F NMR (471 MHz, CDCl$_3$)

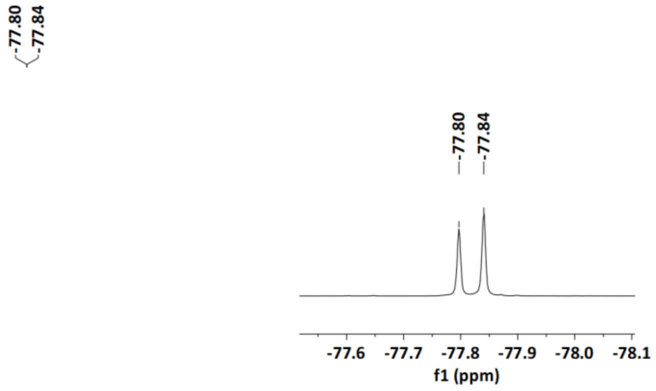
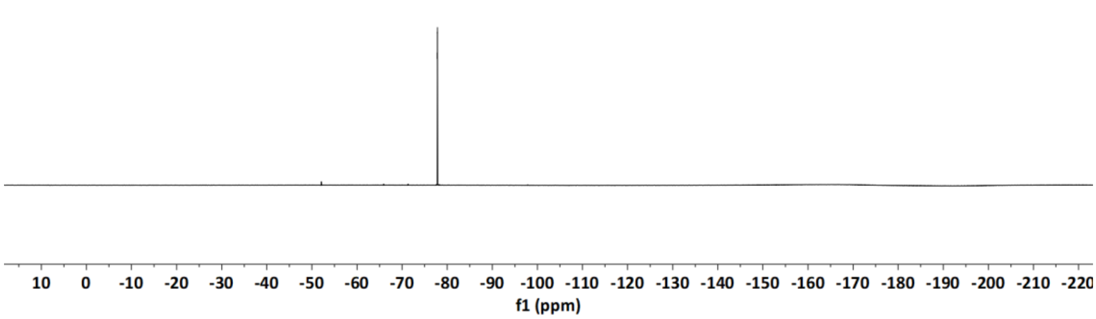

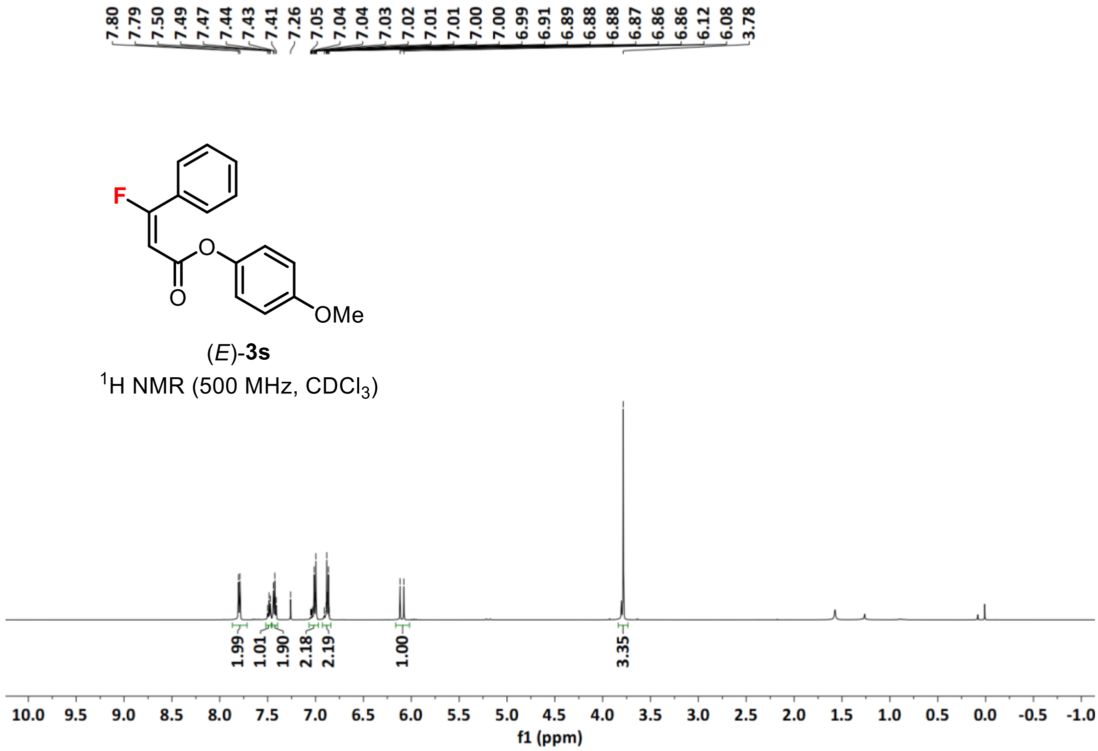

(*E*)-**3s**

^{1}H NMR (500 MHz, $CDCl_3$)

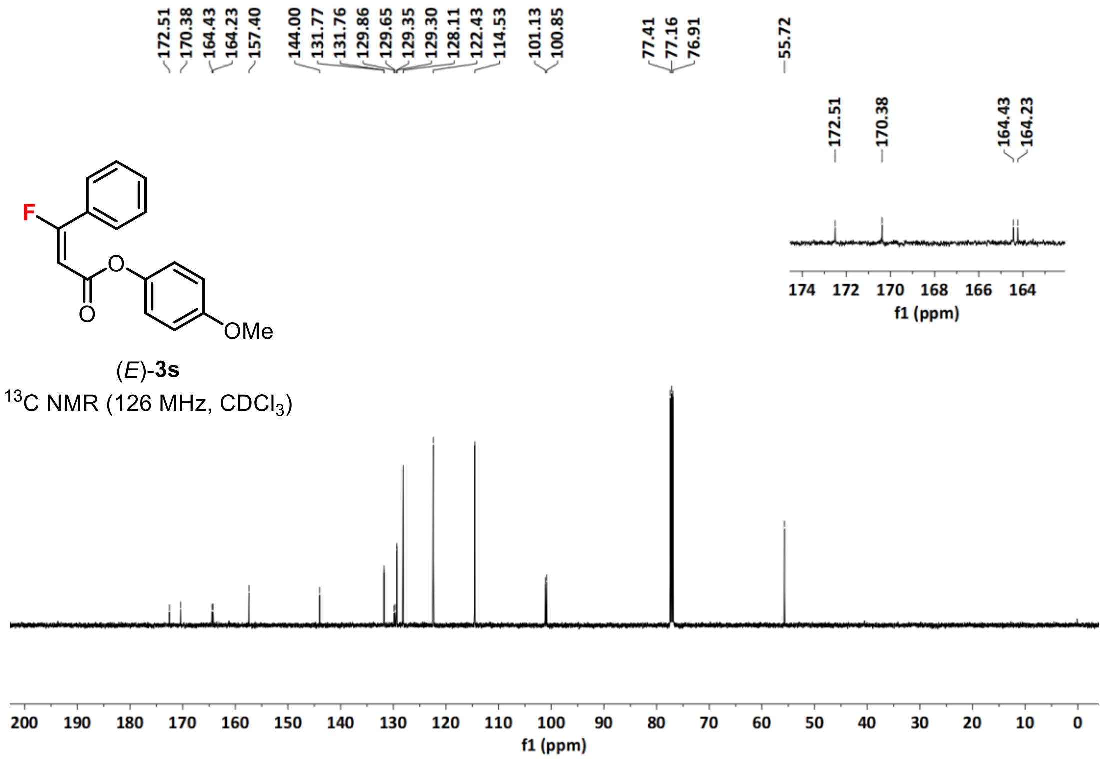

(*E*)-**3s**

^{13}C NMR (126 MHz, $CDCl_3$)

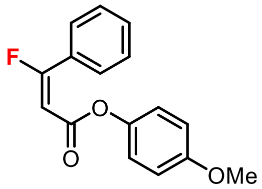

(*E*)-**3s**

^{19}F NMR (471 MHz, $CDCl_3$)

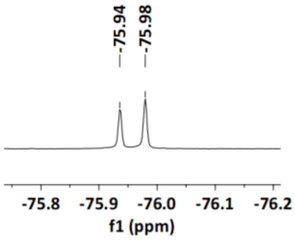

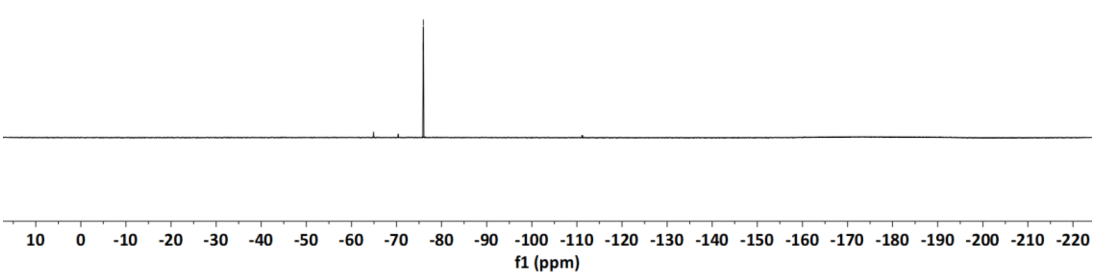

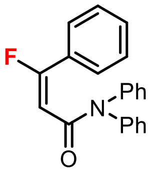

(*E*)-**5**

^{1}H NMR (500 MHz, $CDCl_3$)

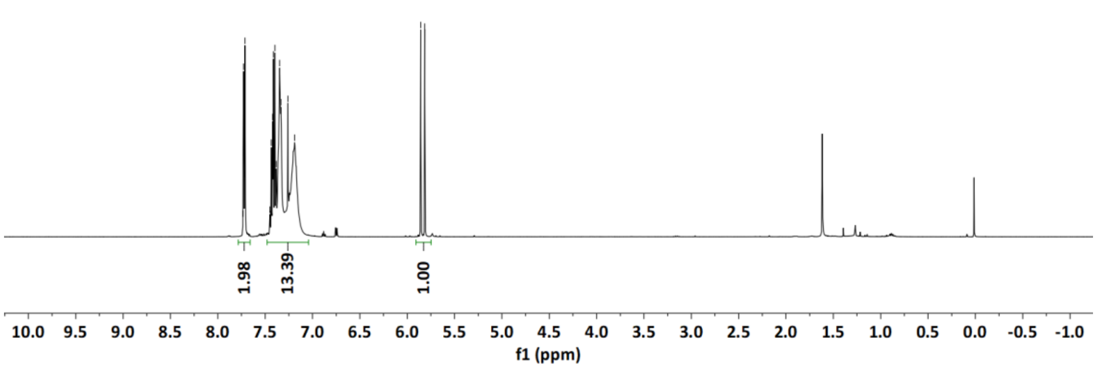

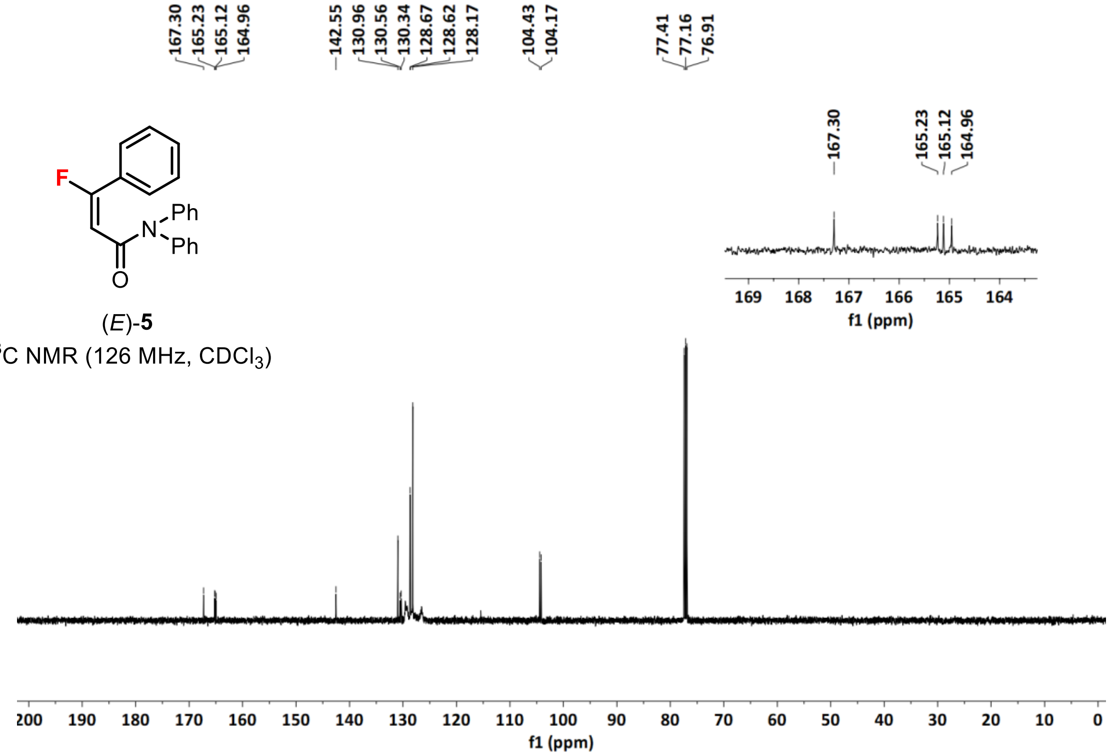

(*E*)-**5**

^{13}C NMR (126 MHz, CDCl$_3$)

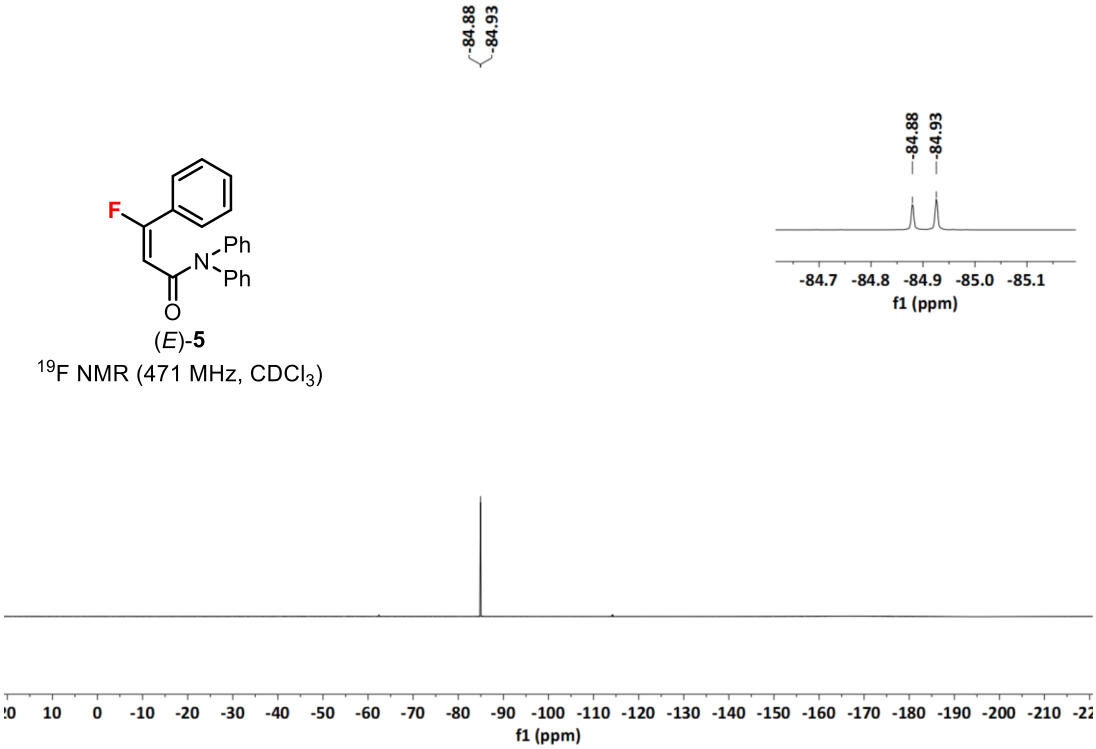

(*E*)-**5**

^{19}F NMR (471 MHz, CDCl$_3$)

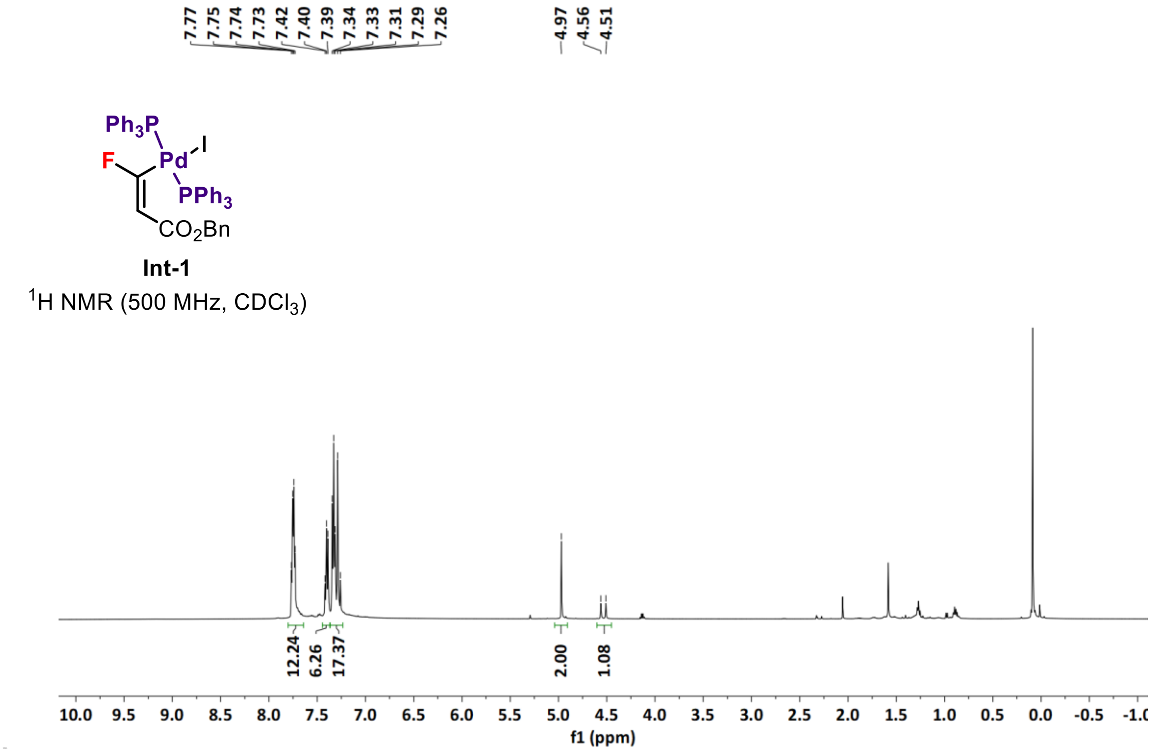
7.77
7.75
7.74
7.73
7.42
7.40
7.39
7.34
7.33
7.31
7.29
7.26
4.97
4.56
4.51
Ph3P
Pd
I
F
PPh3
CO2Bn
Int-1
1H NMR (500 MHz, CDCl3)
12.24
6.26
17.37
2.00
1.08
f1 (ppm)

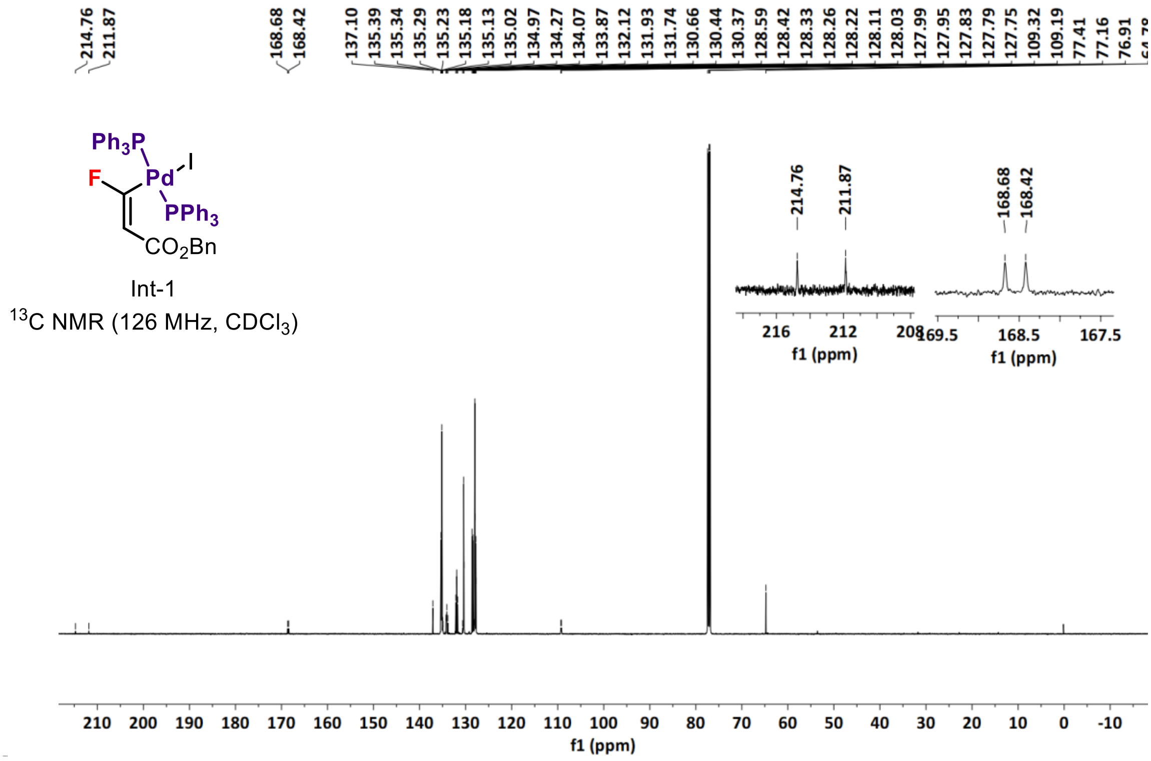
214.76
211.87
168.68
168.42
137.10
135.39
135.34
135.29
135.23
135.18
135.13
135.02
134.97
134.27
134.07
133.87
132.12
131.93
131.74
130.66
130.44
130.37
128.59
128.42
128.33
128.26
128.22
128.11
128.03
127.99
127.95
127.83
127.79
127.75
109.32
109.19
77.41
77.16
76.91
Ph3P
Pd
I
F
PPh3
CO2Bn
Int-1
13C NMR (126 MHz, CDCl3)
f1 (ppm)

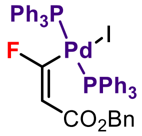

Int-1

^{19}F NMR (471 MHz, $CDCl_3$)

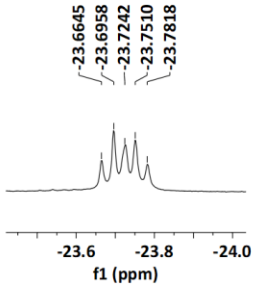

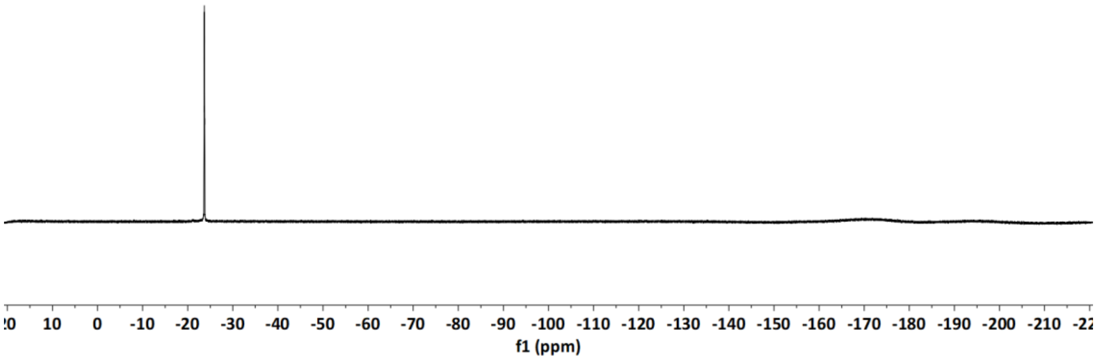

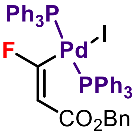

Int-1

^{31}P NMR (202 MHz, $CDCl_3$)

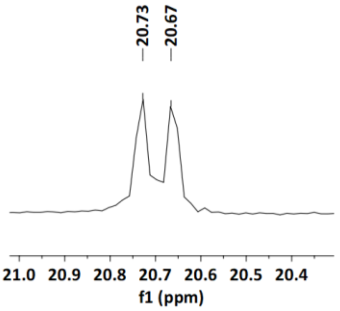

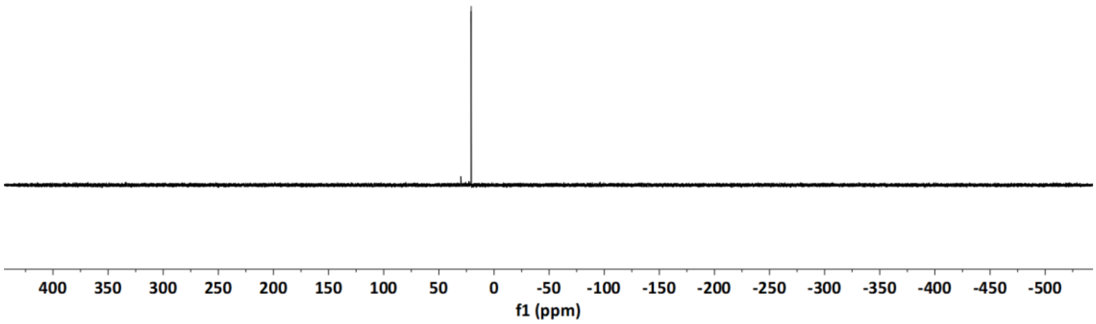

Supplement: Supplementary file 1 — ol4c02112_si_001.pdf [file ol4c02112_si_001.pdf]
